# Genome-Wide Identification and Comparison of Cysteine Proteases in the Pollen Coat and Other Tissues in Maize

**DOI:** 10.3389/fpls.2021.709534

**Published:** 2021-09-23

**Authors:** Yanhua Li, Liangjie Niu, Xiaolin Wu, Claudia Faleri, Fuju Tai, Man Zhang, Hui Liu, Wei Wang, Giampiero Cai

**Affiliations:** ^1^National Key Laboratory of Wheat and Maize Crop Science, College of Life Sciences, Henan Agricultural University, Zhengzhou, China; ^2^Department of Life Sciences, University of Siena, Siena, Italy

**Keywords:** cysteine protease, expression analysis, immunofluorescence microscopy, immunogold electron microscopy, gene duplication, maize pollen, phylogenetic analysis

## Abstract

Cysteine proteases, belonging to the C1-papain family, play a major role in plant growth and development, senescence, and immunity. There is evidence to suggest that pollen cysteine protease (CP) (ZmCP03) is involved in regulating the anther development and pollen formation in maize. However, there is no report on the genome-wide identification and comparison of *CPs* in the pollen coat and other tissues in maize. In this study, a total of 38 homologous genes of *ZmCP03* in maize were identified. Subsequently, protein motifs, conserved domains, gene structures, and duplication patterns of 39 *CPs* are analyzed to explore their evolutionary relationship and potential functions. The *cis*-elements were identified in the upstream sequence of 39 *CPs*, especially those that are related to regulating growth and development and responding to environmental stresses and hormones. The expression patterns of these genes displayed remarked difference at a tissue or organ level in maize based on the available transcriptome data in the public database. Quantitative reverse transcription PCR (RT-qPCR) analysis showed that *ZmCP03* was preferably expressed at a high level in maize pollen. Analyses by sodium dodecyl sulfate polyacrylamide gel electrophoresis (SDS-PAGE) and immunoblot, immunofluorescence and immunogold electron microscopy all validated the cellular localization of ZmCP03 in both the pollen coat and pollen cytoplasm. In addition, 142 *CP* genes from Arabidopsis (*Arabidopsis thaliana*), rice (*Oryza sativa*) and cotton (*Gossypium hirsutum*), together with 39 maize *CPs*, were retrieved to analyze their evolution by comparing with orthologous genes. The results suggested that ZmCP03 was relatively conservative and stable during evolution. This study may provide a referential evidence on the function of *ZmCP03* in pollen development and germination in maize.

## Introduction

The pollen coat is the outermost surface of pollen grains that contain the haploid male gametes and actively participates in the pollination and fertilization in plants (Zhang et al., 2016). Its composition mainly includes lipids, pigments, proteins, and aromatic compounds, which provide the pollen coat with a hydrophobic feature. Thus, the pollen coat can protect pollen grains from dehydration during pollen release and germination in the stigmatic surface (Edlund et al., 2004; Murphy, 2006; Quilichini et al., 2015; Wu et al., 2015; Zhang et al., 2016).

Previous studies in Brassica, Arabidopsis, and *Olea europaea* have showed that the pollen coat serves in pollen-stigmatic adhesion, recognition, and hydration and pollen germination through pollen coat-derived proteins, such as S-locus cysteine-rich protein 11 (SCR/SP11), pollen coat protein B class, extracellular lipase 4, and caleosin (Pacini and Hesse, 2004; Updegraff et al., 2009; Marshall et al., 2011; Zienkiewicz et al., 2011; Rejón et al., 2016; Wang et al., 2017). In particular, SCR/SP11 plays a vital role in regulating self-incompatibility in Brassica (Schopfer et al., 1999; Takayama et al., 2000; Marshall et al., 2011). Pollen coat protein B class (PCP-Bs) are key regulators of compatible pollen hydration in Arabidopsis. After knocking out *AtPCP-B* genes, pollen hydration was impaired and pollen tube growth was obviously delayed in *pcp-b* mutants (Wang et al., 2017). In maize, two pollen coat rich-proteins, β-glucanase and xylanase, have been proved to regulate pollen germination and tube growth (Suen et al., 2003; Suen and Huang, 2007). Pollen coat β-expansin 1 (Zea m 1) has been identified as a cell wall-loosening agent (Yennawar et al., 2006), and the pollen tubes of *expb1* mutant penetrated through the stigma and the style slower than the wild-type ones (Valdivia et al., 2007, 2009). Up to date, only a small number of pollen coat-derived proteins have been identified with definite functions in pollen-stigmatic interactions and pollen germination. Proteomic and bioinformatics analyses have implicated the important role of many functional-unknown pollen coat-derived proteins in maize (Gong et al., 2015; Wu et al., 2015) and rice (Dai et al., 2006). Recently, high-throughput sequencing and bioinformatic analyses have provided comprehensive perspectives in exploring the roles of pollen coat-derived proteins in cotton (Yang et al., 2020).

In the previous study, cysteine protease was found to be present at a high abundance in maize pollen coats (Wu et al., 2015). The enzyme, denoted as ZmCP03, belongs to the papain-like cysteine protease family (subfamily C1A) based on a domain structure analysis (Niño et al., 2020). Previously, ZmCP03 was found in the vacuoles of tapetum cells at a late stage of anther development (Li et al., 2012). Accumulating reports implicate that papain-like cysteine proteases play a role in tapetal programmed cell death (PCD) (Zhang et al., 2014; Shukla et al., 2019), biotic (Niño et al., 2020) and abiotic stresses (Pechan et al., 2000), and seed germination (Lu et al., 2015). For example, in Arabidopsis, a cysteine protease CEP1 has been found to participate in tapetal PCD and pollen development, while the *cep1* mutant and *cep1* overexpression both cause pollen infertility and abnormal tapetal PCD (Zhang et al., 2014).

At present, there is limited information on the role of *ZmCP03* in maize pollen development and tube germination, and especially a lack of the genome-wide identification and comparison of cysteine protease (CPs) in pollen coats and other maize tissues. The evolutionary relationships of the CPs in maize and in three other plant species were performed to provide insights into the biological functions of ZmCP03, especially in pollen development and germination.

## Materials and Methods

### Sequence Retrieval From Public Databases

The sequences of *ZmCP03* gene and its encoded protein were used as queries to identify the homologous genes and proteins in maize from the National Center for Biotechnology Information (NCBI) database (https://www.ncbi.nlm.nih.gov/gene/?term=zea%20mays; February 15, 2021; species, *zea mays*; 68,633 sequences) using basic logical alignment search tool (BLAST) searches. After removing the redundant and incomplete sequences, 39 unique gene and protein sequences of maize CPs were collected. The sequences of *CPs* in Arabidopsis (*Arabidopsis thaliana*) were collected from the TAIR v.10 database (https://www.arabidopsis.org; February 15, 2021) (Richau et al., 2012), those for rice (*Oryza sativa*) from the NCBI database (https://www.ncbi.nlm.nih.gov/gene/?term=rice; February 15, 2021; species, *Oryza sativa*; 80,075 sequences) (Wang et al., 2018), and those for cotton (*Gossypium hirsutum*) from Cottongene (https://www.cottongen.org/organism/Gossypium/hirsutum; July 15, 2021; species, *Gossypium hirsutum*; 12,877,329 sequences) (Zhang et al., 2019). The definitions of the CP domains were based on a Pfam (PF00112) analysis (http://pfam.xfam.org/), and the domains of maize CPs were examined by Web CD-Search Tool (https://www.ncbi.nlm.nih.gov/Structure/bwrpsb/bwrpsb.cgi) (Marchler-Bauer et al., 2017).

### Gene Structure and Protein Sequence Analysis of *ZmCP* Genes

The structure of the *ZmCP* genes was created by the Gene Structure Display Server 2.0 (GSDS; http://gsds.gao-lab.org/) (Hu et al., 2015). The physiochemical properties of ZmCPs, especially theoretical isoelectric point (pI), molecular weights (MW), instability index, and grand average of hydropathy (GRAVY) values, were calculated by the ExPASy Protparam tool (https://web.expasy.org/protparam) (Artimo et al., 2012; Qanmber et al., 2019b).

### Phylogenetic Tree Construction, Chromosomal Distribution, and Collinearity Analysis of *ZmCP* Genes

The proteins sequences of 39 ZmCPs were aligned using “align by muscle” and the unrooted phylogenetic tree was created by MEGA X with neighbor-joining (NJ) algorithm. The parameters were set on 1,000 replicates for bootstrapping, p-distance, and 50% partial deletion site coverage cut-off (Kumar et al., 2018). The position information of *ZmCPs* on chromosomes was obtained from a gff3-file of maize genome annotation data (https://www.ncbi.nlm.nih.gov/genome/?term=maize) and presented using the TBtools toolkit (Chen et al., 2020). The collinear pairs of the *CP* genes were identified using MCScanX (Wang et al., 2012) and the collinearity map was built using the TBtools toolkit (Chen et al., 2020).

### Motif Analysis of ZmCPs and Promoter *cis*-elements Analysis of *ZmCPs*

The protein motifs were analyzed using the MEME (Multiple EM for Motif Elicitation) tool (https://meme-suite.org/tools/meme) (Bailey et al., 2015). The parameters were set as follows: the motif width: 6–80 amino acids; the motif maximum number: 15 (Niu et al., 2020). The 2,000 bp upstream sequence regions of 39 *ZmCP* genes were collected to analyze the promoter *cis*-elements using the database PlantCARE (http://bioinformatics.psb.ugent.be/webtools/plantcare/html/) (Lescot et al., 2002).

### Calculation of *Ka*/*Ks* Values

The number of synonymous (*Ks*) and non-synonymous (*Ka*) substitutions per site of duplicated cystatin genes were calculated by TBtools (Chen et al., 2020). *Ka/Ks* < 1 means negative selection, *Ka/Ks* = 1 means neutral selection, and *Ka/Ks* > 1 means positive selection.

### Expression Profiles of the *ZmCP* Genes in Different Tissues

Tissue-specific expression data were collected from MaizeGDB (https://www.maizegdb.org). The expression abundances were determined for replicated mRNA-sequencing datasets from 79 tissues and abiotic (salt stress, drought, and temperature stress) or biotic stress treatments (*Colletotrichum graminicola* and *Cercosporazeina* infection). The transcript abundances of *ZmCPs* were calculated as fragments per kilobase per million mapped fragments (FPKM) in an unstranded mode, a maximum intron length of 60 kb, and utilizing reference bias correction (Hoopes et al., 2019). The heatmap of the tissue-specific expression was created using expression values in different tissues by TBtools (Chen et al., 2020).

### RNA Extraction and Quantitative Reverse Transcription PCR (RT-qPCR)

The root, stem, and leaves of maize inbred line B104 were sampled at the three-leaf seedling stage, and pollen grains were sampled at the pollen shed stage. Total RNA was extracted using RNA-Solv^®^ reagent (Omega Bio-Tek, Norcross, GA, USA). Single-stranded cDNA was synthesized using a 5 × All-In-One MasterMix with an AccuRT Genomic DNA Removal Kit (Applied Biological Materials Inc., Richmond, Canada) according to the instructions of the manufacturer.

The gene-specific primers of *ZmUBI* and *ZmCP03* were designed using the Primer Premier 5.0 software (http://www.premierbiosoft.com/) and commercially synthesized (Biomed Cooperation, Beijing, China) as follows: *ZmUBI*, 5′-TAAGCTGCCGATGTGCCTGCG-3′, and 5′-CTGAAAGACAGAACATAATGAGCACAG-3′; *ZmCP03*, 5′-AAGAAGCGGGCCAACGTATC-3′, and 5′-CCCTGTCGTGATCTTGGTGA-3′. The *ZmUBI* was used as an internal control to normalize the data.

A quantitative reverse transcription PCR (RT-qPCR) was carried out using the StepOnePlus™ Real-Time PCR Instrument Thermal Cycling Block (Applied Bio-systems). The PCR conditions contained an initial denaturation step at 95°C for 5 min, followed by 40 cycles at 95°C for 10 s and 60°C for 30 s. The quantification method 2^−ΔΔCT^ (Livak and Schmittgen, 2001) was used to assess the relative expression level of *ZmCP03* in different maize tissues. Data were presented as relative expression (mean ± SD) from three biological replicates after normalization based on *ZmUBI* expression. Significant differences in expression changes among tissues were analyzed by Student's *t*-test (^*^p < 0.05, ^**^p < 0.01) in the GraphPad Prism 8.0 software (San Diego, CA, USA).

### Preparation of Monoclonal Antibodies and Immunoblot Analysis

A 15 amino acid polypeptide, PVRRDAGKKRANVSS, from N_115_ to N_130_ of ZmCP03 was used to prepare anti-ZmCP03 antibodies. The synthesized peptide was sequenced by a matrix assisted laser desorption ionization-time of flight (MALDI-TOF) mass spectrometry analysis to verify its sequence. Mice were sequentially immunized on days 1, 15, 35, and 56 with the synthesized peptide. Splenocytes were harvested from mice on day 61 and fused with syngeneic myeloma cells to form hybridoma cells, which were selectively cultured. The positive hybridoma cells were then selected and clonally expanded before inoculation into the peritoneal cavity of mice. After 1–2 weeks, mouse ascites was removed and monoclonal antibodies were immunoaffinity purified.

As for immunoblot analysis, protein samples were subjected to sodium dodecyl sulfate polyacrylamide gel electrophoresis (SDS-PAGE) (12.5% gel), and electrophoretically transferred onto hydrophilic polyvinylidene fluoride (PVDF) membranes (Hybond-P, Amersham, Uppsala, Sweden). After blocking with bovine serum proteins, the blots were incubated with anti-ZmCP03 (1:5000 dilution) for 1 h. After rinsing, it was incubated with rabbit anti-mouse IgG (1:2000 dilution) labeled with horseradish peroxidase for 1 h and stained by Amersham^TM^ ECL^TM^ prime kit (Cytiva, Marlborough, MA, USA).

### Immunofluorescence Microscopy

Pollens were fixed in paraformaldehyde, rinsed, dehydrated, and resin embedded. Sections (150-nm thick) were cut and placed on drops of water on glass slides and dried at 45°C. Sections were blocked with bovine serum albumin (BSA) and incubated with anti-ZmCP03 (1:100 dilution) for 1 h. After washing, sections were incubated with rabbit anti-mouse antibody labeled with fluorescein for 1 h, rinsed, and observed under a fluorescence microscope (Nikon C2-ER; Nikon Corp, Tokyo, Japan).

### Immunogold Electron Microscopy

The immunogold electron microscopy localization of ZmCP03 was carried out as in our previous report (Wang et al., 2006). Briefly, pollen grains were fixed with paraformaldehyde, rinsed, dehydrated, and resin embedded. Ultrathin sections (80-nm thick) were blocked with bovine serum proteins and incubated with anti-ZmCP03 (1:100 dilution) for 1 h. After rinsing, it was incubated with gold particles (10 nm) conjugated with rabbit anti-mouse IgG for 1 h. Sections were sequentially negatively stained with uranyl acetate (10 min) and lead citrate (5 min), rinsed, and examined by transmission electron microscopy (Hitachi HT-7700, Tokyo, Japan).

## Results

### Biophysical Properties of CPs in Maize

The information about the protein length, MW, pI, and GRAVY values of 39 maize CP proteins is listed in Table 1, and the nucleotide and amino acid sequences are provided in Supplementary Dataset 1. Based on the instability index, 31 of 39 CPs were stable, whereas the others, including ZmCP03, were instable. Besides, their pIs ranged from 4.73 to 8.36, and eight CPs were alkaline proteins (pI > 7). All of the 39 CPs were hydrophilic with negative GRAVY values.

**Table 1:** Physiochemical properties of the identified ZmCP proteins in maize.

**No**	**Gene name**	**Protein name**	**Protein ID**	**Family**	**Protein size**	**mW (Da)**	**pI**	**Instability index**	**GRAVY**
1	*ZmCP01*	Uncharacterized protein LOC100382061 precursor	NP_001355124.1	C1	355	39,029	7.64	40.88	−0.472
2	*ZmCP02*	Low quality protein: ervatamin-B	XP_008653318.3	C1	373	40,870	8.36	38.08	−0.422
3	*ZmCP03*	Cysteine protease	NP_001146834.1	C1	352	38,089	8.02	51.56	−0.209
4	*ZmCP04*	Thiol protease SEN102	NP_001150062.1	C1	349	38,111	7.66	31.93	−0.344
5	*ZmCP05*	Ervatamin-B	XP_008660699.2	C1	359	39,515	6.96	33.53	−0.379
6	*ZmCP06*	Ervatamin-B	XP_020400118.1	C1	347	38,151	7.57	33.25	−0.522
7	*ZmCP07*	Cysteine proteinases superfamily protein	XP_008662986.1	C1	390	43,331	5.82	42.92	−0.454
8	*ZmCP08*	Thiol protease SEN102 precursor	NP_001356222.1	C1	374	39,956	6.33	40.50	−0.258
9	*ZmCP09*	Vignain	NP_001148894.2	C1	356	38,140	6.83	32.93	−0.165
10	*ZmCP10*	Cysteine proteinase 1 precursor	NP_001358577.1	C1	371	40,303	6.01	30.76	−0.304
11	*ZmCP11*	Cysteine proteinase 2 precursor	NP_001105479.2	C1	360	38,959	7.55	23.57	−0.131
12	*ZmCP12*	Senescence-specific cysteine protease SAG12	NP_001130503.1	C1	354	37,634	4.73	20.78	−0.245
13	*ZmCP13*	Senescence-specific cysteine protease SAG12	NP_001140922.1	C1	354	37,674	4.80	20.40	−0.248
14	*ZmCP14*	Vignain	NP_001141984.1	C1	362	38,849	4.87	26.31	−0.291
15	*ZmCP15*	Cysteine protease 1 isoform X1	XP_035823976.1	C1	340	35,812	8.37	30.79	−0.114
16	*ZmCP16*	Senescence-specific cysteine protease SAG39	XP_008663073.1	C1	352	37,723	4.73	25.36	−0.291
17	*ZmCP17*	Senescence-specific cysteine protease SAG39	XP_020398957.1	C1	343	37,085	5.73	21.43	−0.196
18	*ZmCP18*	Senescence-specific cysteine protease SAG39	XP_008669668.1	C1	343	37,253	5.34	28.42	−0.221
19	*ZmCP19*	Senescence-specific cysteine protease SAG39	XP_008669672.3	C1	307	33,778	5.15	24.17	−0.424
20	*ZmCP20*	Senescence-specific cysteine protease SAG39	XP_008669676.1	C1	347	37,191	5.35	13.98	−0.223
21	*ZmCP21*	Senescence-specific cysteine protease SAG39	XP_020395776.1	C1	340	36,855	5.03	17.02	−0.307
22	*ZmCP22*	Senescence-specific cysteine protease SAG39	XP_008659446.1	C1	340	36,675	5.09	20.38	−0.296
23	*ZmCP23*	Senescence-specific cysteine protease SAG39	XP_008659445.1	C1	340	36,754	5.09	21.10	−0.268
24	*ZmCP24*	Cysteine protease XCP1	NP_001150119.1	C1	385	41,596	4.99	42.46	−0.420
25	*ZmCP25*	Cysteine protease5	NP_001149806.1	C1	377	41,174	5.08	30.16	−0.398
26	*ZmCP26*	Cysteine protease XCP1	XP_008646166.1	C1	359	39,129	6.16	31.66	−0.236
27	*ZmCP27*	Xylem bark cysteine peptidase 3	NP_001141813.1	C1	460	48,740	6.55	41.87	−0.198
28	*ZmCP28*	Maize insect resistance 2 precursor	NP_001104878.2	C1	493	53,020	5.56	41.58	−0.402
29	*ZmCP29*	Cysteine protease Mir1	NP_001105571.1	C1	398	42,624	5.05	35.59	−0.356
30	*ZmCP30*	Putative cysteine protease RD21B	NP_001357489.1	C1	465	49,914	5.75	30.56	−0.308
31	*ZmCP31*	Cysteine protease 1 precursor	NP_001150196.1	C1	470	50,228	5.47	30.15	−0.260
32	*ZmCP32*	Insect resistance3	NP_001105993.2	C1	465	50,366	5.07	32.62	−0.405
33	*ZmCP33*	Cysteine protease 1 precursor	NP_001149658.2	C1	469	50,759	5.04	37.16	−0.411
34	*ZmCP34*	Uncharacterized protein LOC100272949 precursor	NP_001140873.2	C1	379	41,080	5.90	35.14	−0.419
35	*ZmCP35*	KDEL-tailed cysteine endopeptidase CEP1	NP_001130571.2	C1	368	17,874	6.47	36.63	−0.434
36	*ZmCP36*	KDEL-tailed cysteine endopeptidase CEP1	NP_001309915.1	C1	384	42,087	6.28	43.53	−0.612
37	*ZmCP37*	Uncharacterized protein LOC100284911	NP_001151278.2	C1	377	29,969	5.92	32.62	−0.437
38	*ZmCP38*	Cysteine proteinases superfamily protein	XP_008671849.1	C1	376	40,720	7.48	32.89	−0.173
39	*ZmCP39*	Uncharacterized protein	NP_001136636.1	C1	361	39,383	6.37	39.51	−0.156

### Evolutionary Analysis of *CP* Genes in Selected Plant Species

A total of 142 CP genes were identified in three plant species (Arabidopsis, cotton, and rice) as papain-like cysteine proteases. Among them, 31 were obtained from Arabidopsis (Richau et al., 2012), 78 from cotton (Zhang et al., 2019), and 33 from rice (Wang et al., 2018).

**Figure 1:**
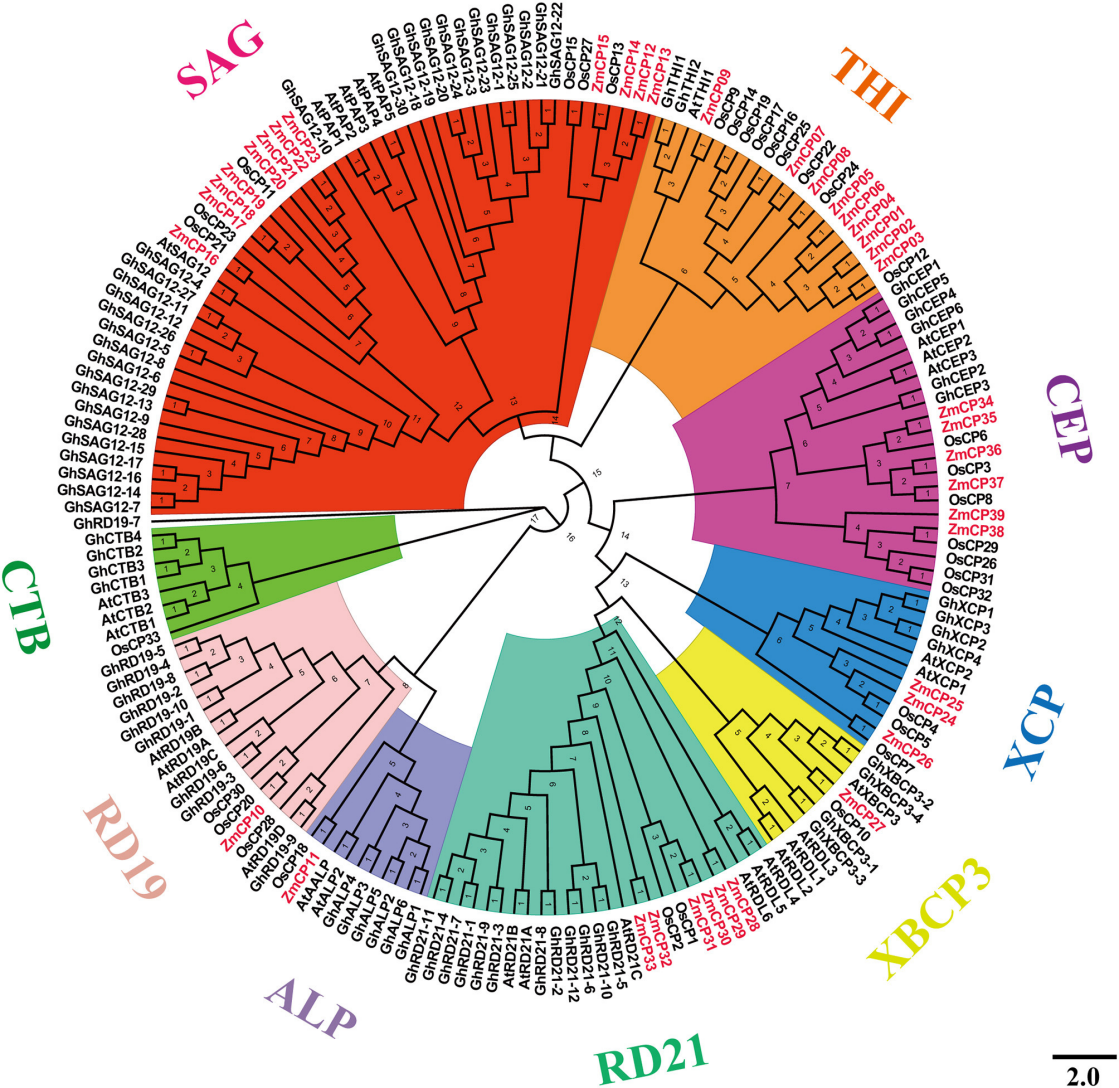
Evolutionary relations among 181 cysteine protease *(CP)* genes among the four plant species examined.

To examine the evolutionary events in the four plant species, a phylogenetic tree was created by MEGA X (Figure 1). The tree was divided into nine subfamilies, similar to the presentation of CPs in cotton (Zhang et al., 2019), namely, the subfamilies responsive to desiccation 21 (RD21), cysteine endopeptidase (CEP), xylem cysteine peptidase (XCP), xylem bark cysteine peptidase 3 (XBCP3), Th1 immune response-associated cysteine protease (THI), senescence-associated gene 12 (SAG12), responsive to desiccation19 (RD19), aleurain-like protease (ALP), and cathepsin B-like (CTB). Therefore, the present study used the same nomenclature as previous studies (Richau et al., 2012; Zhang et al., 2019).

The SAG12 is the largest subfamily, whereas the subfamily XBCP3 had the fewest members (only eight). Previously, GhRD19-7 was in the same group with GhRD19-9 (Zhang et al., 2019), but it fell in the clade with GhRD19-9 in the results, possibly because the phylogenetic tree was built using four different plant species here, whereas the tree in the previous report (Zhang et al., 2019) was created using cotton and Arabidopsis.

### Evolutionary Analysis, Motif Composition, and Gene Structure of *ZmCPs*

**Figure 2:**
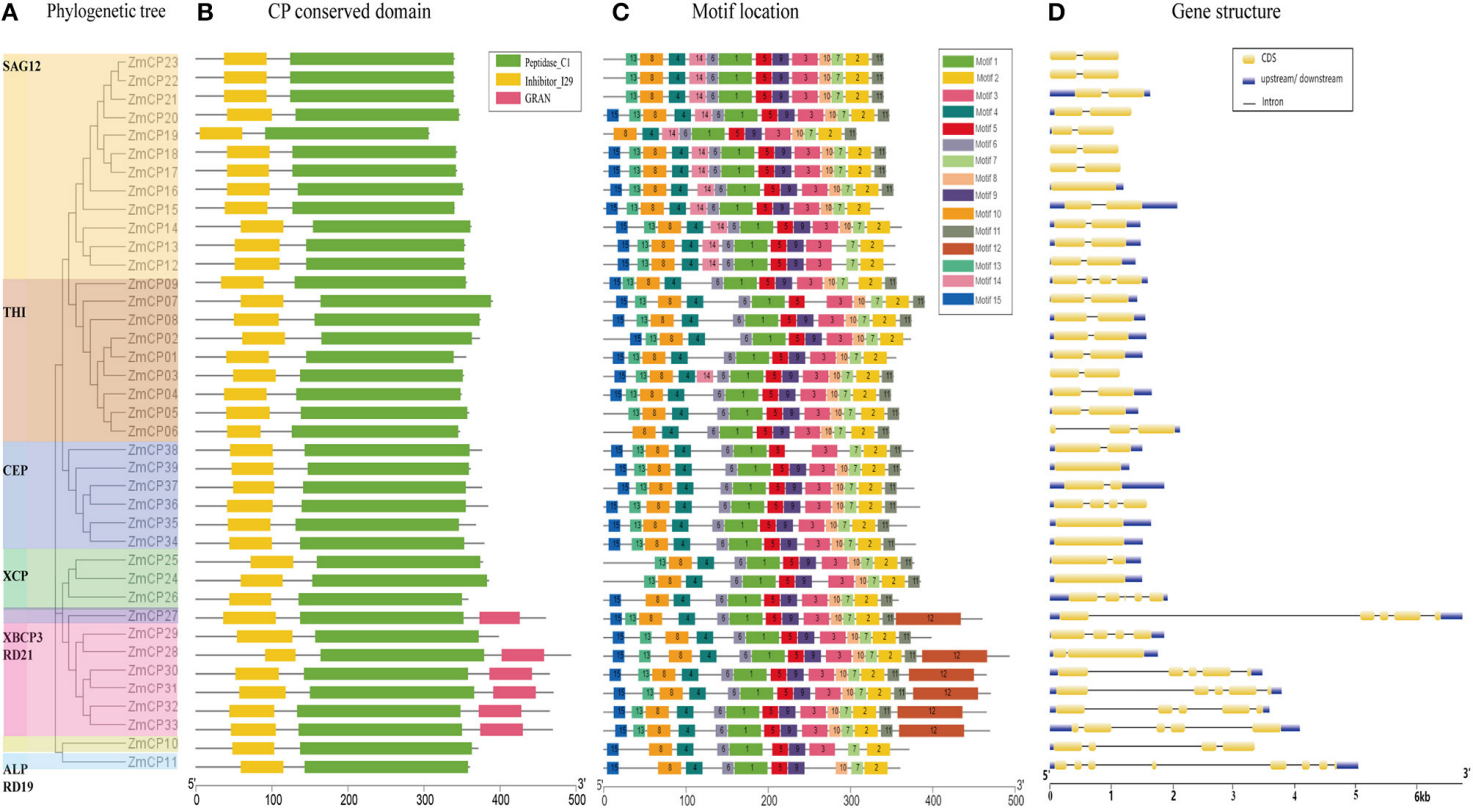
Evolutionary analysis, motif compositions, and gene structures of 39 *ZmCPs*. **(A)** The unrooted phylogenetic tree. **(B)** The conversed domain in *ZmCPs*. **(C)** The motif location in ZmCPs. **(D)** The gene structures of 39 *ZmCPs*.

To reveal the evolutionary relationship of the *ZmCPs*, a multiple sequence alignment of 39 maize CP proteins was carried out and used to construct an unrooted phylogenetic tree. As a result, the ZmCPs were divided into nine groups, namely, SAG12, THI, CEP, XCP, XBCP3, RD21, ALP, and RD19 (Figure 2A). Group SAG12 was composed of 12 ZmCPs, group THI had 9 members (including ZmCP03), group CEP had 6 members, while group XBCP3, ALP, and RD19 all had only one member. A similar phylogenetic tree was created using the maximum likelihood method (Supplementary Figure 1).

Furthermore, the conserved domains of ZmCPs were visualized (Figure 2B). All ZmCPs contain the peptidase C1 domain, which is the known typical domain of peptidase family C1 (Rawlings et al., 2010). All ZmCPs also contained the inhibitor I29 domain (MEROPS peptidase inhibitor family I29). The I29 domain is also found at the N-terminus of a variety of peptidase precursors that belong to the MEROPS peptidase subfamily C1A (Groves et al., 1996). It forms an α-helical domain that runs through the substrate-binding site and prevents access. The removal of this region by proteolytic cleavage results in the activation of the enzyme (Rawlings et al., 2010). Besides, six ZmCP proteins (ZmCP27, 28, 30–33) from the subfamily XCPB3 and RD21 had a C-terminal extension domain named the GRAN domain (granulins). Granulins were first found in animals as growth hormones released after wounding events (Bateman and Bennett, 2009; Wang et al., 2018).

Fifteen different motifs with variable amino acid lengths and sequences were identified in 39 ZmCPs by the MEME search tool (https://meme-suite.org/tools/meme) (Figure 2C). Of them, motifs 1, 2, 6, and 7 existed in all examined ZmCPs, forming the catalytic triad Cys-His-Asn. The 38 ZmCPs, except for ZmCP37, possessed motifs 4 and 8, which were the conversed domain inhibitor I29 (Wang et al., 2018). The sequence of motif 8 is ExxxRxxxFxxNxxxI/VxxxN with one mismatch. Besides, motif 15 was special to the subfamily CEP except for ZmCP37. The sequence of motif 12 is Cx5Cx5CCCx7Cx4CCx6CCx5CCx6Cx6C (the GRAN domain), which existed in the C-terminal extensions of XBCP3 and five members from RD21.

The gene structure analysis showed that 5 genes contained no intron, 21 (most belonging to the subfamilies SAG12 and THI) contained only a single intron, and the remaining 13 contained 2–7 introns (Figure 2D). Obviously, the structures of the 39 *ZmCP* genes were similar to those reported in rice (Wang et al., 2018).

### Enrichment Analysis of *cis*-elements Motifs in the Promoters of *ZmCP* Genes

**Figure 3:**
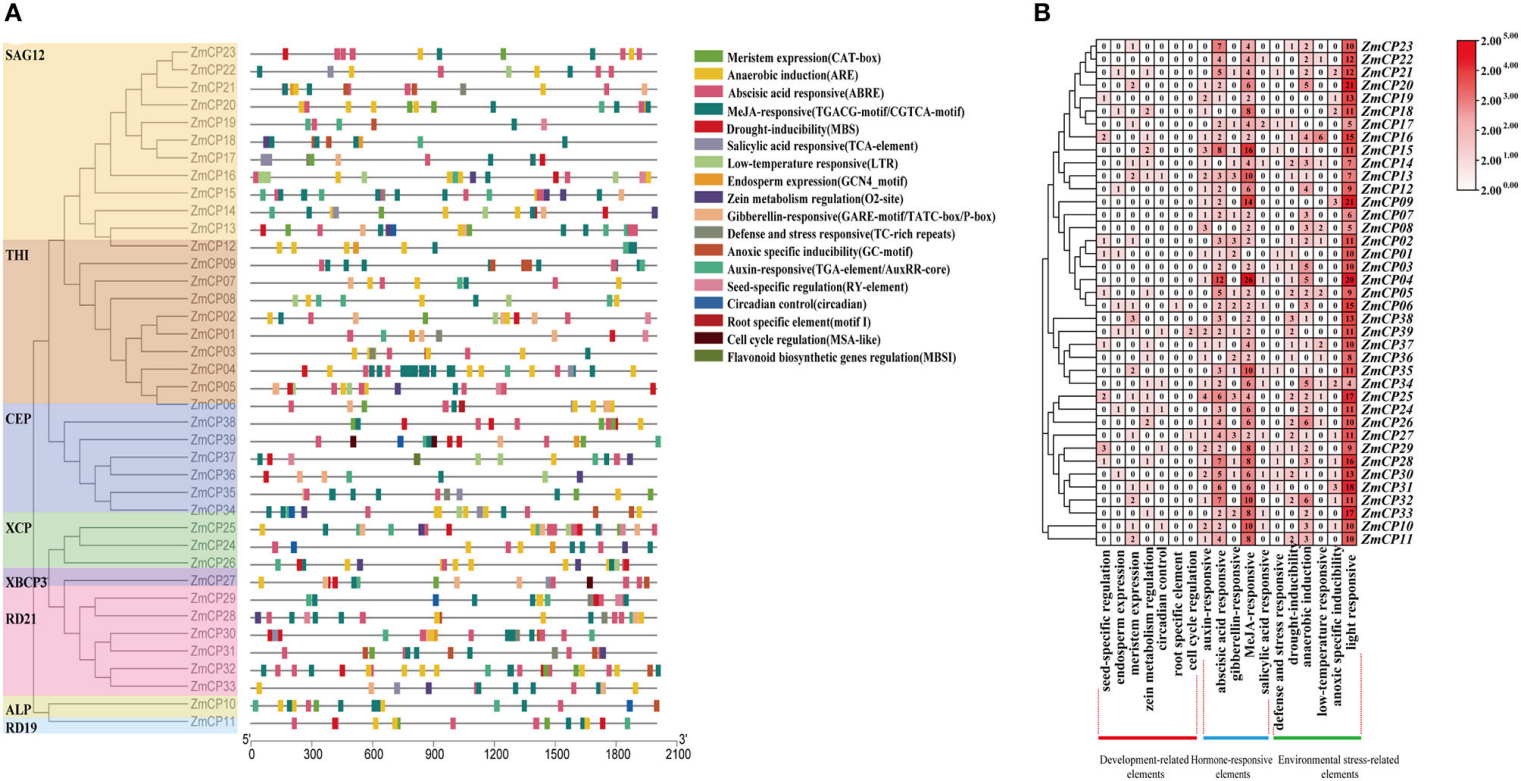
Promoter *cis-*elements analysis of *ZmCP* genes. **(A)** The position of elements in ZmCP promoters. **(B)** The number of occurrences cis-elements in the promoter region of ZmCP genes.

The upstream region of a gene, containing the promoter and the binding site of transcription factors, regulates the gene expression. Thus, a *cis*-element analysis helps to understand gene regulation and function (Higo et al., 1998, 1999; Qanmber et al., 2019a). To identify the putative *cis*-acting regulatory DNA elements, the 2,000-bp upstream sequences from the translation start codons of *ZmCP* genes were retrieved and analyzed in the PlantCARE database (Lescot et al., 2002). As a result, the promoters of *ZmCP* genes contained most elements or sites responsive to environmental factors (e.g., light, temperature, and moisture). The existence of defense and stress responsive elements and MYB binding sites suggested that *ZmCPs* genes were involved in drought and anaerobic responsive elements and may play an important role in counteracting abiotic stress (Figure 3). The hormone-responsive elements, ~40% of total elements, including abscisic acid (ABA), salicylic acid, gibberellin, auxin, and methyl jasmonate (MeJA) -responsive elements, were found within the *ZmCP* gene promoters.

Moreover, 73 elements related to plant growth and development, namely, 23 meristem expression elements, 13 seed-specific regulation elements, 8 endosperm expression elements, 6 circadian control elements, 19 zein metabolism regulation elements, 3 cell cycle regulation elements, and 1 root specific element, were identified within the *ZmCP* gene promoters, indicating that some *ZmCP* genes may regulate plant growth and development.

### Chromosomal Location, Collinearity, and Gene Duplication of *ZmCP* Genes

**Figure 4:**
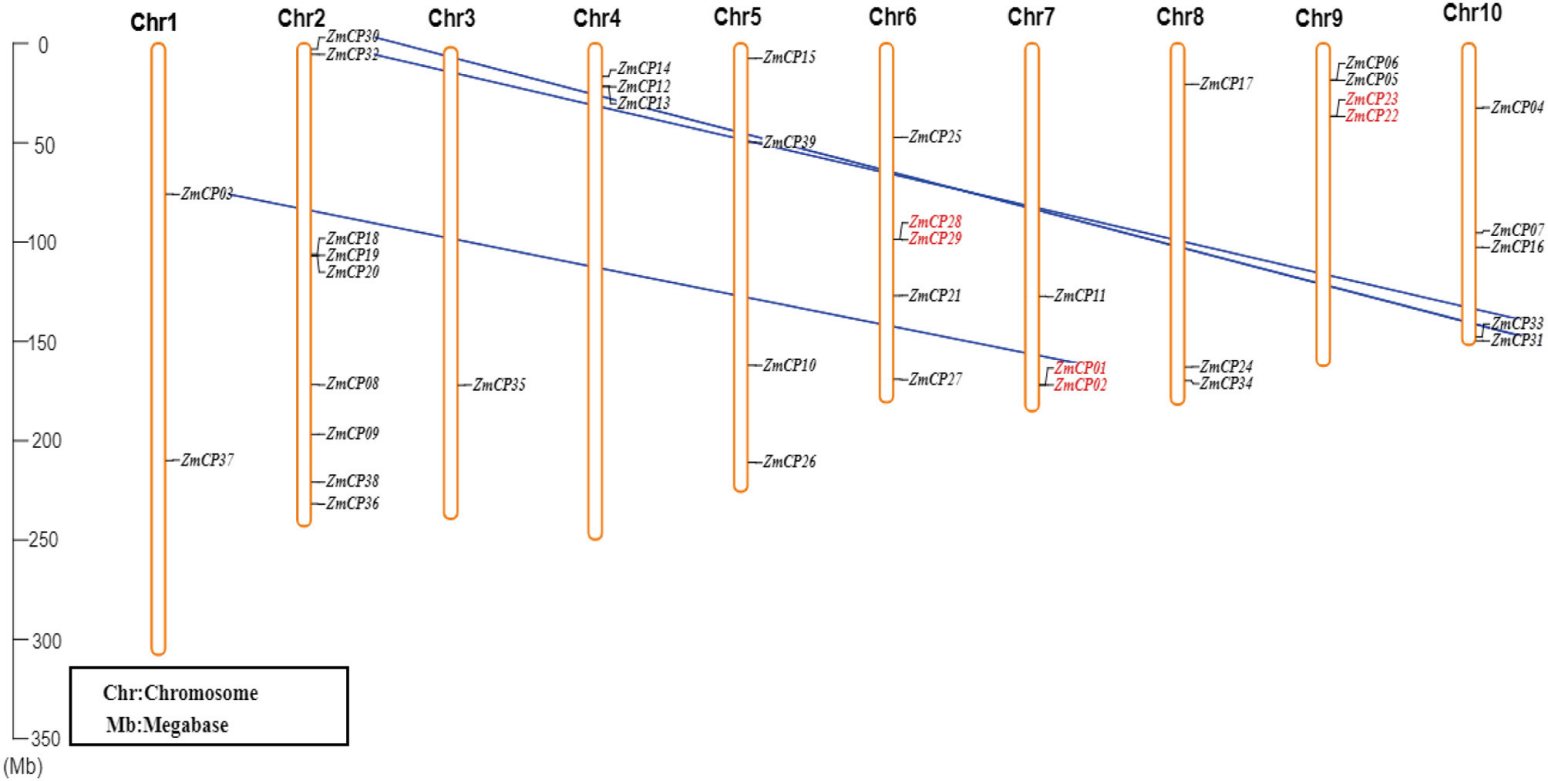
The chromosomal distribution of *ZmCP* genes. The left scale represents the length of the chromosomes. The chromosomal segmental duplications between two *ZmCP* genes are displayed by blue lines, and the two *ZmCP* genes that occurred as tandem duplication events are indicated in red.

The locations of *ZmCP* genes were mapped according to the data of gene locus, and they were unevenly distributed on chromosomes (Figure 4). Specifically, chromosome 2 contained more *ZmCP* genes (9), chromosomes 4, 7, and 8 each contained three, and chromosomes 1, 5, 6, 9, and 10 contained two, four, five, four, and five, respectively. Only *ZmCP35* was localized on chromosome 3. The target gene *ZmCP03* was located on chromosome 1. These indicated that genetic variations existed during the evolutionary process of maize. Similarly, in rice, 33 *OsCPs* were located on 10 chromosomes of 12 chromosomes in an unbalanced pattern (Wang et al., 2018). In cotton, *GhCP* genes were unequally distributed on 21 chromosomes (Zhang et al., 2019). Obviously, the number of *CP* genes differed, and their chromosomal distribution was unequal in the examined plant species.

Duplication is a major driving force for gene expansion during evolution. There are five types of gene duplication in evolution, which are singleton, dispersed, tandem, proximal, and segmental duplication (Zheng et al., 2020). The collinearity of *ZmCP* genes was examined in maize (Figure 4). As a result, three pairs (*ZmCP01*/*ZmCP02, ZmCP22*/*ZmCP23*, and *ZmCP28*/*ZmCP29*) from *ZmCP* genes were discovered in tandem repeats. Moreover, the three pairs (*ZmCP01/ZmCP03, ZmCP30/ZmCP31*, and *ZmCP32/ZmCP33*) probably resulted from the duplicated chromosomal segments, suggesting that *ZmCPs* were expanded by tandem and segmental duplication.

There are three types of the preservation of duplicated genes functional divergence during long-term evolution that are neo-functionalization (gaining new functions), sub-functionalization (partition of actual functions), or non-functionalization (loss of actual functions) (Prince and Pickett, 2002). The *Ka* and *Ks* were calculated for six gene pairs to explore whether Darwinian positive selection influenced the divergence of *ZmCP* members after the duplication. The *Ka*/*Ks* ratio indicates the gene divergence under selection pressure (Hurst, 2002). In maize, all duplicated gene pairs with *Ka/Ks* < 1 indicated negative selection (Table 2).

**Table 2:** Non-synonymous (*Ka)*/synonymous (*Ks)* analysis of *ZmCPs* in maize.

**Duplicated pair**	**Duplicate type**	***Ka***	***Ks***	***Ka/Ks***	**Positive selection**
*ZmCP01/ZmCP02*	Tandenm	0.033075715	0.066598	0.496645	No
*ZmCP22/ZmCP23*	Tandenm	0.012837284	0.16599	0.077338	No
*ZmCP28/ZmCP29*	Tandenm	0.304310686	0.488655	0.622752	No
*ZmCP01/ZmCP03*	Segmental	0.442911131	0.638932	0.693206	No
*ZmCP30/ZmCP31*	Segmental	0.042436719	0.36131	0.117453	No
*ZmCP32/ZmCP33*	Segmental	0.038677537	0.21077	0.183506	No

*Ka/Ks < 1 means negative selection, Ka/Ks = 1 means neutral selection, and Ka/Ks > 1 means positive selection*.

### Gene Expression Profile of the *ZmCP* Genes in Different Tissues

**Figure 5:**
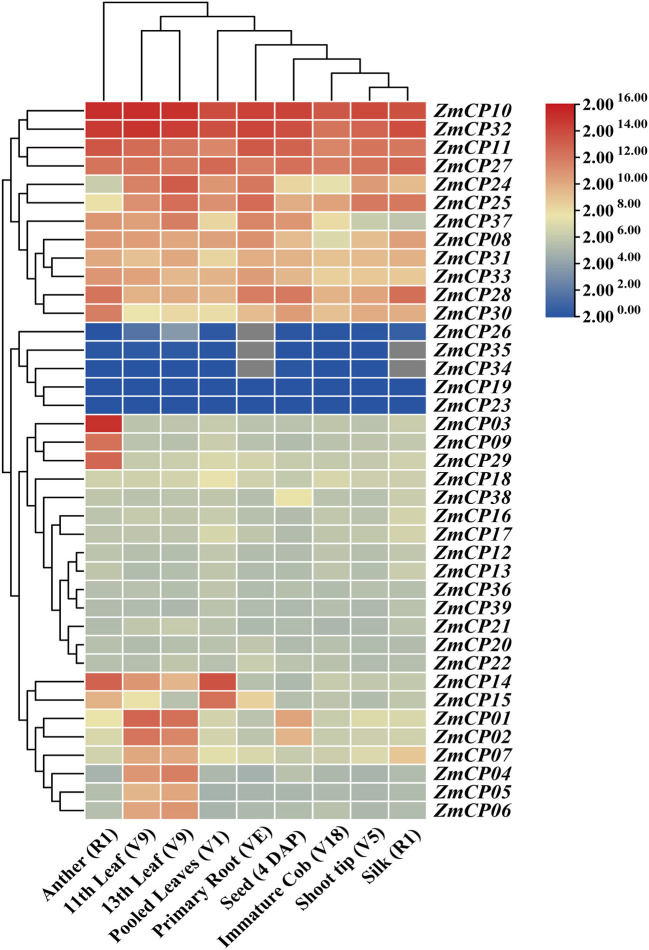
Heatmap of *ZmCP* genes expression levels in maize. R1: silking stage; VE: vegetative emergence; V1, vegetative 1st leaf stage; V5, vegetative 5th leaf stage; V9, vegetative 9th leaf stage; V18, vegetative 18th leaf stage; DAP, days after pollination.

To analyze the expression patterns of the *ZmCPs*, the heatmap of their expression patterns in different maize tissues, including the shoot tip of the vegetative 5th leaf stage (V5), anther at silking stage (R1), primary root at vegetative emergence (VE) and vegetative first leaf stage (V1), pooled leaves from vegetative first leaf stage (V1), 11th leaf and 13th leaf at vegetative 9th leaf stage (V9), immature cob at vegetative 18th leaf stage (V18), silk at silking stage (R1), and seed from 4 DAP (days after pollination) (Figure 5), were built. The transcriptome data retrieved from MaizeGDB indicated the differential expression patterns of *ZmCP* genes during maize growth and development.

Particularly, *ZmCP10, ZmCP11, ZmCP27*, and *ZmCP32* displayed high expression levels in all examined tissues. *ZmCP11* and *ZmCP27* had relatively high expression levels in these tissues, especially *ZmCP11* in anthers, primary roots (VE and V1), and *ZmCP27* in leaves (V1). *ZmCP14* and *ZmCP15* represented relatively high expression levels in young leaves but low expression levels in elder leaves. However, *ZmCP01, ZmCP02*, and *ZmCP04-07* were highly expressed in old leaves but lowly in young leaves. *ZmCP34* and *ZmCP35* had no expressional information in the primary root and silks and showed very low expression levels in other tissues. *ZmCP26* had no expressional information in the primary root and other organs.

*ZmCP03*, the gene of interest, showed a tissue-specific expression pattern with unusually high expression levels in anthers but low expression levels in other tissues, suggesting it may be related with anther development. All members of the subfamily RD21 had high expression levels in anthers, as was the case in cotton (Zhang et al., 2019).

**Figure 6:**
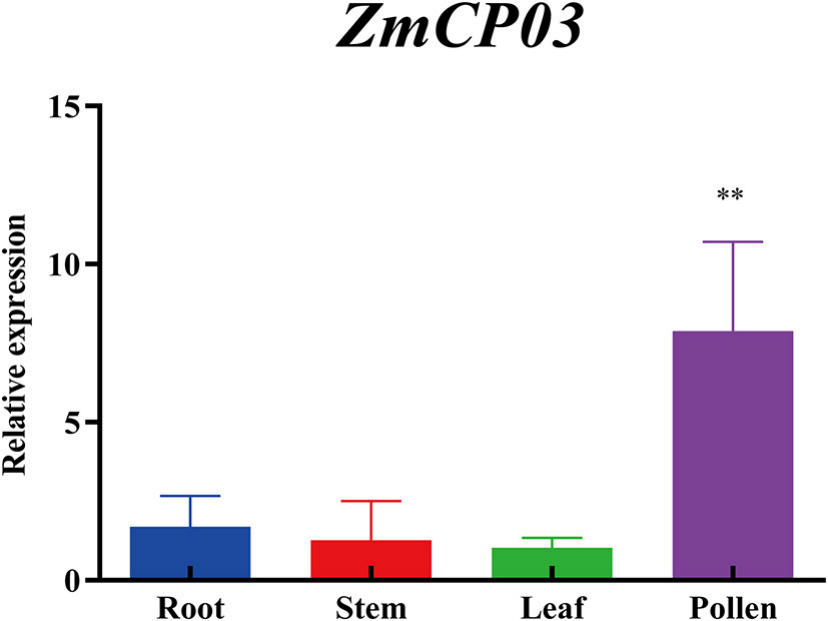
Quantitative reverse transcription PCR (RT-qPCR) analysis of *ZmCP03* in different organs of maize. Data presented are relative expression (mean ± SD) from the three biological replicates after normalization based on *ZmUBI* expression levels. Student's *t*-test in the GraphPad Prism 8.0 software was used to compare significant differences (indicated by ** at *p* < 0.01) in expression levels among different tissues.

As a comparison with the transcriptome data retrieved from MaizeGDB, the expression levels of *ZmCP03* in the root, stem, leaves, and pollen of maize were further analyzed using RT-qPCR. *ZmCP03* had a high expression level in pollen grains but a low level in other tissues, consistent with the results of the transcriptome data (Figure 6).

**Figure 7:**
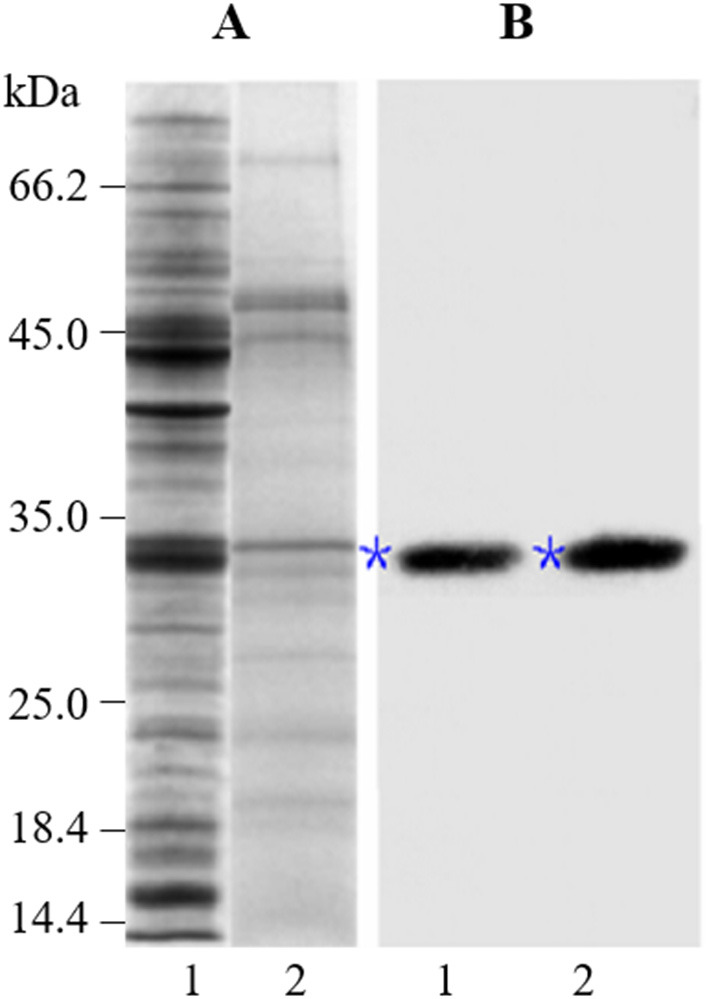
Detection of ZmCP03 in pollen grains of maize. Proteins were separated on an equal amount of pollen fresh weight. **(A)** Sodium dodecyl sulfate polyacrylamide gel electrophoresis (SDS-PAGE), 12.5% resolving gel; **(B)** Immunoblotting, detection with anti-ZmCP03 (1: 5000 dilution). Lanes 1, pollen grains; 2, pollen coats. Asterisks indicate ZmCP03.

**Figure 8:**
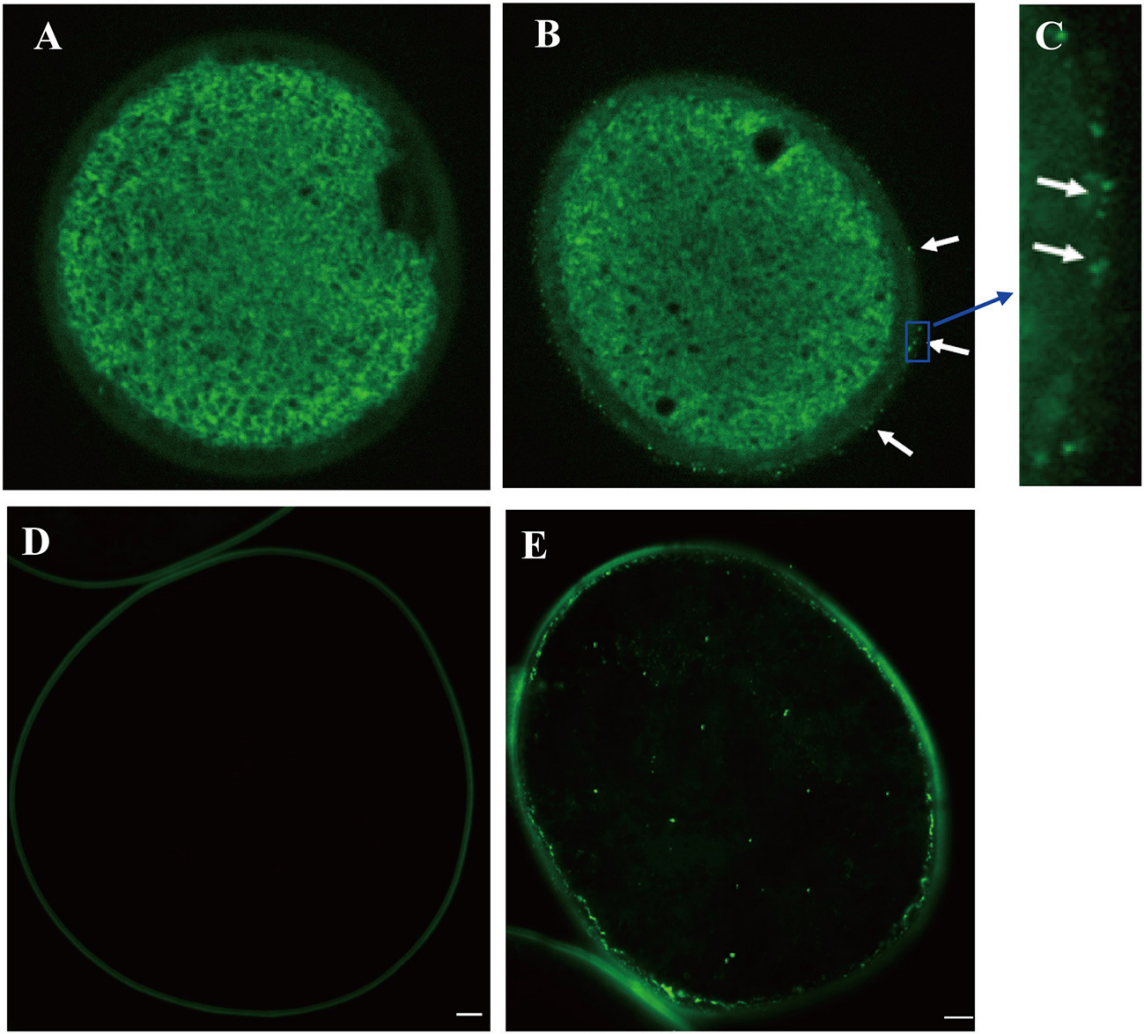
Localization of ZmCP03 in maize pollen by immunofluorescence microscopy. **(A)** Control without primary antibody, no signal is detected; **(B)** in the presence of primary antibody, several fluorescent dots can be seen on the surface of pollen grains (arrows); **(C)** a magnification picture of **(B)** highlighting some dots on the grain surface; **(D)** sections of maize pollen without a primary antibody; **(E)** sections of maize pollen with a primary antibody. Maize pollen sections were probed with **(B,D)** anti-CP (1:200 dilution, 4°C, overnight) or **(A,D)** negative control, and then incubated with fluorescence anti-rabbit lgG (1:100, 37°C, 45 min). Scale bar = 5 μm.

To examine the accumulation and localization of ZmCP03 in pollen grains, a monoclonal antibody (anti-ZmCP03) was prepared using a synthetic 15 peptide to immunize mice. Sodium dodecyl sulfate polyacrylamide gel electrophoresis and immunoblot analyses revealed the abundant accumulation of ZmCP03 in pollen grains, especially in the pollen coat component (Figure 7). In addition, fluorescence microscopy showed distinct bright spots detected on the pollen surface of intact pollen grains (Figures 8A–C). The presence of ZmCP03 was further examined by fluorescence microscopy using semi-thin sections of pollen grains. Obviously, ZmCP03 signals were most present on the pollen surface (coat) and in small quantities inside pollen grains (the cytoplasm) (Figures 8D,E).

**Figure 9:**
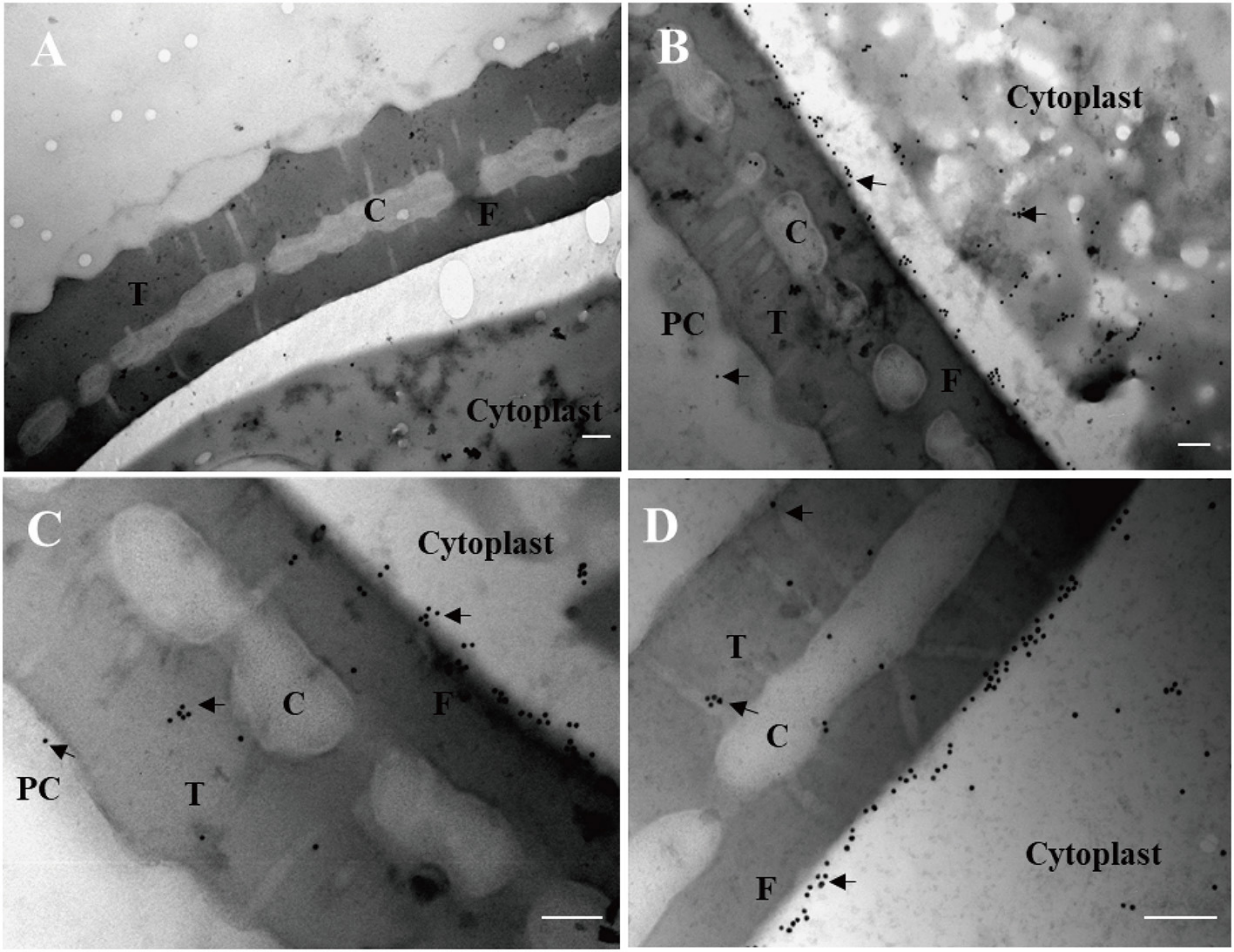
Subcellular localization of ZmCP03 in maize pollen by immunogold electron microscopy. **(A)** Negative control, preimmunized rabbit serum, and then incubated with 15-nm gold-conjugated anti-rabbit lgG (1:20, 45 min); **(B**–**D)** sections of maize pollen grains were probed with anti-CP (1:100 dilution, 4°C, overnight); arrows indicate the presence of ZmCP03; PC, pollen coat; T, tectum layer, C, columella; F, foot layer; The exine consists of the tectum layer, foot layer, and columella. Scale bar = 200 nm.

Finally, immunogold electron microscopy was carried out to examine the subcellular localization of maize pollen ZmCP03. Ultrathin sections of maize pollen grains were probed with the anti-ZmCP03. The results clearly demonstrated that ZmCP03 was located on the pollen surface (coat), but particularly in the inner layer of the wall (arrows). Furthermore, ZmCP03 signaling was also in the pollen cytoplasm (Figures 9B–D).

## Discussion

Several reports investigated the evolutionary relationship of papain-like cysteine proteases (PLCP) family in Arabidopsis (Richau et al., 2012), rubber (Zou et al., 2017), papaya (Liu et al., 2018), rice (Wang et al., 2018), castor bean and physic nut (Zou et al., 2018), and cotton (Zhang et al., 2019), and soybean (Yuan et al., 2020). In maize, a report analyzed the evolutionary relationship of *CP* genes only through building phylogenetic tree (Richau et al., 2012). In the present study, 39 *CP* genes in maize were analyzed using several bioinformatic tools, especially comparing *CP* in pollen and other tissues, to provide insights on the potential function of ZmCP03 in pollen development and germination.

The results here showed that the number of subfamilies of papain-like cysteine protease differed greatly in maize. SAG12 (12 members) and THI (9 members) were the two largest subfamilies, like in rice (Wang et al., 2018). However, the two subfamilies owned much more members in *Ficus carica* (Zhai et al., 2021) and Arabidopsis (Richau et al., 2012), implying that SAG12 may be generated earlier than the formation of individual species. Usually, SAG12 is significantly increased during leaf senescence, thus it is most widely used as a senescence-associated reference gene (James et al., 2018). The CTB subfamily has a negative role in regulating cryo-injury tolerance and cell viability *via* mediating the PCD event in plant cryopreservation in Arabidopsis and *Agapanthus praecox* (Chen et al., 2021). However, no CTB subfamily was identified in maize in the present study, perhaps being lost during evolution.

The results here further showed that the family of papain-like cysteine protease is relatively conservative during the evolution events based on their motif, conserved domain, and gene structure (Figure 2). All 39 ZmCP proteins are composed of the peptidase C1 domain and inhibitor I29 domain. In the same subfamily, most members have similar exon-intron structures, suggesting affinity and the conservation of evolutionary relationships. Meanwhile, segmental duplications and tandem duplications may have the same effect on expanding this family in maize. *Ka/Ks* < 1 indicates that duplicated gene pairs in *ZmCPs* were all purified selection, according to the theory that a species is more likely keeping protein as it is (purified selection) rather than a mutation occurring that changes the protein (Hurst, 2002). Moreover, *ZmCP* genes maintain conserved and stable evolutionary selection, suggesting that their functions were stabilized in maize. However, the *CP* genes in rice displayed different evolutionary selection ways, especially five out of seven pairs of tandem duplications were positive selection, and segmental duplications belong to purified selection in rice (Wang et al., 2018).

Previously, ZmCP03 was found to be synthesized in the rough endoplasmic reticulum (ER) and processed during or after its transfer to the vacuole at stage 4 of anther development (Li et al., 2012). In Arabidopsis, *AtTHI1* (*AtCP51*) participates in pollen exine formation and anther development, and the pollen of transgenic CP51-RNAi plants was aborted due to defective exine and early degrading tapetum (Yang et al., 2014). According to the phylogenetic trees here, *ZmCP03* was in the same clade with *OsCP12* and in the same group with *GhTHI1, GhTHI2*, and *AtTHI1*. As *OsCP12* displayed an organ-special expression pattern with a high expression level in anthers (Wang et al., 2018). *ZmCP03* was also highly expressed in anthers but low in other tissues (Figure 6). Moreover, it was verified that ZmCP03 was mainly found in the pollen coat and, to a lesser extent, in the cytoplasm (Figures 8, 9). Therefore, both the previous studies (Li et al., 2012) and the present results supported the speculation that ZmCP03 may be related to the PCD of the tapetum and play a role in pollen development and germination.

In addition, the upstream region of the *ZmCP03* contained the MYB binding site involved in drought-inducibility and the abscisic acid (ABA) -responsive element (ABRE), which is recognized as a necessary element controlling ABA-regulated gene expression (Fujita et al., 2005). A previous report indicated that a C1 cysteine protease was highly induced by drought in soybean (Cilliers et al., 2017), suggesting that *ZmCP03* may be involved in the response to drought stress.

In conclusion, we characterized in this study the *CP* genes family in maize at a genome scale using comprehensive bioinformatic tools and further compared with Arabidopsis, cotton, and rice. Particularly, maize *CPs* in pollen coat and other tissues were emphatically compared. The evolutionary relationship, gene structure and duplication, chromosomal distribution, sequence characteristics, and expression profiles of *ZmCP* genes suggest that *ZmCP03* is relatively conservative and stable in the process of evolution. This study may provide referential evidence on the function of ZmCP03 in maize pollen development and germination.

## Data Availability Statement

The original contributions presented in the study are included in the article/Supplementary Material, further inquiries can be directed to the corresponding author/s.

## Author Contributions

WW and GC conceived the research. YL, LN, CF, GC, and MZ performed the experiments. XW, FT, WW, and GC analyzed the data. YL, HL, and WW drafted the manuscript. LN, GC, and WW edited the manuscript. All authors contributed to the article and approved the submitted version.

## Funding

This study was supported by the National Natural Science Foundation of China (Grant 31771700).

## Conflict of Interest

The authors declare that the research was conducted in the absence of any commercial or financial relationships that could be construed as a potential conflict of interest.

## Publisher's Note

All claims expressed in this article are solely those of the authors and do not necessarily represent those of their affiliated organizations, or those of the publisher, the editors and the reviewers. Any product that may be evaluated in this article, or claim that may be made by its manufacturer, is not guaranteed or endorsed by the publisher.

## Supplementary Material

Supplementary Figure 1 The unrooted phylogenetic tree was generated based on the amino acid sequences of 39 ZmCPs using a maximum likelihood method with 1,000 bootstrap values.

Supplementary Dataset 1 The nucleotide and amino acid sequences of 39 ZmCPs.
