# Supplementary figures and images for: Genome-Wide Identification and Comparison of Cysteine Proteases in the Pollen Coat and Other Tissues in Maize
Source: Front Plant Sci. 2021 Sep 23;12:709534. doi: 10.3389/fpls.2021.709534 (PMC8494779; doi:10.3389/fpls.2021.709534)

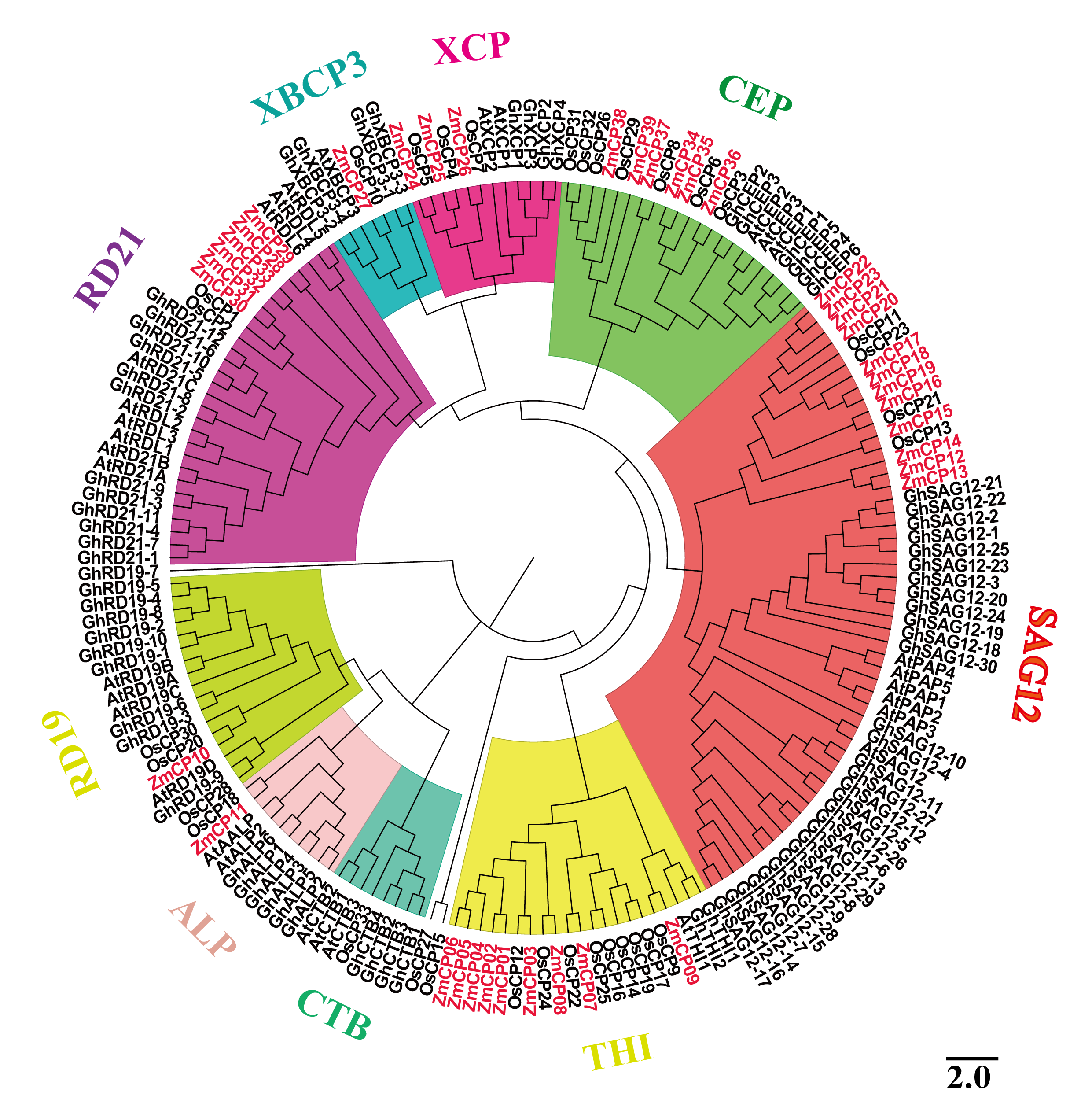

Supplement: Supplementary Figure 1 — The unrooted phylogenetic tree was generated based on the amino acid sequences of 39 ZmCPs using a maximum likelihood method with 1,000 bootstrap values. [file Image_2.TIF]

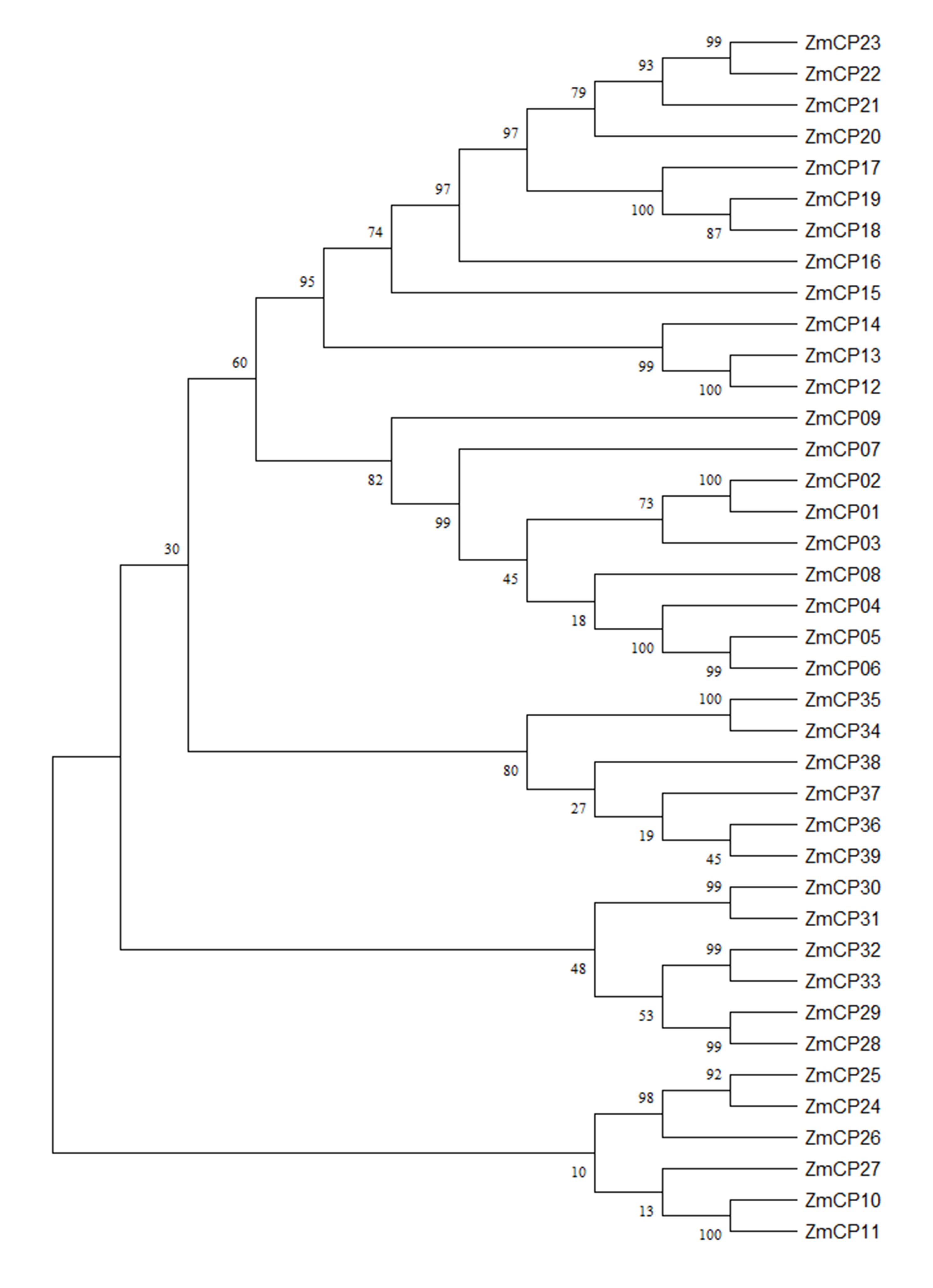

Supplement: Supplementary file 3 [file Image_1.TIF]
