# Supplementary material for: Genome-Wide Identification and Comparison of Cysteine Proteases in the Pollen Coat and Other Tissues in Maize
Source: Front Plant Sci. 2021 Sep 23;12:709534. doi: 10.3389/fpls.2021.709534 (PMC8494779; doi:10.3389/fpls.2021.709534)
Supplement: Supplementary Dataset 1 — The nucleotide and amino acid sequences of 39 ZmCPs. [file Data_Sheet_1.docx]

**Nucleotide sequences：**

>ZmCP01

ACTCAGATTCAGTACAAGCTCAAGCCAGGCACACAGCAAGACATCGACCATGGCTGCCTACCAACAAGCTCCCGCTCTCCTGTGCGCCTGCCTGATGCTCGTGCTCATGGCGGGCGCGGCATCCGGCGGCCGCGTAGACGTGGAAGACATGCTGATGATGGACCGGTTCCGCGCGTGGCAAGCCACGTACAACCGCTCGTACCTGACCGCCGCGGAGCGTCTGCGCCGGTTCGAGGTGTACCGCCAGAACATGGAGCTCATCGAGGCCACGAACCGCCGCGCCGAGCTCTCGTACCAGCTCAGCGAGACCCCGTTCACCGACCTCACCAGCGAGGAGTTCCTCGCCACACACACCATGTCCACGCGCCTGCATGCGTCCGAGGCCGCGCGGCGGCACCGGGAGCTCATCACGACGCACGCAGGCCCCGTCAGCGACGGCGGTCGCCAGTGGAACCGGCGGAACTACACGACGGACCTCGACGTCCCCGAAAGCGTGGACTGGAGGACCAAGGGCGCGGTGACGACGGTCAAGGACCAAGGAGCTTGTGGTAAACGATAGCATTTACATACTTATTCCTTGCTAGCTATATATGAAAAGAAGTTAAAATATAGACACACACCATAATAGATGATCGAAGATGCTGCCCCTCCTTGCAGGCGGTTGTTGGTCATTCGCGACGGTGGCGGCGATCGAAGGCCTGCACAAGATAAGGACGGGCCAGCTTGTCTCCCTGTCGGAGCAGGAGGTTCTGGACTGCTCTTCCCCGCCCAACAATGGCTGCCACGGCGGCAACCCCGCGGCTGCCATAGACTGGGTGTCCGCTAACGGCGGCCTCACCACCGAGTCGGACTACCCGTACGAGGGGCGGCAGGGCAAATGCAAGCTCGACAAGGCACGCAACCACGTCGCCAAGATCAGAGGCAGGAAGTTGGTGGATCAAAACAACGAAGCCGCGCTGGAGGTCGCCGTGGCGCAACAGCCTGTTGCCGTGGGCATGAATGTACATCCGATCCAACAGCACTACAAGAGCGGGGTGTTCCATGGCCCCTGCGACCCAGAGGATCTCAACCACGCCGTCACCATGGTGGGGTATGGTGCCGAATCCGGTGGCCGCAAATACTGGATCGTCAAGAACTCGTGGGGCGAGAAGTGGGGCGAGAAGGGATACTTTCGCGGTTTCGCCTCGAGAGGCGCGTCAAGGACGTCAGGGGCACCTGCGGTATAGCCGAGTGGGCAAACTACCCGGTGATGTGAGCTCCCGTCCCGTGTCTGTCGCCACCGTGCTTTACCTGCCTGGCCTACAGTGCGGTCGTCTTGCTTTCCTGCACATGCGCAAGCCTATATATGATGATGTGCAGAAGTCAGCTTGTAGAACAGGCTGAAATAAACTCGACGCGCCCGGCATGTGTACTCGGCAGTGTCGTCGTTGTCATTTGCCTATTATCTATCATCTTAAGAACTATATAGTAAGCTCCATGTGTAGCTGCTAAATAAAAACGCCCCCTTAAAT

>ZmCP02

AAGGTGCGCATGCATGGCGAGAGATCGCCACACCGTGCCGGCCGCTATATAAACCCGTCGTGTCCATGGCTGCTATATCACTCAGACTCAGTACAAGCTCAAGCCAGGCACACAGCAAGACATCGACCATGGCTGCCTACCAACAAGCTCCCGCTCTCCTGTGCGCCTGCCTGATGCTCGTGCTCATGGCGGGCGCGGCATCCGGCGGCCGCGTAGACGTGGAAGACATGCTGATGATGGACCGGTTCCGCGGGTGGCAAGCCACGTACAACCGCTCGTACCTGACCGCCGCGGAGCGTCTGCGCCGGTTCGAGGTGTACCGCCAGAACATGGAGCTCATCGAGGCCACGAACCGGCGCGCCGGGCTCTCGTACCAGCTCGGCGAGACCCCGTTCACGGACCTCACCAGCGAGGAGTTCCTCGCCACACACACCATGTCCACGCGCCTGCATGCGTCCGAGGCCGCGCGGCGGCACCGGGAGCTCATCACGACGCACGCAGGCCCCGTCAGCGACGGCGGTCGCCAGTGGAACCGGAACTACACGACGGACCTCGACGTCCCCGAAAGCGTGGACTGGAGGACCAAGGGCGCGGTGACGCCGGTCAAGGACCAAGGAGCTTGTGGTAAACAATAGCATTATTTACCCATCATATCACACACACTTATTCCTACGACGACGACGACGACGATAACGATCCTTGACGCTGTCCATCTTTGCAGGCAGTTGCTGGTCATTCGTGACGGTGGCGGCAATCGAAGGCCTGCACAAGATAAGGACGGGCCAGCTCGTCTCCCTGTCGGAGCAGGCGGTTCTGGACTGCTCTTCCCCGCCCAACCATGGCTGCAACCGCGGTGACCCCGCGGCTGCCATAGACTGGGTGTCCGCTAACGGCGGCCTCACCACCGAGTCGGACTACCCGTATGTGGGACGGCAGGGCAAATGCAAGCTCGACAAGGCACGCAACCACGTCGCCAAGATCAAAGGCAGGAAGTTGGTGGATCAAAACAACGAAGCCGCGCTGGAGGTCGCCGTGGCGCAGCAACCTGTTGCCGTGGACATGAATGTAGATCCGATCCTCCAGCACTACAAGAGCGGGGTGTTCCATGGCCCCTGCGACCCAGAGGATCGTCAACCACGGCGTCACCATGGTGGGGTACGGTGTTGAATCTGGCGGCCGCAAGTACTGGATCGCCAAGAACTCGTGGGGCAATGAGTGGGGCGAGAAGGGATACTTTCGCCTCGAGAGGCGCATCAAGGACGCCAGGGGCACGTGTGGTATAGCCGTGTGGGCAAACTACCCGGTGATGTGAGCTCCAGGGCCTGGCCGTATACTAGTACAATACAGTCGACTTGCTCCTGGACATACGCAGTGCAAGCCTATCTATATATATGATGAGGTGCAGAAGTCAGCTTGAAAAACAGGCTGAAATAAACTCTGACGCGCGCGGCCTGTACTCAGCAAAGCGTCGTCGTCGCCATTTGCTAATTTTATGTGTAATCTTTAAAACTACTATGCTGTGACCTCCATAAGTCGCGGCTAATAAATAGAAATGTTCCCTTAAATAATTTCA

>ZmCP03

ATGGCTCTCTTCCGTGCCGCCGCCTCAGGAGGGTTCGCGCTGATCCTTCTCGCGTGCTGCAGCCTCATAATGCTGGCGGCGGCGTCTGGCGGCGGCGGCGTGGACGACGACGGCGTCGGTGGTGACAGGCTGATGATGGACCGGTTCCTCAGCTGGCAGGCGACGTACAACCGGTCGTACCCGACGGCGGAGGAGAGGCAGCGCCGGTTCCAGGTGTACCGCCGCAACATTGAGCACATCGAGGCCACCAACCGGGCGGGCAACCTGACGTACACGCTCGGCGAGAACCAGTTCGCCGACCTCACGGAGGAGGAGTTCCTGGACCTGTACACCATGAAGGGGATGCCCGTCCGCCGTGACGCCGGCAAGAAGCGGGCCAACGTATCCTCCTCCGCCGCTGCCGTGGACGCTCCGACCAGCGTCGACTGGAGGTCCAAGGGCGCCGTCACGCCGATCAAGAACCAGGGCCCGTCGTGCTGTCCGTATATCTATATATGTTCTCCTTTGATTTTGCACCTATGCACCTCTGCGCGCAATCCGTTAATTGATTTCTGCATGCAGCTAGCTGCTGGGCATTCGTGACGGCGGCGACGATCGAGAGCATCACCAAGATCACGACAGGGAAGCTGGTGTCGCTGTCGGAGCAGGAGCTGATCGACTGCGACCCCTACGACGGCGGCTGCAACCTGGGCTACTTCGTGAACGGCTACAGGTGGGTGATCCAGAACGGCGGGCTCACCACCGAGGCCAACTACCCGTACCAGGCGCGGCGGTACGCGTGCAGCCGCTCCAGGGCCGCCCAGCACGCCGCCACCATCTCCGACTACGTGCAGCTGCCGGCGGGCGAGGGCCAGCTGCAGCAGGCGGTGGCGCAGCAGCCCGTGGCCGCGGCCATCGAGATGGGCGGCAGCCTGCAGTTCTACAGCGGCGGCGTCTTCTCGGGGCAGTGCGGGACCAGGATGAACCACGCCATCACCGTCGTCGGCTACGGCGCCGACTCCTCCTCGGGCCTCAAGTACTGGCTCGTCAAGAACTCGTGGGGGCAGAGCTGGGGCGAGCGCGGATACCTGCGGATGCGCCGCGACGTCGGGCGCGGGGGACTGTGCGGCATCGCGCTCGACCTCGCGTACCCGGTCGTGTGA

>ZmCP04

AAGGCAGCAAGGCCACTAACACTAGTTATCACATCCACCATAACGACCATGGCTACCACCTCAGCTTTGCTAGCTCTTGTCTTGTTAGCCAGCCTCCTTGCAGGCACGGTGTTCAGTGATGATATCGTACCCATCCATATACCCTTGTTGGATCGGTTCCAAGCCTGGCAGGCCGAATACAACCGCACATATGCGACCCCAGAGGAATTCCAGCAACGCTTCATGGTCTATAGCGAGAATGTCAAGTTCATCGAGACCATGAACCAGCCTGGGAGCTCATATGAGCTCGGTGAGAACCAATTCGCTGACCTCACCGAGGAGGAGTTCAAGGACACGTATCTTATGAAGCTTGACAACGTGGCCTCGTCCCCTGAGGCCATGGCACTGACCGTCGATACCATGAATAGGCAGGCACATCTGGCGGCAGCAACACCAACGAGGCTCCCAATAGTGTGGACTGGAGGACCAAGGGAGCAGTGACGCCGGTCAAGAGCCAGCAACATTGTGGTAGGCGAAAACATTAATGACTACACCCTCTATTTATCTATCCATTTCAAGTTTATGTTAAAAAATAATAAATATTGACTAAATTATTTGTTAAATATCATAAAGTTTTTGTCTTTTGGGTCTTGATGTGTGTGTCTGCCATAGAAATAAAAACAGACAGAACACTGCTTTATCCAATTATAGTATGTCAGTCATTGTTTTTGTGTGGAAACAAAGACTCATGTCATGCATGTACATGCTCCATGCCTGACAAGATAGTCATCCTTGCATGCAGGCTCTTGCTGGGCGTTTGCCGCAGTGGCGTCTATCGAAGGTGTGCACAAGATAAAGACAGGTCGTCTCGTGTCACTGTCGGAGCAAGAGATTGTGGATTGCGACCGTGGGGGGAATAACCATGGCTGCCATGGTGGTCACTCTAGTTCTGCCATGGAGTGGGTCACCCGCAACGGCGGCCTGACCACCGAGTCGGACTACCCATATGTGGGTAGGCAGGGACAATGCATGAGCGACAAGCTTGGTCATCATGCGGCCAAGATCCGTGGCCGGCAGGCAGTGCAGGGCAAGAACGAGGGCGCGCTACAGCATGCAGTGGCGGGGCGGCCGGTGGCCGTAAGCATCAATGCAAGCAGGGCCTTCCAGTTCTACAAGCGCGGGATTTTCTCAGGCCCCTGCAACACCACCCGGAACCACGCCGTCACGGTGGTCGGGTATGGAGCCAACGCTAGTGGCCACAAGTACTGGATTGTCAAGAACTCATGGGGTGAGAGGTGGGGAGAGAAGGGCTACGTGCGAATGCAGAGGGGTGTTAGGGCTAGGGAGGGCGTGTGCGGCATCGCCATAGCGCCCTTCTATGCGGTGATGTGAGAGTACCGTCTCTCCCCATACTAGTTACCAGTTGCATTCTGATTCTAAACAAGTATGGGTGTCATCTTGGAGAAGAATGCATGGAATTAAATAAACTACAGCTCACCGAGGGATGGTACATGATGTTTGCTACGCGTTGCCAATTTATCGTTTGTTTGCTAATGTAACAAAATCTCAAAATCAATGACTTTTGGGATGTAATATGTGTTCTTGTTAAATAAAGAGAATCACAATTGATCTCTTGCCTTAAAACATCATCACTGCGATGTGCAAGAGGTTAGTTTGTCAA

>ZmCP05

CAGCAGTTGCACTAGTCATCGCATCCACCATAACATGACCATGGCTACCGCCTCAGCTTCGCTAGCTCTTGTCATGCTGTTCGCCTGCAGCCTTCTCCTTGCAGGCACGGCGTTCAGTGATGATACCATAGCCATACCCTTGCTGGAGCGGTTCAAAGCCTGGCAGGCTGAATACAACCGCACCTATGCGACCCCAGAGGAATTCCAGCAACGCTTCATGGTCTACAGCGAGAACCTCCGGTTCATCAAGACCATGAACCAGCTGTCGACCGGGAGCTCGTATGAGCTCGGTGAGAACCAATTCACCGACCTCACCGAGGAGGAGTTCAAGGACACATATCTTATGAAGCTCGACGAGCAGCCCCCTGCAGCTGAGGCCATGCCACCGATCGTCGGCACCATGAGCACAGCAGGCATGTCCAACGGCGACAACACCGGCGAGGCTCCCAACAGTGTGGACTGGAGGACCAAGGGAGCAGTGACGCCGGTCAAGAACCAGCAACAATGCGGTAAAGTTTGGGTCTTGATGTTTGTTTCTGTCTTAGAAATGAAAAAAACGAAGGGAATGTCATGCATCTATTTATGACCCATGCATATGTAAAAGATTTGCTCATCATCCTTGCATGCGTGCAGGCTCTTGCTGGGCGTTTGCAACGGTGGCGTCCATCGAAGGTGTGCACCAGATAAAGACAGGTCGTCTGGTGTCCCTGTCGGAGCAAGAGATCGTGGACTGCGACCGTGGGGGGAACGACCACGGCTGCCGTGGCGGTTACCCTAGATCCGCCATGGAGTGGGTCACCCGCAACGGCGGCCTGACCACCGAGTCGGACTACCCGTACGTGGGCAGCCAGAGACAATGCATGAGCGGCAAGCTTGGTCACCACGCGGCCAGGATCCGTGGCTACCAGGCGGTGCAGCGCAAAAACGAGGCCGAGCTGGAGCGCGCCGTGGCGGGACGCCCGGTCGCCGTGGTCATCGACGCGAGCAGGGCCTTCCAGTTCTACAAGCGCGGGGTCTTCTCGGGCCCCTGCAACACCACCACCGTCAACCACGCCGTCACGGTGGTCGGGTACGGATCAGCCGGCAGCGACAGCGGCGGCGGCCGCAAGTACTGGATCGTCAAGAACTCGTGGGGTCAGAGATGGGGAGAGAATGGCTACGTGCGGATGGCGAGGCGTGTCAGGGCTCGGGAGGGCATGTGCGCCATCGCCATAGAGCCCTACTACCCGGTGATGTGATCTAAACAAGCTAGTATGCTTGGATAAGGATGCATGCATGGAATTAAATAAACTAAGCTCAACGAGACGGTGTGTGTGTGCTGCGCTATGCCTATCGATGTTATTGTTTGTTTGCTAATGTAACATTTATCTCAAAAGGCAATGACTTTTGGGGATGCAGTATGTGTGTTCTTACATTAAATAAAGAGAATCACAATTGATCTC

>ZmCP06

ATGGCCGGCGTCACTCGGCTGGAGAAGGGGCAGGGCGCAGGCTGGGTACGCGGCGTCCTGGGCGTGGAGCATAGAGCAGGAGAGAGAGAAAGAGAAAGGTGGCGGCTTAGGGAACACCAGGGGGCGACGACTGCTAGGAGAGGCGGCTAGGTCAGCAGCCAGGGAGCGGCGGCTGTGACATGGGCCTAATGGGCTTTAGGGTTTAGGTTTTTTTCTTTTTTATTTCTTTTCTAATTCCTTTATAATTTCAAAATATAATTTTAAATAACCATAAAATTCATAACAATTAACCCAAAATTATTTATAAATAAAATAGTTATTTTTGGACTAATATTATTATATATATTATTTAGTTTTTCATTTAAGAAGAAATGATATTCAATATCCAGAATCACTTATGAAAAATCAAAAATCATTCCAAATGCAAATAAATGATAAACACTTAAAAGAAAATTTATTACCATAATTACCTTTAATAACTAAATGCATTATTTTTATCTAATGATTTTTATAGTGTTACAAGTTTTAAGAAAGTATACTAGCCCAATTCATTATTTACTACATTTTTTCATAGTATGTTTTATCAAAAAAATCATATAAAGATGGTTTTATATTTAGAAGTGTCTAGTATACTCACTAACATCTAAGACAGTTCTTATAGTCTAGACGACTCTAATAATATCTTTATTTGAGATGGTTTCATATATAAAAGTGTATAATATATTAACCAGAATCTAAGACAATTCTTACATTCTTAATGACTCAAAAGGTATATTTATTTAAGATGGTTTTCAATAACAATATGTCTTAAATCAATATAAGACATTTCTTACGATATAACTGTCTCAAAATACTTCTTTATTAAAGACATATCTCAACCAAACCGTCTTATCTTACTCATCACTATATGATAAAAGACGCTTCTATAAAATGCACTGATATCCATCTTAAAATGTGCGTCTTAAATAAGCATATCTCTAGTAGTGCTATGCGACCCAGAGGAATTCCAGCAACGCTTCATGGTCTACAGCGAGAACGTCCGGTTCATCAAGACCATGAACCAGCTGTCGACCGGGAGCTCGTATGAGCTCGGTGAGAACCAATTCACCGACCTCACCGAGGAGGAGTTCAAGGACACATATCTTATGAAGCTCGACGAGCAGCCCCCTGCAGCTGAGGCCATGCCACCGACCGTCGGCACCATGAGCACAGCAGGCATGTCCAACGGCGACAACACCGGCGAGGCTCCCAACAGTGTGGACTGGAGGACCAAGGGAGCAGTGACGCCGGTCAAGAACCAGCAACAATGCGGTAAAGTTTGGGTTTTGATGTGTGTTTCTGTCTTAGAAATGATAAAAACGAAGGGAATGTCATGCATCTATTTATGACCCATGCATATGTAAAAGATTTGCTCATCATCCTTGCATGCGTGCAGGCTCTTGCTGGGCGTTTGCAACGGTGGCGTCCATCGAAGGTGTGCACCAGATAAAGACAGGTCGTCTGGTGTCCCTGTCGGAGCAACAGATCGTGGACTGCGACCGTGGGGGGAACGACCACGGCTGCCATGGCGGTTACCCTAGATCCGCCATGGAGTGGGTCACCCGCAACGGCGGCCTGACCACCGAGTCGGACTACCCGTACGTGGGCAGCCAGAGACAATGCATGAGCGGCAAGCTTGGTCACCAAGCGGCTAGGATCCGTGGCTACCAGGCGGTGCAGCGCAAAAACGAGGCCGAGCTGGAGCGCGCCGTGGCGGGACGCCCGGTCGCCGTGGTCATCGACGCGAGCAGGGCCTTCCAGTTCTACAAGCGCGGGGTCTTCTCGGGCCCCTGCAACACCACCACCGTCAACCACGCCGTCACGGTGGTCGGGTATGGATCAACCGGCAGCGACAGCGGCGGCGGCCGCAAGTACTGGATCGTCAAGAACTCGTGGGGTCAGAGATGGGGAGAGAATGGCTACGTGCGGATGGCGAGGCGTGTCAGGGCTCGGGAGGGCATGTGCGCCATCGCCATAGAGCCCTACTACCCGGTGATGTGATCTAAACAAGCTAGTATGCTTGGATAAGGATGCATGCATGGAATTAAATAAACTAAGCTCAACGAGACGGTGTGTGTGTGTG

>ZmCP07

GCAGCCTGCAAACATGAATGGCCCTCCCTCTCCTTCGTCGTCGTCTCGTTTATCCGTAGCGCTTCTTCTAATGATCACTGTGCTCGCATGCGGCTTCGTCTTGGCTTCCTCTGGTCGAAGCTACGCCCATGCTGACTACGCTGATGGAAGTGACCAAGAGCTGCTGATGTCGACGGAGTGGTTCCGCTTCCATGCCTGGATGGCAGCTCATGGCCGGTCATACCCGACGGCCGAGGAGAAGCTCCGGAGGTTCCATATATACCGTGCCAACGTGGAGCTCATCGAGGCCACGAACCGCGACACCAGCAAGACCTTCACCTGCGGCGAGAACCAGTTCACCGACCTCTCCCACCACGAGTTCCTCGCCATGTACACCATGGCCGGCCATTCGGCGCCGCCACTTCTAAACCTCTCGTCCGTGATCACGACCCGCGCGGGCGATATCACAGAGAGTGATCGCGGCACGACGCAAGTGGAAGAAGATGAAGAAGTAGAAGCTCTGCCGGAAAACATCGACTGGAGGGAGCAAAACGCGGTGACGCCTGTCCAGGATCAGCGCAGGGGCTGCAGTAAGTGACAAATAATATGATCGTAGAGTTGTAAAAAAAAACGCGCATCCTAGAATCAGGATTCTGACAACTATGTGGGCCGGGTTTTCTCCATGCAGATGCTTGCTGGGTGTTCGCTTCGGTGGCAACGATGGAGAGTGCACACAAGATCAAGACAAATCACGGGCATGGGGAGCTCCTAAAGTTGTCGGAGCAGGAGATCGTGGACTGCACCAGCCAACACTGCGGCGGCGGCTACCCGGATGATGCATTCAGTTGGGTGAAGAGGAATGGGATCGCCACGGAGTCGGAGTACGGCGGTTACGAGGCGACGGTGGACTCCTGCCGCGCCGACATGGTACGACCTCCGGCCGTCAGGGTCAAGGACTACAGTTTCGTGCCCAAAAACTCAGAGAAAAAACTAGCCATGAGGGTTGCCCAGCAGCCCGTCGCCGTCCTGTTCGACGCCACCGATCCCTGCTTCCAATGTACACGAATGGCATCTACTCGGGGCGGCCAGCAGCAGCCGCCGACCGCTATAATATCCTCAACCACGCCATGGCCATCGTCGGCTACGGGGAAGACAAGACTACGGGGCGAAAGTACTGGATCGCCAAGAATTCCTGGGGTACCCGCTGGGGACAAAACGGCTATGTCTACATACGCAAGGACATGGCCGACAGACCCGAGGGCGTGGGTGGGCTTGCCACACATCCCAGGTACCCTATCGTGTAATAGCTTAATTCCCATATATGTGTTGACAATAAAGGACAACGTACGGAACATGGTGGTGGAGCACCAGACTTTTGAGACGTCATTAAGACCTAATATATATAAATCAATAAATTAATATACACATCGCTGTTATGGTATTA

>ZmCP08

ATCATTTGTCTCTTCCATAAGCGTAGACCTACCACAGGGCTGGGCCAACCAGTCCCGATCGATCAGCCAGCATGGCGTCCTCCTCCAAAGGGTCGCTGCCATGCGTGCTCCTCCTCCTCGCTGTCTTCCACCACGGCTGCTCGTCGGCGAGGGCCCACAGACGTGCCGGCGACATGGAGAGGAGCATGAGCACCGACGACAGCTCGATGATAGAGCGGTTCCAGCGGTGGAAGGCGGCGTACAACAAGTCCTACGCCACGGTCGCCGAGGAGCGGCGGCGGTTCCGGGTGTGCGCGCGCAACATGGCGTACATCGAGGCCACCAACGCCGAGGCCGAGGCGGCGGGGCTCACGTACGAGCTCGGCGAGACGGCCTACACCGACCTCACCAACCAGGAGTTCATGGCCATGTACACGGCGCCCGCGCCGGCGCAGCTGCCCGCGGACGAGTCGGTGATCACCACGCGCGCGGGGCCGGTTGACGCCGTCGGGGGCGCGCCGGGCCAGCTGCCGGTGTACGTGAACCTGTCCACCTCCGCGCCGGCGAGCGTGGACTGGCGAGCCAGCGGCGCCGTGACGCCGGTGAAGAACCAAGGCCGATGTGGTACGCACAATCCGTTACCGACACCATCATCATTCCGCGCGTCCTTCCTGGCACCTGTCATTTGCCACCAGCTGTTGCTCTTGCCTGTCCCACATGCATGCATGCTTGTCACTGCGCAAAACGCACATGCTTTTGTCCTGGATGGACGGGTGACGGGATCGGATCGTGCACGCAGGGTCATGCTGGGCCTTCTCGACGGTGGCGGTGGTCGAAGGCATCTACCAGATCCGGACGGGGAAGCTGGTGTCCCTGTCGGAGCAGGAGCTGGTGGACTGCGACACGCTGGACGACGGCTGCGACGGCGGCATCAGCTACCGCGCTCTGCGGTGGATCGCCTCCAACGGCGGCATCACCACGGAGACCGACTACCCGTACACGGGCACCACGGACGCTTGCAACAGGGCCAAGCTCTCCCACAACGCGGTCAGCATCGCCGGGCTCCGGCGCGTTGCCACCCGGAGCGAGGCGTCGCTGGCCAACGCCGTGGCGGGGCAGCCCGTGGCCGTGTCCATCGAGGCCGGCGGCGACAACTTCCAGCACTACAAGAAGGGAGTGTACAACGGGCCCTGCGGGACCAACCTCAACCACGGCGTCACCGTCGTCGGCTACGGCCAGGAGGCGGCCGGCGGCGACAGGTACTGGATCGTCAAGAACTCGTGGGGGCAGGGGTGGGGCGACGACGGATACATCAGGATGAAGAAAGACGTCGCCGGCAAGCCGGAGGGGCTCTGCGGCATCGCCATCCGCCCGTCCTATCCACTAATGTGACATGGTGTGGTGTACATCTGTTTTCGTTTTAATTATCATTTTATTTTATTAAACAATAAATAAGTAGTCAACATATGGATCGACTCAAACTTTTGCAGTTACGTGTACCAGTACACTTGTTGTAAATAATAATCCATGGCATCTCGCGTGTAAACTTTATTCTGGTGCGAAAACGACTTCTTCAAA

>ZmCP09

ACTACAAGTACTTCCACATTCCAACAATCATCGCGCCTCCGTGCATGCACCGTTGCCGATCGTCGTCCTCCTCCCCACTCCCCAGAGGCCAAGCCAAAGGTCCAGCAATGGCGAGGTCTCCCCGGCTTCTCGCGCTGCTGCTGGCTGTCGTCTGGATATGCGGCGCGGCCCTAGTAGCGCGTGCCGACCCGATGCTCGAGAGGTTCGAGCAGTGGATGGGCAGGCACGGCCGGCTGTACGCTGACGCCGGAGAGAAGCAGAGGCGGCTCGAGGTGTACCGGAGGAACGTCGAGCTGGTCGAGACCTTCAACTCCATGGGCAACGGGTACAGGCTGGCCGACAACAAGTTCGCCGACCTGACGAACGAGGAGTTCAGGGCCAAGATGCTGGGCTTCGGACGGCCTCGCAGCGGAGGCGGAGCCGGGCACTCCACAGCGCCGAGCACCGTGGCTTGTGTCTGTACTCTCTTTCTCTGAAACCATCTGAGCTAGCTTGCATTGTCAGTACTCAGTAATCGCGGCAATCCACGCAAGTTTCTCAGCCATCCCATGTTAATTAACTTTTTCATGATGTTCGTGCTGGTTATAGATTGGAAGTGGACTGATGGGTCGTCAGGGTTATTCTGATCTACCGAAAAGCGTGGACTGGCGAGAGAAGGGAGCTGTCGCGCCGGTGAAGAGCCAGGGGGACTGTGGTGAGTGATATATAGCGTTCGTCGGTGAACGATGGATGATCCGGAGAATGACGAGTGCGATGCCCGCCTCGCCTGGTTTGATGAGACTTTGCGCGCGCGTCCTGCATGCAGGCTCTTGCTGGGCGTTCTCCGCCGTTGCGGCCATCGAGGGCATCAACCAGATCAAGAACGGGAAGCTGGTGTCCCTGTCGGAGCAGGAGCTGGTGGACTGCGACACCAAGGCCATCGGCTGCGCCGGCGGGTACATGAGCTGGGCCTTCGAGTTCGTCATGAAGAACCGCGGCCTCACCACCGAACGGAACTACCCGTATCAAGGTACACGGGAAACGTACCAAGAGATCAAAGATTTGCATTTCTAGCTAGCTCCACGTCCGTTGCGTTCGCGGTGCAGGGCTGAACGGCGCCTGCCAGACGCCGAAGCTGAAGGAGAGCGCGGTGAGCATCTCCGGGTACATGAACGTGACGCCCAGCAGCGAGCCGGACCTCCTGCGGGCCGCCGCGGCGCAGCCGGTGTCCGTGGCCGTGGACGCCGGCAGCTTCGTGTGGCAGCTCTACGGCGGCGGCGTCTTCACGGGACCCTGCACCGCCGAGCTGAACCACGGCGTCACGGTGGTCGGGTACGGCGAGACCCAAGGGGACACGGACGGCGACGGTAGCGGCGTGCCCGGGAAGAAGTACTGGATCGTCAAGAACTCGTGGGGGCCCGAGTGGGGCGACGCCGGCTACATCCTCATGCAACGCGAAGCCAGCGTCGCGTCTGGCCTCTGCGGCATCGCTATGCTCCCGAGCTACCCGGTCATGTGATCCGGCGGCTGCCGCCGTCGAGACCTACAGCTGCAGCTCTAAGAACAATCAGTCATTATGTTGGCCATCTTTTGAACAAAGGGCGTTCCTCTTTCCCG

>ZmCP10

GGGAGAAGGGAACCGAAACCAAGCAACCCAATCCAAACCAGACAAGCAAGGAATCCCCCGATGGCTCATCGCGTTCTCCTCCTCCTCTCTCTCGCGTCGGCCGCCGCGGTTGCCGCCGCCGTCGACGCGGAGGACCCGCTGATCCGGCAGGTGGTTCCCGGTGGAGATGACAACGATCTGGAGCTGAACGCGGAGTCCCACTTCCTGAGCTTCGTGCAGCGGTTCGGCAAGTCCTACAAGGACGCCGACGAGCACGCGTACCGGCTGTCCGTTTTCAAGGCCAACCTGCGCCGCGCGCGCCGGCACCAGCTGCTGGACCCGTCGGCGGAGCACGGCGTCACTAAGTTCTCCGACCTCACGCCGGCAGAGTTCCGCCGGACCTACCTCGGCCTCCGCAAGTCCCGGCGCGCTCTCCTCAGAGAACTCGGGGAGTCGGCGCACGAGGCGCCCGTGCTCCCCACCGATGGTCTCCCCGACGATTTCGACTGGAGGGACCACGGCGCCGTCGGCCCCGTCAAGAACCAGGTCGGTTTCCAATCTGTTGACCATGGATCCACAGATCGGAGCAGTTCTTTCATAGTACTCAGCGATCTGTTTGGGTCCTAAATTTCCTTTCCCCGGCTGTTGTTTAGGGTTCGTGCGGGTCGTGCTGGTCGTTCAGCGCGTCTGGAGCGCTGGAGGGTGCGCACTACCTCGCAACTGGCAAGCTGGAGGTGCTTTCGGAGCAGCAGTTCGTCGACTGTGACCACGAGGTGAGGAAGGAAACTGCCCTTTTCTTTCATCCATAGATTAAATTCATAGTTTTCTTACTATTATCGTACTTTTTTATGGGATTGGTTCATGGTCACATGTGCTAGGAAGGCTGGGTTTCCGGCCATAGCCCATGTTTTGTAAGCTAGTGTGATGTCCTTTTCCTATTTTCCTCCTTTCCGATTGGGCAGTGGTGCACCTACCGTGAGTCTGAGTAGGAAAACGGGGGTTTGGAAGAATGGAAAGGTTTCATGATAATTTTTCAAAATAAAATTAGCGGCTAAAAGAGTGGTACAATATTGAGATGGTAGAGCATCTCCGACCATCTTTTTTAAAACTCACCTTTCATCATCTTCTGCACACTTTGTGGTTGCCTGCCCGTTCTCCATCTTTGTCTAGCTAGCTAGAAATAAGGAATAGATGATAACAATATTTGGAGATCTAATTGACATAAGGATATAGTGAGGCTCTTGGAGTTGCTTACTCCTTGAGGGGTGAATCCACAGTGATTCAAGTTTGGATAACAAATTACTGTCTTAGTTTACTGATAGAGTAGGTTGATTCACTTGACCATGGAGCTGCTGGCGTTATTTGTATGTCTTATAGAGTTATAGTCTTTGTAGATCATACATCTTGTCTTCACAACTAAGCTCTTATGGTTCGGAACCTGTGAGCCTTCGGGGCCTTGTGGAATCTGACTTCATAAGTTCCGAACAGTGGGGTATAATTTGAACAAAAGCATGGTGATAATATTGTACATCCTAGGATTTGACATTTCATTTCTCATGTGTCAGTTCTATAGTTAGGTTTGTTCTTGAACAGCTGACTATCTGAGTGGGGCTTGCTCCTAGGAACATGGGATTGCAATGAGTTGGCTTACTATTGTAATAACCATCCATTATTCAGCCTGGGTAGGCGTTTTTGACAATCTTCTGGAACATTATTTGGCTATTTTGAATGCAAAGTTTTTGAATCATCGTTATATGCATAGACTATCGCTACATTCATCAGCAAGATGCAATTTCACTGTACATGGTTTTCAAGTCATCGCAAGGGGGCGGCACCAGCATGGGGGAAGGGGAGGGGAAGCGGCAAGGACTGGAGGAGAGGTGATGGCGGCAGCAACAGATGTGAACGGGAGGGGGTAGGGGGATGTATAAAGAAAGGGGGAGGAGGGAGGGAGAGAGGTGGCACCTGATGGAAGGGTCTCCGGAGAGTGACATCACTAGGTCGTGCTTAGGGAAGGTTATATGGGTTGTTAGGTGGTCTTTTGGACTGGACTTCCTGAGTTTCTTCCCCTTCTATTTTTTCTTTTCGTTTTCGTACCTTCTCCACAATGCAGTACTGAAGTTCATATTCTTATGGCGTTGCCTCACCTCGCCTTATGATTGCCTAGGTGTCGCGTAGTCGACTCAAAAGGGTGGCTCGCCACATCTCATCGTACCGCCTTAATAACTATGATATCATATAACCAATATCTGATCCAGTGATGTAAATTTTTCATTTTGCATGAGGCTTTTGCTAATTTATCTTGAATGTGACAGTGATTTTGCAGTGCATGAGTATTGCACATGTGTGAAGCTAATGCCATGAGACGGGACCCATATATTTGTAGGTTTTGGACAGTAGGATTTGCATAGTTGTCTTGAAGTTAACTGTTACCTTATTTAGCTCACATTAATTCAAACTTACTATCCCCCATTCCTGTTGTGAATGTTGCAGTGTGATTCATCGGAGCCTGATTCATGTGATTCGGGTTGCAATGGTGGGCTAATGACGACGGCATTCAGTTATCTCCAGAAGGCCGGTGGCCTTGAGAGTGAGAAGGATTACCCTTACACTGGGAGTGATGGCAAATGCAAGTTTGACAAGTCCAAGATTGTTGCTTCAGTTCAGAACTTCAGTGTTGTGTCTGTGGACGAGGCTCAAATTTCTGCTAACCTCATCAAACATGGGCCATTGGCAAGTAAGTAAAAAAGACCGAAAGAATCTTTTGCCCCAATAGATCTGACCACCTTATCAAAATACATAGAACAGTCAGTTGATGCAATTTCTCTGTTCCACGCAGTCGGCATCAATGCTGCCTACATGCAGACATACATCGGAGGCGTGTCATGCCCATACATCTGTGGCAGGCACCTTGACCATGGTGTTCTCCTTGTTGGATATGGTGCTTCTGGTTTCGCCCCCATTCGCCTGAAGGACAAGCCTTACTGGATCATCAAGAACTCGTGGGGCGAGAACTGGGGGGAGAACGGATACTACAAGATATGCAGGGGTTCCAACGTTCGCAACAAGTGCGGCGTCGATTCCATGGTCTCTACCGTGTCCGCAGTCCACGCCTCGAAGGAGTAGACTCTGGTCAGCGGTGATAATCGTGATTCCATATATTGTAGAACTATAATCCCTGCTAGTAATCCATTTGTGCTGTTTATGTATCGAAGGCAGCAAAGATGTGTGAAGCGGCAATGGGATGCTTGCTGTAAGGTAATATGTTTGGCTTGCTAGGCATGCTACACGCTATTTGGATGTAGCAACGTTATCACCAGCTGTATACGCTTTTTAATCGACAACATTTGAATAATATGTA

>ZmCP11

CGCTCGTGCCAGCAGGCCATTCTTCTCGCCTTACTCCCTCACAGAACCCAGTAAAATATTGCCAGTCCCGCCGTCGAGATGGCCCCACGCCGCCTGCTCGTCCTCGCCGTCGTCGCCCTCGCGGCCACCGCCGCCGCGGCCAACTCCGGCTTCGCGGACTCGAACCCGATCCGCCCCGTCACCGACCGCGCGGCCTCCGCGCTCGAGTCCACGGTCTTCGCCGCGCTCGGCCGCACCCGCGACGCGCTCCGCTTCGCACGCTTCGCTGTGAGGTGAGCACCCAGTCACCTAATCCAGCGGCCAGCGGTCCAAACCCCGCCGCCGTGCGCCCTCCCGTTTCCATTCTCCATGATGGCCTCACGATTTCTGCGGTTATGCCGCCGCCGTCTCGCGATACAGGTACGGCAAGAGCTACGAGAGCGCGGCGGAGGTCCACAAGCGGTTCAGGATCTTCTCCGAGAGTCTCCAGCTCGTCCGCTCCACCAATCGGAAAGGCCTCTCCTACCGCCTCGGCATCAACCGTATGACCTTGACCGCCTCAGTCCTCCTCGCCCCAATCTGTAACCGTGGGCTTCAGCGCGTCCCGGATTCTCATATGTTTGTTATGTGTGGCTGGTGTACAGGCTTCGCGGACATGAGCTGGGAGGAGTTCCGTGCGACCCGGCTCGGCGCAGCCCAGAACTGCTCCGCCACGCTTACCGGCAACCACCGGATGCGCGCCGCCGCCGTTGCGCTGCCGGAGACGGTGAGTTACCAAAGCTGTTCGTCGGTCGTCACTCGTCACAGATAATCCTCTCTGTCTTGTAACGTAACGTTTTGACTCCCAAACGGGCAGGCGAGTAGGTTGTAGCCACGTTGGTACTGTGATTTCATTTGTAACGGCATGAGACGGCCCTGTGTTGGTTCAGTTTATTTAGGCTTTACCGCTTTACAGGGGGGTTCGCAGCTTGTCTGTGGGCCTGTGGCTGCAGTATTTTCTCGTTGTTTTCATGCTTTGTGGTCTCTAGCTTTTGCCTATTTGCTCTGCCGGTCCCACCATGACAAGCAATGTTTTTCAGCCACTTCCTTCCTTGGCCCCATCACATGATGAAGTTAATGGTCCCTTTCTTTGCAGTACTAATTGCAACAGTGGAACTTCTCAAATTTGGTTTATGTTTCCAACAAAACCGCAAAATTGTTTAGAGCCTCTTTGGGAGAGCTCTTTCTCAGCTCCGGCTTCCCCGGAGGAGCTCTACCAAACTATTTATAGGAAGAGCCATTTTCAGCGAAAAACAAAAGCCCTACCAAACAGTTTATAGGAAGAACCATTTTCAGTGAAATACAAAGGGGCCGTAGCTGCTTTTGAGGAGCCACAAATTAGCTTCTCCAAAACGGCTCCAGCTCCCCTCAAGGAGCCCCTCACGAGGTCTGCCAAACACCCCCTTAATTAACTAGCTGGTGCCTTGTTAGCAACCATGTATCTTTAATGATTATAGAACAGCAGTTGTGAGATACATGCTTACTAGTCCAATAGTGGTTTTCGCAGTTTTGCAAGGCGTTGCTTTACTAGTTCTGAGCCATGTGACGTGGTTACAAATTAATACTTCAACTAGTCTTGGATGCTATAGTTTTTTTGGGCATTATGATGTGAAATGTGTATGTGATGTTGGTCTGGTTACTGCAGAAAGACTGGAGGGAGGATGGGATTGTGAGCCCAGTGAAAAACCAGGGCCACTGTGGATCATGCTGGACCTTCAGGTAACTACTGAAAAATCAACATCCACTTTTTCGTTCTCTTTTTTCTTATATATATCATTCCTAGAGGAAACTGGATGGAACATATGTTATTACTGGTTTACCTTGTTCTCTAATGTACTAAAAAGTAATAATATATGAGAATCATGTCAATGATGGTTGTGTTTGGTGCATGCTTACTTTACCAGTAAAAGGTGCAGATGGGATCCGACAGGAAGTACAGATTGGAGTCTGTATCCAAATTACTTGCATCGTCTTCATGTCCTTTAAATCAATCATGGTCTTGAAAGTAATCAGGGTGGGGCACATTCAAACCATGCTGATAAATAATTTAAAACTACTTTGGGTGTGGTCCAGGATGGGTTGTGATAGATATATCTGATGTTTTTGTGTAAAAAAATAGATATATCTGTTGTTGAAGCTGATTTTTGCTAGGAGTAGTACTTGATATCTTTCGTCCCAGAGTTTTTTTTAACGAAAGAGATTTTAAAAATGTCCAGTGATTGATAAAAACCTACTATACTCGTGAGACATGCAAGCAATTAAATAATTGGATCACGTTCAGATCTGTCCCAGTTAGTCGACCTAAACTAATGCACTCGTGAGACACGCAAACAATTAAATAATTGGATCAGGATCAACTAACCAACCTTAGCTCTTGGCCTCTCTATGCTTTGCATTCAAATGTCCCGGTTAGTTGACCTGATCAATTTTGGAGCTGTTCATGTTTCCTGCTAACTAAATTTACTTTCAAATAATAGTTTCCATTTCATATTAATGCATTATGAAGTTTTATATGCACACAAGCTAATATGTCATATGACATACATTACTATCCAATAGTGTAATGCAGACCTCCATGAAAAAGTTTGCCTGATGAATTTTAAAAAGATCAATTAATATGTAAAGCAATGCAAGTCTAATTGTCCAGTCGTTCCGAGTAACAAATTAATATATCAGATTTGAGTCATGATAGTGAGCAGAATCTCTAATAAATGTGATCCCTTGTAATAAAAGTTGGTTATTAGACAATATCCTTGAGACTGTTTATTCATAGTGAATAGTTAAGTTAGTATCAATCTCATCTTGTGTTTTCAAACATTTCTGTGTTTATATACTATCCTTCTGGGCCTGCCAAAAAAATTATAAAAAATCACAGTTCAAATCTGGGAGAAGCAAACTTGGTACATAGATAGAGAAACAAACCACTTCAGAGATAAGAAGCCAAGTTATATTCTTAATTGTCAGAGCATTGCAAACTCTGGTTACTTCCTTGATTCATGCCAATCTATATGAGAACAAATTCAGGCTGCAGGGTTAATAAAGTTTTATTCAGAGAGTTTACATGTAGTTTGGATTTTGTTGATTTAATGTCTTCATTTCTCTTCTGATATTCCTATATCAGTGAATCTGAATAAATATCATTTTTACCATTGGATTCTCTCACTGGTATCCAGTTTCAAACTGTAGGCTGTAGCCAAAATAAGATTGGCCAACAGCCTTCATGGAAAAGGAAATGATCCTAAGAAAGTGTTAGCTAGCACTTATACATCAATTCTGAAGTAGTGCACATTTGTATTATCAGATATGCCTCACAAACAAAGAGGCAAAGGGTAAACCTGTAGGAGAGCTTTCATGGAATGTTGAGGTGCAGAAACTTAAGCAATTAAGCATTTGAATATAATTCAGATAATTTTTAGCAGACAGTTATTAGACGTTCTTATATTTCGTGTTACATGTTCTTTTCACTCTTTATCTTTTCTTCTGCATAGAAATGACCATTATAGCATGTTCTGAGAGTAGAAAAAGGGGGTCGTGCATAAAAATAATTAAATTCGTTTGATGCTTATTATGGCAGCACTACTGGTGCACTTGAGGCTGCATATACCCAGGCAACTGGCAAGCCCATCTCTCTCTCTGAGCAACAGCTTATTGACTGTGGTTTTGCATTCAACAATTTCGGATGCAACGGAGGCCTTCCATCCCAGGCCTTTGAATACATCAAATACAATGGTGGCCTTGACACTGAGGAATCTTACCCTTACCAAGGTGTCAATGGAATCTGCAAGTTTAAGAATGAGAATGTTGGAGTCAAGGTTTTGGACTCGGTTAACATCACCCTGGTGAGAACTTCATCCTAAATTTTAGCAGAGTGTTATCTACAATTAGGGTGTGTTTGGTTTATAGAGATAAATTTTAGTCCCCTACTTTATTCTATTTTAGTCCCTAAATTACCAAATACGCTGTATTTGGCAATTTAGGAACTAAAATGGAATAAAATGGAGGGACTAAAAATTAGTCCCTATAAACCAAACACCCCCTTAGCTACTCTGAGTTAATAGTTCATACTATTTCTGTTGGTGTGGTTAGGGTGCTGAGGATGAACTGAAGGATGCTGTTGGTCTGGTTCGCCCAGTTAGTGTTGCCTTCGAGGTGATCACTGGTTTCAGGCTGTACAAGAGCGGAGTTTACACTAGCGACCATTGTGGAACTACACCGATGGTCAGTTGCAGTATCCTTGTGTCATCAACCTACGATTTATCTATGGTTTGCTTATGTAGAGTTCCTCTATGAATCGCTCCTATCCTATCATGCCAGGACTTCTTGTTATCCTGTTGTTCTGATCTGAATTTATGAATTCTTTTGCGTTACTAGGATGTGAACCACGCTGTTCTGGCTGTTGGCTACGGTGTCGAAGATGGTGTACCCTACTGGCTCATCAAGAACTCATGGGGCGCTGACTGGGGTGATGAGGGTTACTTCAAGATGGAAATGGGCAAGAACATGTGCGGTGAGTATTCCATGCATTTCCTCGCTACAATAAAGAACGAGAAGAGATCTTGTGTTTTCAGGTATGGTGCTCTAGCACTAGTGACACGATACTGACTGACCCTAAACGATGCAGGTGTTGCTACGTGTGCATCCTACCCTATTGTCGCATGAGGCCCTTACGAAGTGTTACATGGTCTGTTTGGCATCAATAATGCATGTTTAACCTGAGCTTGGCGATGGGTTATACAGAACGGAAACTCTGTTTGTGAATAGAAAATCATGAAGGGAAGGAGTTGACCGGACCCCTGCTTGTACGTCTCCCCGACTGTGTAGGTAGATTGTCAGTTGGGGTTCCGAAGTACTCTACTCATACGTGTGTATGACAGTTTATTTATGAAACAAAATACGCATTGATCGTTATGGTGCAGCCTGGGATTTCTTATTATAACATAATGTATCGTATGTATAAACGGACCGTCCTCAGCACGGACCTAATATAAGCACGACCTGTTGGCATCA

>ZmCP12

TGCACAACTGGCTATCATGGCGCCCCACATTGTCGTCAACAAAACCGTGATCACATTCACAGCGGTGGCCCTGACGATTCTTGCTGTGACAACCATGATGGCCGAGGCCCGAGACCTGTCATCCACCTCCACCGGTGGCTATGGCGAGGAAGCCATGAAAGTGAGGCATCAGCAGTGGATGGCGGAGCATGGCCGCACCTACAGGGACGAGGCTGAGAAGGCGCACCGGTTCCAGGTATTCAAGGCGAACGCCGATTTTGTTGACGCGTCCAATGCCGCAGGTGACGACAAGAAGAGCTATCGGCTGGAGCTCAACGAGTTCGCCGACATGACCAACGACGAGTTCATGGCCATGTACACTGGCTTGAGACCAGTCCCGGCAGGGGCAAAGAAGATGGCCGGTTTCAAGTACGGGAACGTGACTCTCTCAGACGCCGACGACGACCAGCAGACGGTTGACTGGAGGCAGAAAGGCGCGGTCACCGGTATCAAGAACCAGGGCCAGTGTGGTAATAATTATATATTCGATGATTCAATTTTATTCCATACAGTAGACTGTAAGTAATGCTCTAATAAAATGCATGCATGCATGCATTGTGCAGGTTGTTGCTGGGCCTTCGCTGCGGTGGCGGCCGTGGAGGGCATCCACCAGATCACGACGGGCAACTTGGTGTCGCTATCGGAGCAGCAGGTGCTGGACTGCGACACCGACGGGAACAACGGCTGCAACGGCGGCTACATCGACAACGCCTTCCAATACATCGTCGGCAACGGCGGCCTCGGCACCGAGGATGCCTACCCGTACACCGCCGCGCAGGCCATGTGCCAGTCCGTGCAGCCGGTGGCCGCCATCAGCGGCTACCAGGACGTGCCGAGCGGCGACGAGGCAGCGCTCGCCGCGGCCGTCGCCAACCAGCCGGTGTCGGTTGCCATCGACGCACATAACTTCCAGCTCTACGGTGGCGGGGTGATGACCGCGGCCAGCTGCAGCACGCCGCCGAACCTGAACCACGCCGTCACGGCGGTAGGCTACGGCACGGCGGAGGATGGGACCCCGTACTGGCTGCTCAAGAACCAGTGGGGCCAGAACTGGGGAGAGGGAGGATACCTCAGGCTCGAGAGAGGCGCAAACGCCTGTGGTGTCGCCCAGCAAGCCTCCTACCCGGTCGCATGATGAATAAAGCATATATTCTACATATACACGAAGGCATGCATAAAAATGGCTAGTAAGGAATAAAGCATGCAAACACATTGACAGTACATACATACATAGCTTAAAATTGAATCACAATTGTCATGTACAACACTCCAGATATCATTTATTTCTATTTGGCCTTCTTTTCAAAGATCGATATTCTATATATACATATATGTAAGGGAACAAGTTTTTATAGCAT

>ZmCP13

GCACTAGCTAGCTCTAGCTTCGCCATTGCATTCGCTTACTGGGAGTACGTCTGGTACTTGCACAACTAGCTATATATATCATGGCGCCCTACATTGTCGTCAACAAAACCGTGATCGCATTCACAGCGGTGGCCCTGACGATTCTTGCTGTGAAAACCATGATGGCCGAGGCCCGAGACCTGTCGTCCACCTCCACCGGTGGCTATGGCGAGGAAGCCATGAAAGTGAGGCATCAGCAGTGGATGGCGGAGCACGGCCGCACCTACAGGGACGAGGCTGAGAAGGCGCACCGGTTCCAGGTATTCAAGGCGAACGCCGATTTTGTTGACGCGTCCAATGCCGCAGGTGACGACAAGAAGAGCTATCGGATGGAGCTCAACGAGTTCGCCGACATGACCAACGACGAGTTCATGGCCATGTACACTGGCTTGAGACCAGTCCCGGCAGGGGCAAAGAAGATGGCCGGTTTCAAGTACGGGAACGTGACTCTTTCAGACGCCGACGACAACCAGCAGACGGTTGACTGGAGGCAGAAAGGCGCGGTCACCGGTATCAAGAACCAGGGCCAGTGTGGTAATAATAATTATATATTCGATGATTCAATTTTATTCCATACAGTAGACTGTAAGTAGTGCTCTAATAAAATGCATGCATGCATGCATCCATTGTGCAGGTTGTTGCTGGGCCTTCGCTGCGGTGGCGGCCGTGGAGGGCATCCACCAGATTACGACGGGCAACTTGGTGTCGTTATCGGAGCAGCAGGTGCTGGACTGCGACACCGAGGGGAACAACGGCTGCAACGGCGGCTACATCGACAATGCCTTCCAATACATCGCCGGCAACGGCGGCCTCGCCACCGAGGACGCCTACCCGTACACCGCCGCGCAGGCCATGTGCCAGTCCGTGCAGCCGGTGGCCGCCATCAGCGGCTACCAGGACGTGCCGAGCGGCGACGAGGCAGCGCTCGCCGCGGCCGTCGCCAACCAGCCGGTGTCGGTTGCCATCGACGCACATAACTTCCAGCTCTACGGTGGCGGGGTGATGACCGCGGCCAGCTGCAGCACGCCGCCGAACCTGAACCACGCCGTCACGGCGGTAGGCTACGGCACGGCGGAGGATGGGACCCCGTACTGGCTGCTCAAGAACCAGTGGGGCCAGAACTGGGGAGAGGGAGGATACCTCAGGCTCGAGAGAGGCGCAAACGCCTGTGGTGTCGCCCAGCAAGCCTCCTACCCGGTCGCATGATGAATACGTAAGGGTCGAAAAGAAAACATATATTCTACACACACGAAGGCATGCATAAGAATGACTACTAAGGAATAAAGCATGCAAACACATTGACAGAATGACAGTACATACATGCATAGCGTAAAATTGAATCACAATTGTCATGTACAACACTCAAGATATCATTTATTTCTATTTGGCCTTCTTTTCAAAGATCGCTATTATATATATACATGTAAGGTAACAAGATTTTAT

>ZmCP14

AAACAACTGCACTAGACATCTCAATTAATTAGCTAATGAGCAAGCGGCTGAGCTGCACTCAAGACAAACTACCATGGATCAGTCAAACATTAGCAACAAGCACATGACGATGACAACCCTAATGCTCCTCCTCTGTGTCATAGCCATTGCAGATTGCATTTGCCACGCCGCAGTGGCAGCCCGGGTGGAGCCATCCACCACCGTCGGCAGAACTACAGGAGGAGACGAGGCGATGATGATGGCGAGGTACAAGAAGTGGATGGCGCAGTATCGCCGGAAGTACAAGGACGACGCCGAGAAAGCACACCGTTTCCAGGTATTCAAGGCGAACGCGGAGTTTATTGACAGGTCTAACGCTGGAGGAAAGAAGAAGTACGTCCTAGGGACCAACCAGTTCGCCGACCTGACCAGCAAAGAGTTCGCGGCCATGTACACCGGTTTGAGGAAACCGGCGGCGGTGCCTTCCGGGGCCAAGCAGATCCCTGCAGCTGGTTCCAAGTACCAGAATTTTACGCGCCTAGATGATGATGTCCAGGTTGATTGGAGGCAGCAGGGTGCTGTCACTCCTGTCAAGAACCAAGGCCAATGTGGTAAAGTTAACTGCTCCGTCTACGTCTATCATTATTAACTATTGAGTAACTGGCTAATGGCATCTAAATGAGATCAATATTTGCAGGCTGTTGCTGGGCGTTCTCTGCAGTAGGTGCCATGGAAGGTTTGATCATGATAACGACAGGAAACCTGGTCTCCCTGTCGGAGCAGCAGATTCTAGACTGCGACGAGTCAGACGGGAACCAGGGCTGCAACGGTGGCTACATGGACAACGCCTTCCAGTACGTCATCAACAATGGCGGCGTCACCACTGAGGACGCCTACCCTTACTCTGCAGTCCAAGGGACGTGCCAAAACGTCCAGCCAGCCGCCACCATCAGCGGCTTCCAGGACCTGCCCAGCGGCGACGAGAACGCGCTCGCCAACGCAGTCGCCAACCAGCCGGTGTCTGTTGGCGTCGACGGCGGATCGAGTCCTTTCCAGTTTTACCAGGGTGGCATATACGACGGGGATGGCTGTGGCACGGACATGAACCATGCAGTGACGGCGATCGGCTACGGCGCCGATGACCAGGGAACCCAGTACTGGATCCTCAAGAACTCCTGGGGCACAGGATGGGGTGAGAACGGTTTCATGCAGCTACAGATGGGCGTCGGCGCCTGTGGTATCTCCACGATGGCCTCCTACCCAACTCCATGAACACAAATCTTCAGATAAAGCGTAGTATATACCAGTCCATGCATATGACTGCAGAATAAAGCATGGAAGATTAATTACATGCGTGCATCAAGGCATGCTACGTACTGTATTAAAACTTCGGTACTGGCCAGATATATACACAAAGGTAGAATGTTTGTGTGATAATATAGTAATGCTTGTACGAATGCAAAGAACATGACCTTTCAATAATAAATAAGATTAGGCT

>ZmCP15

CTAACACTGACAATGGAGTCACAGTTAACCATGCCTAACTTAAATCCTTTTGGGTCCTTTCCGGCTACCTCCTGAACGTACTGCTATATAAACCCCTTCCTGCATTGCAGGGTTTGGCCAAGCACCAGCTAAGCTAGCTAGGTCACATGACATGAGCTAAAGACGACGACACACTAGCATTGCACACACAAGACACACTAACTACATAGCTAAATACACTAGCATACCCCCCCAAAAAAATATGTCCGCGTCACGGTTCCTCCTCGCCGTCCTCGTCGTGGGCAGCGCCGTACTGTGCACCGCCGCCGCTCCCCGCGCGCTCGCGGCGGCCGCCGCCGCGATGGCGTCGCGGCACGAGAAGTGGATGGCGGAGCACGGGCGCGCGTACAAGGATGAGGCCGAGAAGGCGCGGCGGCTGGAGGTGTTCCGGGCCAACGCGGAGCTGATCGACTCGTTCAACGCCGCAGGGACGCACAGCCACCGGCTGGCGACCAACAGGTTCGCCGACCTCACCGTCGAGGAGTTCCGCGCGGCCAGGACCGGGCTCCGGCCGCGCCCGGCGCCGTCCGCGGGCGCCGGCCGGTTCAGGTACGAGAACTTCAGCCTGGCGGACGCGGCGCAGAGCGTGGACTGGCGGGCCATGGGTGCCGTCACCGGCGTCAAGGACCAGGGAGCGTGCGGTACGTACCTACGAACGAGAACGACCATGCAAACACGGAAACACCACCTGCGCACATGCACGATGCATGCATTCTGAATAGTGAACGATCAAAACCTACCGTGCGTGGCCGTGGATACGTCTCCATCTAGCTACCTTTTTTCTGAATGAAGGACCAATTAAGTTAAAAGGTTCCCAGTATTTTTAGGCAATTAATCCAACGTAGCCATGCACAACACGTCAACACACACGCACGCAGGCTGCTGCTGGGCGTTCTCGGCGGTGGCGGCGGTGGAAGGGCTGAACAAGATCCGGACGGGGCGGCTGGTGTCGCTGTCGGAGCAGGAGCTGGTGGACTGTGACGTCAGCGGCGTGGACCAGGGCTGCGACGGCGGCCTCATGGACAACGCCTTCCAGTTCGTCGCGCGCCGCGGCGGGCTGGCCTCGGAGTCGGGCTACCCGTACCAGGGCCGGGACGGGCCCTGCCGCTCGTCCGCCGCCGCCGCGCGGGCCGCGTCCATCCGGGGCCACGAGGACGTGCCGCGCAACAACGAGGCCGCGCTCGCGGCCGCCGTGGCCAACCAGCCCGTGTCCGTGGCCATCAACGGCGAGGACATGGCGTTCCGCTTCTACGACAGCGGCGTGCTGGGCGGGGCCTGCGGCACGGACCTCAACCACGCCATCACGGCCGTCGGGTACGGCACGGCGAACGACGGCACAAGGTACTGGCTGATGAAGAACTCCTGGGGCGCGTCGTGGGGCGAGGGCGGATACGTCCGGATCCGCCGCGGCGTCCGCGGGGAGGGCGTCTGCGGTCTCGCCAAGCTGCCGTCCTACCCCGTCTAGAGCGCCGATCGCGTCCACCAACGTAACCATCGCACAGCATACAGTACGCATGATGCTGTTACGTCATCCAAGACAAGCGTGCTCGTGCATGGCATATATATTTGACTCCTCCATTTTTAAACAAGCGAACGATAAATATTCGAGAATGGAGATAATACTTATTATAAGATTGTATATAGGCCCTGTTTGGGACAACTGCTGCTTGTTGAAAAAGCAGCTTATCTGATAAGCTGGTAAGAAGCAGCTTCTGCTTGTTGGCTGCTTTTAGTTCATTTTGAGAAGCAGCTGAACTGATAAGCTGCTGCAGAAGCTGACTGTTTGGCAGGACTTCAGCTTAAATTTGTGAAGAAGCTGAAAATAAGCTGTGCCAAACAGGGCCATAATCACATGATAGATGGTTATATGTATAGTGATATAGTCGAAATCACGACCCACGCCAAGCACTATGTATTGTACTCCCAGCCATAAGAATGTCGCTGAGGTATTAGTTGGACCTTTACGTGTGATGGTCACGTGTATTGTAATGTACAGATTGCCTTGAATGCAACCAATTTAGTGTAAACAAATACATTCA

>ZmCP16

AAGCCTAACGTAGTAAACGCCATGGCTACTCACTACTCGTCGGCTTTCGTCCTCCTCTCCGTCGTCGCCTGGGCCTGCGCTCTCAGCGGCTCTCTGGCGGCGCGCGACCTGGCGGACCAGGACCAGGCCATGGTGGCCAGGCACGAAGAGTGGATGGCAAAGTACGACCGCGTCTACAGCGACGCCGCGGAGAAGGCGAGGCGGTTCGAGGTGTTCAAGGCCAACATGGCCCTCATCGAGTCCGTCAACGCCGGCAACCACAAGTTCTGGCTGGAGGCGAACCGTTTCGCCGACCTCACCGACGACGAGTTCAGGGCCACCTGGACGGGTTACAGGCCCAAGACTGCGGCGGCCAGCAGCAAGGGACGGAGCCGGACGGCGACGACAGGGTTCAAGTACGCCAACGTCAGCCTCGACGACGTTCCGGCGTCCGTGGACTGGAGGACCAAAGGCGCCGTCACGCCCATCAAGAACCAAGGTGAGTGCGGTTGCTGCTGGGCGTTCTCCGCGGTGGCGTCCATGGAGGGCGTCGTGAAGCTGAGCACGGGGAAGCTTGTCTCCCTCTCGGAGCAGGAGCTAGTGGACTGCGACGTGAACGGCATGGACCAGGGCTGCGAGGGCGGCGAGATG

GACGACGCCTTCGACTTCATCGTGGGCAACGGCGGCCTCACCACCGAGAGCAGGTACCCGTACACCGCCTCCGACGGCACCTGCAACTCCAACGAGGCCTCCGGCGACGCGGCGTCCATCAAGGGCTACGAGGACGTGCCGGCCAACGACGAGGCCTCCCTGCGCAAGGCCGTCGCCAACCAGCCCGTGTCGGTCGCCGTCGACGGAGGGGACTCCCACTTCAGGTTCTACAAGGGCGGCGTCCTGTCCGGCGCGTGCGGCACGGAGCTCGACCACGGGATCGCCGCCGTCGGGTATGGCGTCGCCAGCGACGGCACCAAGTATTGGGTCATGAAGAACTCGTGGGGCACCTCTTGGGGGGAGGCCGGGTACATCCGGATGGAGAGGGACATCGCCGACGAGGAGGGTCTGTGCGGCCTCGCCATGCAGCCCTCCTACCCGACAGCCTAGCCGTTGATGAAGAACGTACCCAATTAAACTGCTAGATATTAGTCCATCTATAAGATTGTGTGTAATTAAGGTTGTGAGGGCGAGACGACATGAAGCTTCACCTGCACTGTAAATAATTAA

>ZmCP17

ATGGTCAGCTCCAAGGCTTTCCTCCTCCTCCTTGCCGTCCTTATTGGTTGCGTCTGCAGCTTCCCTAGTCCTGTCCTAGCGGCTCGTGAGCTGAGCGACGATGCAGCCATGGCCGAGAGGCATGAGAGATGGATGGCGGAGTATGGCCGTGTCTACAAGGACGCCGCCGATAAGGCACGGCGCTTCGAGGTGTTCAAGGATAACTTCGCATTCGTCGAGTCGTTCAACGCCGATAAGAAGAACAAGTTCTGGCTTGGCGTCAACCAGTTCGCCGACCTGACTACCGAAGCGTTCAAAGCAAACAAAGGCTTCAAACCGATCTCAGCAGAAAAGGCCCCAACGACTGGATTCAAGTACGAGAACTTAAGCATTAGCGCGCTTCCGACGGCCGTCGACTGGAGGACCAAGGGAGCTGTCACACCCATCAAGAACCAAGGCCAGTGTGGTATGTGCATTCATATTTTATGGTCATACTTGAAAGACATACTTCTTATGATGACATGAACAGTTAATGACACCAATAACTGCATATGATGACATGAACAGTTAATGACACCAATAACTGCAGGTTGCTGCTGGGCGTTCTCGGCCGTAGCTGCCGTGGAGGGCATCGTGAAGCTGAGCACCGGCAACCTCGTCTCACTCTCAGAGCAAGAACTAGTAGACTGTGACACCCACAGCATGGACGAGGGTTGCGAGGGGGGCTGGATGGATAGCGCCTTCGAGTTCGTCATCAAGAACGGAGGCCTGGCCACCGAGTCCAGCTACCCGTACAAGGCCGTGGACGGCAAGTGCAAGGGCGGGTCAAAGAGCGCCGCGACGATCAAAGGCCATGAGGACGTGCCGCCCAACAACGAGGCTGCGCTCATGAAGGCCGTGGCCAGTCAGCCTGTGTCGGTCGCCGTCGATGCAAGTGATAGGACTTTCATGCTTTACTCCGGCGGTGTGATGACCGGCTCCTGCGGCACTCAGCTGGACCATGGCATCGCTGCGATCGGCTACGGCGTGGAGAGTGATGGCACAAAGTATTGGATTATGAAGAACTCGTGGGGCACCACTTGGGGAGAGAAGGGGTTCCTACGGATGGAGAAGGACATTTCTGACAAGCGAGGGATGTGCGGCCTTGCCATGAAGCCTTCCTACCCCACCGAGTAG

>ZmCP18

ATGGTGAGCTCCAGGGCTTTCCTCCTCCTCCTTGCCATCCTTACTGGCTGCGCCTGCAGCTTCCCTAGTCCTGTCCTGGCGGCTCGTGAGCTGAGCGACGATGCAGCCATGGCCGAGAGGCATGAGAGGTGGATGGCGGTGTATGGCCGCGTCTACAAGGATGCAGCCGAGAAGGCACGACGCTTCGAGGTGTTCAAGGACAATCTCGCATTCGTCGAGTCGTTCAACGCCGATAAGAAGAACAAGTTCTGGCTTGGCGTGAACCAGTTCGCCGACCTGCTACCGAAGAGTTCAAAGCAAACAAAGGCTTCAAACCGATCTCAGCAGAAGAGGTCCCAACGACTGGATTCAAGTACGAGAACTTAAGCGTTAGCGCGCTTCCGACGGCCGTCGACTGGAGGACCAAGGGAGCTGTCACACCCATCAAGAACCAAGGCCAGTGTGGTATGTGCATTCATATTTTATGGTCATACTTGAAAGCCATACTTCTTATGATGACATGAACAGTAAATGACACCAATATATAACTGCAGGTTGCTGCTGGGCGTTCTCGGCCGTAGCTGCCATGGAGGGCATCGTGAAGCTGAGCACCGACAACCTCGTCTCACTCTCAGAGCAAGAACTGGTGGACTGTGACACCCACAGCATGGACGAGGGTTGCGAGGGGGGCTGGATGGACAGCGCCTTCGAGTTCGTCATCAAGAACGGAGGCCTCGCCACCGAGTCTAGCTACCCGTACAAGGCCGTAGACGGCAAGTGCAAGGGCGGGTCAAAGAGCGCCGCGACGATCAAAGGCCATGAGGACGTGCCGCCCAACAACGAGGCCGCGCTCATGAAGGCCGTGGCCAGTCAGCCTGTGTCGGTCGCCGTCGATGCAAGCGACAGGACTTTCATGCTTTACTCCGGCGGTGTGATGACCGGCTCCTGCGGCACTCAGCTGGACCATGGGATCGCTGCGATCGGCTACGGCGTGGAGAGTGACGGCACAAAGTATTGGATTCTGAAGAACTCATGGGGCACCACTTGGGGAGAGAAGAGATTCCTACGGATGGAGAAGGACATTTCTGACAAGCAAGGGATGTGCGGCCTTGCCATGAAGCCTTCCTACCCCACCGAGTAG

>ZmCP19

GCGGCTCGTGAGCTGAGCAACGATGCAGCCATGGCCGAGAGGCATGAGAGGTGGATGGCGGAGTATGACCGCGTCTACAAGGACGCAGCCGAGAAGGCACGGCGCTTCGAGGTGTTCAAGGATAACTTCGCATTCGTCGAGTCGTTCAACGCCGATAAGAAGAACAAGTTCTGGCTTGGCGTGAACCAGTTCGCCGACCTGACTACCGAAGAGTTCAAAGCAAACAAAGGCTTCAAACCGATCTCAGCAGAAGAGGTCCCCACGACTGGATTCAAGTACGAGAACTTAAGCGTTAGCGCGCTTCCCACGGCCGTCGATTGGAGGACCAAGGGAGCTGTCACACCCATCAAGAACCAAGGCCAGTGTGGTATGTGCATTCATATTTTATGGTCATACTTGAAAGCCATACTTCTTATAATGACATGAACAGTTAATGACACCAATATATAACCGCAGGTTGCTGCTGGGCGTTCTCGGCCATAGCTGCCATGGAGGGCATCGTGAAGCTGAGCACCGGCAACCTCGTCTCACTCTCAGAGCAAGAACCGGTGGACTGTGACACCCACAACATGGACGAGGGCTGCGAGGGGGGCTGGATGGACAATGCCTTCGAGTTCGTCATCAAGAACGGAGGCCTGGCCACCGAGTCCAGCTACCCGTACAAGGTCGTGGACGGCAAGTGCAAGGGCGGGTCAAAGAGCGCCGCGACGATCAAAGGCCATGAGGACGTGCCGCCCAACAACGAGGCCGCGCTCATGAAGGTCGTGGCCAGTCAGCCTGTGTCGGTCGCCGTCGATGCAAGCGACAGGACTTTCATGCTTTACTCCGGCGGTGTGATGACCGGCTCCTGCGGCACTCAACTAGACCATGGCATCGCTGCGATCGGCTATGGCGTGGAGAGTGACGACACAAAGTATTGGATTCTGAAGAACTCGTGGGGCACCACCTGGGGAGAGAAGGGGTTCCTACGGATGGAGAAGGACATTTCTGACAAGCGAGGGATGTGCGACCTTGCCATGAAACCTTCCTACCCCACCGAATAG

>ZmCP20

CCATCAAACCAAAGCAGAGTCGTCAACAACAGCAGGAAACATAACTGAAAGAGCTAGCACTACCTGATCGAGCACCATGGGCATCCCCAAGGCTTTGCTTCTTGCCATCCTCGGCTGCGGCGTCTGCTTGTGCAGCGCTGCTGTCCTTGCAGCTCGCGAGCTGGGGGGCGACGACGAGCTGGCCATGGTGGCGAGGCACGAGCAGTGGATGGTCCAGCACGGGCGCGTCTACAAGGATGAAACCGACAAGGCACACCGGTTCCTAGTGTTCAAGGCCAACGTCAAGTTCATCGAGTCGTTCAACGCTGCCGCCGCCGCCGGCAACCGCAAGTTCTGGCTGGGTGTGAACCAGTTCGCCGACCTCACCAACGACGAATTCAGAGCAACCAAGACCAACAAGGGATTTAACCCAAATGTGGTGAAGGTTCCCACGGGATTCCGGTACCAGAATCTTAGCATCGATGCACTTCCGCAAACCGTGGACTGGAGGACCAAGGGTGCTGTCACGCCCATCAAGGACCAAGGCCAGTGCGGTAAGTAAACTAAAATTCTCCTACATATGTATATGGTTTATTATTTATTTATTTATTAGAACAAAGGTTGACAATGGCATGCATCTGAAAAACTGAACAAAACGAATGTGCAATGCAGGCTGCTGTTGGGCGTTCTCAGCGGTGGCTGCCACGGAGGGCATCGTCAAGATAAGCACCGGCAAGCTAACCTCACTCTCGGAGCAAGAACTAGTGGACTGTGACGTCCACGGTGAGGACCAGGGCTGCAACGGCGGCGAGATGGACGACGCCTTCAAGTTCATCATCAAGAACGGTGGCCTTACCACCGAGTCGAACTACCCTTACACCGCGCAAGACGGCCAGTGCAAGTCCGGATCCAACGGCGCCGCAACCATCAAGGGGTACGAGGATGTGCCTGCCAACGACGAGGCCGCCCTCATGAAGGCGGTAGCTAGCCAGCCAGTCTCTGTGGCGGTGGACGGCGGCGACATGACATTTCAGTTCTACTCTGGAGGCGTGATGACGGGATCTTGCGGCACTGACCTCGACCATGGTATTGCAGCTATTGGTTATGGAAAGACCAGTGATGGCACCAAGTACTGGCTGATGAAGAACTCGTGGGGCACAACTTGGGGAGAGAATGGATTTCTGAGAATGGAGAAGGATATTGCTGACAAGAAGGGGATGTGTGGCCTTGCCATGCAACCATCCTACCCTACTGCGTAGGCTAAGCTTCGAATGATATCGCTTCCGTACATTAATACTTGTGGTCTGTATATTGTGTCATATTGTGTCATATGAATATATATGTGTTCTGTA

>ZmCP21

CTACTGCGTGTGCTGTCAATACACTTCTTTTTTTTTCTTCCAATTTTTATCTGATACTATATGTTACTGTATGCATGTAATAATCAAATGTGGCGCTTATAATCTTGATTTTTATAATGTTCAAGAGAGTCCACAGCAGAATGGCAAATGCACGTCTCTGGCACAAGTATTTTAATATACCACACATACTTCACGGCCACGGACCTGTCCCATTGGCCGTTGCATACAGTAAAGTAAAGCGTAGGAGCACCGTATGGCTTCGTACTTAACAATAATACAAATCTGTAGCTTCTTATATATATATAGACGCATACCACCAAGCTCTAATCACCAAATCAAAAGGCATAGTGCACAGCATCAGCAGCAACAGCCAATTGACAGGCCTGATCGATCGAGTAGCGTGATCGACCATGGCCACCCTCCAGGCATCGATCTTGGCAGTCCTCAGCTTTGCCTTCTTCTGCGGTGCTGCTCTTGCTGCTCGCGACCTGAACGAGGACTCAGCCATGGTGGCCAGGCATGAGCAGTGGATGGCGCAATACAGCCGCGTCTACAAAGATGCCGCCGAGAAGGCTCGACGGTTCGAGGTGTTCAAAGCTAATGTTAAGTTCATCGAATCATTCAATACAGGTGGGAACCGTAAGTTCTGGCTTGGTATCAACCAGTTCGCTGACCTCACCAATGATGAGTTCAGGACTACCAAGACCAACAAAGGCTTCAAACCTAGCCTCGATAAGGTCTCTACCGGATTTAGGTATGAGAATGTTAGCGTTGATGCGATTCCAGCGACTATCGACTGGAGGACCAATGGTGCAGTCACTCCGATCAAGGATCAAGGGCAATGTGGTAAATACATTTGGTCCTAATAAGACTCATAAAAACACTATTATGATGTAAACTAAAGTGCCATAACATATGTCAAAACATATATATAATGATCGATGATTGCAGGTTGCTGCTGGGCATTCTCGGCCGTGGCAGCCACAGAAGGTATCGTGAAAATTAGCACTGGCAAGCTCATCTCCCTCTCAGAACAAGAATTGGTGGATTGCGATGTCCATGGTGAGGATCAGGGTTGTGAGGGTGGCCTGATGGACGATGCATTCAAGTTTATCATCAAGAATGGAGGCCTAACAACGGAGTCCAACTATCCATACACGGCTGCAGATGGCAAGTGCAAGAGTGGATCGAATAGTGCTGCAAACATCAAGGGCTACGAGGATGTGCCAACAAACGATGAGGCTGCCCTGATGAAGGCCGTGGCAAACCAGCCCGTGTCGGTGGCAGTGGACGGTGGAGACATGACATTTCAGTTCTACTCTGGTGGTGTGATGACTGGATCATGTGGTACTGACTTGGACCATGGGATTGCAGCCATTGGTTATGGGAAGACTAGTGATGGAACCAAGTATTGGCTCATGAAGAACTCATGGGGCACGACATGGGGTGAGAATGGTTACCTGAGAATGGAGAAGGATATTTCAGACAAGAAAGGCATGTGCGGACTAGCCATGGAACCTTCCTATCCCACTGAGTAGGCAAAACCTTCAGAAGCCTTTTAGTTCATACATGAACACATAGACAGAATAAATCGGTGCTTCTATACATTCTACTACATATATAAAGCTTGTCCATA

>ZmCP22

ATGGCCACCCTGAAGGCATCGATCTCGGCCATCATTGGCTTCGCCTTCTTCTGTGGTGCTGCCATGGCTGCTCGCGACCTGAGCGACGACTCTGTCATGGTGGCGAGGCATGAGCAGTGGATGGCGCAGTACAGCCGCGTCTACAAAGACGCCTCCGAGAAGGCTCGGCGGTTCGAGGTGTTCAAGGCTAACGTTCAGTTCATCGAGTCGTTCAACGCTGGTGGGAATAATAAGTTCTGGCTCGGGGTTAACCAGTTCGCCGATCTCACCAACGACGAGTTCAGGTCTACCAAGACTAATAAGGGTCTCAAATCTAGCAATATGAAGATCCCTACAGGATTTAGGTATGAGAATGTTAGTGCCGATGCGCTTCCGACGACCATCGATTGGAGGACCAAGGGTGCCGTCACTCCCATCAAGGATCAAGGTCAATGTGGTATGTATATTTAGTAGCACGAACACTTCCGACAACTCTATTATGGTGGAAATAAAAACTAGAGTGTGATGACATAAGTCGAGACATAATAATCAATTGCAGGTTGCTGCTGGGCGTTCTCAGCCGTGGCCGCCACAGAAGGCATTGTGAAAATCAGCACGGGCAAGCTCGTCTCTCTTGCAGAACAAGAGTTAGTGGATTGCGATGTCCATGGCGAGGATCAAGGTTGTGAGGGTGGTTTGATGGACGATGCTTTCAAGTTCATCATCAAGAACGGCGGCCTAACCACGGAGTCCAGCTATCCTTACACTGCTGCAGATGGCAAGTGCAAGAGTGGATCAAATAGTGCTGCAACCATAAAGGGCTACGAGGATGTGCCGGCAAATGATGAGGCTGCCTTGATGAAGGCCGTGGCAAACCAGCCTGTCTCGGTGGCAGTGGATGGCGGAGACATGACGTTCCAGTTCTACTCTGGTGGTGTGATGACTGGCTCATGTGGCACTGACTTGGACCATGGGATTGCAGCCATTGGTTATGGAAAGACCAGTGATGGTACTAAGTACTGGCTGATGAAGAACTCATGGGGCACGACTTGGGGCGAGAATGGATACCTCAGAATGGAGAAGGATATTTCAGATAAGAGGGGCATGTGTGGTCTAGCCATGGAACCTTCGTACCCCACTGAGTAG

>ZmCP23

ATGGCCACCCTCAAGGCATCGATCTTGGCCATCCTTGGTTTCGCCTTCTTCTGCGGTGCTGCCTTGGCTGCCCGCGACCTGAGCGACGACTCTGCCATGGTGGCGAGGCATGAGCAGTGGATGGCGCAGTACAGCCGCGTCTACAAAGACGCCTCCGAGAAGGCTCGGCGGTTCGAGGTGTTCAAGGCTAACGTTAAGTTCATCGAGTCGTTCAACGCTGGTGGGAATAATAAGTTCTGGCTCGGTGTCAACCAGTTCGCCGATCTCACCAACGACGAGTTCAGGTCTATCAAAACTAATAAGGGCTTCAAATCTAGCAACATGAAGATCCCTACAGGATTTAGGTATGAGAATGTTAGCGTCGATGCGCTTCCGACGACCATCGATTGGAGGACCAAGGGCGCCGTCACTCCCATCAAGGATCAAGGTCAATGTGGTATGTAATTTAGTAGCACGAACACTTGTGAAAACTCTATTCCGATGGAAATAATTAACTAGAGTGTGATGACATAATTCGAGACATAATAATCAATTGCAGGTTGCTGCTGGGCGTTCTCAGCCGTGGCTGCCACCGAAGGCATCGTGAAAATCAGCACCGGTAAGCTCGTCTCTCTTGCAGAACAAGAGTTAGTGGATTGCGATGTCCATGGCGAGGATCAAGGTTGTGAGGGTGGTTTGATGGACGATGCTTTCAAGTTCATCATCAATAACGGTGGCCTAACCACGGAGTCCAGCTATCCTTACACTGCTGCAGATGGCAAGTGTAAGAGTGGATCAAATAGTGCCGCAACCATAAAGGGCTACGAGGATGTGCCAGCAAATGATGAGGCTGCCTTGATGAAGGCCGTGGCAAACCAGCCTGTGTCGGTGGCAGTGGATGGTGGAGACATGACGTTCCAATTCTACTCTAGTGGTGTGATGACTGGCTCATGCGGTACTGACTTAGATCATGGGATTGCAGCCATTGGTTATGGAAAGACCAGTGATGGTACTAAGTACTGGCTGATGAAGAATTCATGGGGCACGACTTGGGGCGAGAATGGATATCTCAGAATGGAGAAGGATATTTCGGACAAGAGGGGCATGTGTGGCCTAGCCATGGAGCCTTCCTACCCCACCGAGTAG

>ZmCP24

ACCACCACCATAGATCGATCCTTGAGTTGACGAAAACATACACAGCTGCTAGCTAATCCTGCCGTGAGTGCAATGCATGCGAAGATGACGAAGCCCGCAGCCATCGTTGTTGCTGCTATCGCCGTCCTCTCCGTCAGCCTGCTGGCTGGGTCGTCGTGCCTTGCCCTTGCGCGGCCCAGCGGCGACTTCTCCATCGTGGGCTACTCAGAGGAGGACCTGTCGTCGCACGAGAGCCTGGCGGAGCTGTTCGAGCGCTGGCTGTCCCGCCACCGCAGGGCGTACGCCAGCCTGGAGGAGAAGCTCCGGCGGTTCCAGGTGTTCAAGGACAACCTGCACCACATCGACGAGACCAACCGCAAGGTGAGCAGCTACTGGCTGGGCCTCAACGAGTTCGCCGACCTCACGCACGACGAGTTCAAGGCCACCTACCTCGGCCTACGGAGCAGTGTTGGCGATGGTGGTAGTGGTATCGATGACGACGACGAGCCGGAGGAGGAGGAGGGCTACGAGGGTGTGGACGGGGCGAGCCTCCCCAAGTCGGTGGACTGGCGGAGCAAGGGCGCGGTGACGGGGGTAAAGAACCAGGGCCAGTGCGGCAGCTGCTGGGCCTTCTCCACGGTGGCCGCCGTCGAGGGCATCAACCAGATCGTCACGGGCAACCTCACGGCGCTGTCGGAGCAGGAGCTCATCGACTGCGACACGGACGGCAACAACGGCTGCAACGGCGGCCTCATGGACTACGCCTTCTCCTACATCGCACACAACGGCGGCCTCCACACGGAGGAGGCCTACCCGTACCTCATGGAGGAAGGCACCTGCCAGCGCAGCAGCAGCAGCGAGAAGAAGTGGCCGGGATCGTCGGAGGACGCCAACGACGATGCCGCGGTGGTCACCATCTCCGGGTACGAGGACGTGCCGAGGAACAACGAGCAGGCGCTGCTCAAAGCGCTGGCGCAACAGCCCGTCAGCGTCGCCATCGAGGCCTCCGGCAGGAACTTCCAGTTCTACAGCGGCGGCGTCTTCGATGGGCCCTGCGGCACGCAGCTGGACCACGGGGTAGCGGCCGTCGGCTACGGGACGGCGGCCAAGGGCCATGACTACATCATCGTCAAGAACTCGTGGGGGCCCAGCTGGGGCGAGAAGGGGTACATCCGCATGAGGAGGGGCACCGGCAAGCGCCAGGGCCTCTGCGGCATCAACAAGATGGCCTCCTACCCAACCAAGAACTGAACTCAGATCATCGATTCATCGTCAAGCAGCAGCTGGCTGATTACTCCCACTGCTGGCGCTCCACGCTTCACTAGTTCTTCGTCGTTCTCCTTCACTTCCATTGTTTTCAAGATTGGACGATCTTTCGTTGTTTGAAATGAAATAATAATGATGATGGAATTAGTGTTCCCCAAGTTCGAACAAATTAAGTATTGTTGGAGTTTTCGTTGGAACAACTGTAGTCTGTAGTCAGAGTGAGCCCCAAATCAGGGCAGTTGCTTTTGAACATAACATT

>ZmCP25

ATCCATCCCGAGCAAGCAAGTAATATGGCTTGGTCTTGTGCTCGTCCGATGAGCATCGCCCTCGCTGCCGTCCTCCTGCTGTGCGGCGGCGCGTGGCTGCAGCAGGCGGCGGAGGCGCGCCCCCACCACATGGACGACGACAGCAGCATCGACATGGACCGCGGCAGCGACGACTTCTTCTCCATCGTGGGGTACTCGCCGGAGGACCTGACGCAGCACGACCGGCTCGTCAGGCTGTTCGAGGAGTGGGTGGCCAAGTACCGCAAGGCGTACGGCAGCTTCGAGGAGAAGCTGCGCCGGTTCGAGGTGTTCAAGGACAACCTGCACCACATCGACGAGGCCAACAGGAAGGAGGTCACCAGCTACTGGCTGGGCCTCAACGCCTTCGCCGACCTCACGCACGACGAGTTCAAGGCCACCTACCTGGGCCTCCTGCCGAAGAGGACCAGCGGTGGCCGGTTCAGGTACGGCGGCGTGGGCGACGGCGGCGACGAGGTGCCCGCGTCCGTGGACTGGCGGAAGAAGGGCGCCGTGACGGAGGTCAAGAACCAGGGGCAGTGCGGCAGCTGCTGGGCCTTCTCCACGGTGGCGGCCGTGGAAGGGATCAACCAGATCGTGACGGGCAACCTCACGTCGCTGTCGGAGCAGCAGCTGGTCGACTGCAGCACCGACGGGAACAACGGGTGCAGCGGCGGCGTCATGGACAACGCCTTCTCGTTCATCGCCACCGGCGCCGGGCTCCGCTCCGAGGAGGCGTACCCGTACCTCATGGAGGAAGGGGACTGCGACGACAGGGCCCGCGACGGCGAGGTGCTCGTCACTATATCCGGGTACGAGGACGTGCCGGCCAACGACGAGCAGGCGCTCGTCAAGGCCCTCGCCCACCAGCCCGTCAGCGTCGCCATCGAGGCGTCCGGGAGGCACTTCCAGTTCTACAGCGGGGTACGTACGGCAAACTCTGATTGCATTTGCAGCTGGACTAGACTGGGCCATTGATGATCTCTGATTGCACGATGCATGCAGGGCGTGTTCGATGGACCGTGCGGGTCGGAGCTGGACCACGGCGTGGCGGCGGTGGGCTACGGCAGCAGCAAGGGGCAGGACTACATCATCGTCAAGAACTCGTGGGGCACCCACTGGGGCGAGAAGGGCTACATCCGCATGAAGAGGGGCACCGGCAAGCCGGAGGGCCTCTGCGGCATCAACAAGATGGCATCCTACCCGACCAAGGACCATTGATGGATCATGGACGACGGAGATCATATCTATCTTCCCTTCCCTTTCATCCGACTCTTTCGATTTCCATCGTTTGCCCCCTTTTCCTGAATGTGTGTGTTTTTTTTTATATATTACTGTGTCAAGTAATTTCTCGATGGATCGATCGAGGAAGAACGACAAACATGCACTCAAGCACGAGCGCAGCAATGTCATCTTTCTCTTCATTTGGTGCGTTGAAATGACGAATATCAAATAAAGTGGTTTATTT

>ZmCP26

ACCCTTACTATAATTCATCATACTACCAAAACCAAGATTTAAGCGTCATCTGAGGCATTGGAGAAGCACCGAAAAAGCATCCTTGGACGAAACTTGTGGAACAGAAGCATACGTGCAACTGCAAGGCCGCAGCAACAGCTTCCTGCTAAAGCAAGGCTCTGCCCTTGAGAAGCTTCATCCAGAAGAGCCATAGCTGGTGCAGGGAATTTACTTGCAAGAAACATAGATATTCGCAGTATAAAGGTGTTCCTGGATCCTGCACATGCCATGACAACAAGGGGGCAAAAAATAGCAGCAGGCATTTGACATGGAACCCAAACTCGCGGTCGCGGTGTTCGTCCTGTTCCTAGCGTTTGCAGCCTGTTCCGCGAACCACCACCGAGACCCCTCTGTCGTCGGATACTCCCAGGAAGACCTCGCACTGCCCAGCAGCCTCTTCAGATCCTGGTCCGTGAAGCACGGCAAGCTCTACGCCAGCCCGACGGAGAAGCTGGAGAGGTACGAGATATTCAAGCAGAACCTGATGCACATCGCGGAAACGAACAGGAAGAACGGGAGCTACTGGCTGGGCCTGAACCAGTTCGCCGACGTCGCCCACGAGGAGTTCAAGGCCAGTTATCTGGGACTGAAGCGAGCACTGCCAAGAGCGGGCGCGCCACAGACACGCACCCCGACAGCGTTCAGGTACGCGGCCGCAGCCGCAGGCAGCTTGCCCTGGTCAGTCGACTGGAGGTACAAGGGAGCCGTGACGCCGGTCAAGAACCAGGGAAAATGCGGTGAGTTCCGTCTCTGGACAATACACGAACGCCATCCTCTTCTCAGCGTGTTGCTGTGCCGATTAGATGCCACTAGACGGTGGGAGATGCGATTACAAGGCGAACAAAAAAAAAATGCTTGCAGGGAGCTGCTGGGCCTTCTCGTCGGTGGCAGCAGTCGAAGGGATCAACCAGATCGTGACGGGCAAGCTGGTGTCGCTCTCGGAGCAGGAGCTGGTGGACTGCGACACCACGCTGGATCACGGCTGCGAAGGGGGGACCATGGACTTGGCGTTCGCTTACATGATGGGGAGCCAGGGGATCCACGCCGAGGACGACTACCCGTACCTCATGGAAGAAGGCTACTGCAAAGAGAAACAGGTTCGCACACAAAAATCTTCACATGTGAACTGGATGATACTAGGACGCAGGGTTCTAATCCTTTGCTCTGGCGTGCGTGTTTAGCCCCAAGCTGACGTCGTCACTATAAGGGCTTGTTCGGTTAGCTCTCAATCCATGTGGATTGAGTGGGATTGGATGGGTTTAAATTCCAAACAAGTCAAACTTCTTAAGAATTTTTTCCAATCCCATCCAATCCATGTGTATTGGGAATAACCGAACAAGACCTAACTGGATTCGAGGATGTCCCGGAGAACAGCGAGATCAGCCTGCTGAAAGCGCTGGCCCATCAGCCTGTCAGCGTCGGCATAGCTGCAGGGAGCAGGGACTTCCAGTTCTACAGAGGGGTAAGTATCAACTGACTTGCCTGTAGTATGGTTGGTTTCAGATGGTGTCATCACTAGCTGTCACAGAAGGGAAGTAACATGTGCGTGTATACCTCGTCTGGCTGGCTGCACTGCTGCAGGGGGTGTTTGACGGGGCCTGCAGCGTCGAGCTCGACCATGCGCTGACGGCCGTCGGGTACGGCTCATCGTACGGCCAGAACTACATCACCATGAAGAACTCGTGGGGCAAGAACTGGGGGGAGCAAGGCTACGTCAGGATAAAGATGGGCACCGGGAAGCCGGAGGGTGTCTGCGGAATCTATACCATGGCCTCCTATCCCGTGAAAAATGCAACACGCTGGGGTGCTTAATATTTTTGTAACGAGTTTGTTCGAAACTATTCTTTTAAATATATCCACATGGCAAAATGCCTTTTTTTTTA

>ZmCP27

CCATCTTCGCTTCTCTTCTCCACATTTCTGCGCTATCCCGGCGTACGGCTGCGATCCATACACGCCCAATCCAAACCCTACCTAAAACTCCGCCTCGTGCCTCCGGGCTCACACTCGTAGGCACGGAGTGCCGTTCCGTGGTGGGTGGAATCACCAAAAATGCCCTCCGTCCACCACCACCTCCTCCTCCTGACGCTCGCCGCCTTGGCCGTGGCGGCAGCCACCGCTAGCGCCGGCGGGGACCCGCCGGCAATCGAGGCGCAGTTCGACGCCTGGTGCGCGGAGCACGGGAAGGCCTACGCCACGCCCGAGGAGCGCGCCGCGCGGCTGGCCGTGTTCGCGGACAACGCCGCCTTCGTCGCCGCGCACAACGCCCGTGCAGGCGCCAACGCCGCTGGCGGCGGCGGCGGCGGCGCCGCGCCCCCGTCCTACACGCTCGCGCTCAACGCCTTCGCGGACCTCACGCACGAGGAGTTCCGCGCCGCGCGCCTCGGCCGCATCGCGCCGGGGGCGGCGCTCCGGAGCCGCGCCGCGCCCGTGTACTGGGGCCTCGGCGGCGGTGCCGCGGTGCCTGATGCGCTGGACTGGAGGAAGAGCGGGGCCGTCACCAAGGTCAAGGACCAAGGCAGCTGCGGTGAGCGTCCCTTACTCGATCTGCTCTATGTTTTGCTTAGGTTGAAATTTGCAAAACCCATCTGTCTCCCACATCAGAGTCCACTCCAGTGTAGTGTTCAGATTCATTGTTAGGGACCCTGTTGACAACTCTATGGTTAGCACATCTGAACGAGTGAACGAGGTACACAGTAGTGGGTTAATATCCACTTGACCGTTCGTATAAGGAGTAGATTCGACTCGTGGATTAAGCCTAACAGTAAAGGGAACCGAGGCTGAGGAATTCAAGCCCATTTTGGAACTTACGATTTCACCAGGTGCGCCGAAACATGGAGGATTTATCAATCGACAACAATAGCTCACTTCATTTTCTATGGACTAAATTACACCTATGGTGTTTCTCATCCTATCCTATTAATGTTTTGTTTACATGAACATTCGCTGTTGTACTGACTAGAAGTTTCAAACTGTATGATCTCAGTCATTTGAAGCTTTTTAGCTCTTTCCAGGCACTAGATCTAGATCCAATCCAAACGACACTGAGTTTTGCTATAACAAAGTTAGTAAATGACTACCACCTGTTCTTTAGATTGGGTCATTTAAATGATCTGGTGACAACTAGATAAGTCAGACATCAAAAGTCACATATGAATCAAAAATAGTTTCACAGTCCATGTGAGATTCTGTATTGCGTTCAATGAGATGTCAGCTACATGTTTGTTTCTGTGTACGATCTGTTGCACTAAAGATTTGCATACGGAGTATACAGATATAATTTTAGTTTACTGTTGGAAAAGACTTACATGTCTTGAATATATTATGTAACGCACTTGAGCACTATGAAGTTCATCAGAGCAATTCCAGCAGGGCCCCTACTTAAGCATAGCTAAATATGGGTATCCAGACTAAAAACACAGCGCGAGCTGGACCCCTACTAGAGCACCCAAATCCACCCCAGGGACCCCATTCTAGGGGCTCAGGTGCAGGCACCTGCCTGTACGCTGAATCAGCATGGTGGCTCTTCCCCATCTCTCTCTTCAACTCTCTCCGGCAGATCCCGTAGCGGTGGAGGGCTTGTGTGGCCTCTCCGGCAGGCGGCAGCCCCTCCCCTCCGGCTACCTCCTGTTAGAGGAATAGACATTTTACGGTACATTTTAATTCCAAAATTCTGTAGATAAATCATGAAGATGGATATTCTGAATGAAGCAGTGACGTCATAATCTGTGCATGTGTTCATGTTTAACATTGTTTTAATCATGAATAGTATAAACTATAAAACAAGAAGTTTCAGAAAGTACATTGAAGCGGAGCCTTTGCCGGAAGCATTGGCTCCGCTCTTGCCAACTGTAGTAGTCATACTACTCGATTGGCACCAGTTGTAGCCATACAGTTTGATGCAGTGAAGAGCTGATGCCGTGCAGTCCCTTGAATGATTGCTAGAAAGTAGGTGATATAGTTGTTCAGTGAGTACTCACATGTAGGTGCATGCTCTTCAAAAACCTTACTGGCTGCTATCCCAAGCAGGATTTTCAACGACCAAGGCTTTGGAGGCCTACTCTTGTCAGGTCCGTACGCGTGGAGCCGTTGCAATGGAGGTGCTCTTCTCAGCAATAGCAGGAAGCTGAGGGAGAAGGAAGATGTCTCTTAAGTAGAAGTACCTGAGCTGTGAGGGTGTGTTCTAAGGAGGGGTGGCGCCTTCTTATATATGTGAGGAAGGACTGAGCAGTGGAAGAACCAGGACGCCATTTATACAATGAATAGGACGCTATTGATGGGGTCAAATTCTGGTAGTTACTGTAACGACCAAATGCCCAGGCCATCATGGTCATCGTTGTTATCAGTTACTGCAAAAACTGATCTCATCATAGCACAACACATCACGTCCGGCCCAACCGTGCCGCCTTCTCGTCTTGTCTCGTCATGACACACCCTCAGTCTCATCCTTGGCCTGGCTCGACGAGACGAGTGAGCAAAAGATTCTGCCATGCTGGTCTCCTTTTTCAACTTCACAGATGGTGTACATAGGGTCCACCTTATAAGTTGGTTAGGATCCCTCGTACTTCCAAGGTGGGATTAATCACCTTTGACTCTCTAGTATTATATGAGCCTTTAATATTAGATGATTTAATGGATTAATTTGGGCTAAGCCCATTAAATCCAACAATCCCCACCAAATAATTCAAAGCATATCAAGAACAATGATTTCAAGTCGCGTGGTCGCCTAGTCGATCCCTGGGACCGACCAGGCGACTAGTCGCGATTAGGCGACGACTAGGGCGATTAGTCGACTCCTAGTCGTTCCCTGGTCGTCCTAGTCTAGTAGTTATACTTATATAGCTATATATATAAATGTACATGTATACAACTACATTACATAAATTAGAAAGTCACTACTTAGCACAGGCTTAGGATTAGGGCAGTCAGCAACCATCCACTAATTTGGCAGAACTGCAGAGAAGGGGGAGATGGGGGGAGAGCAGGAGGAACCTTGAGATGACGAGCTCATCTCCTCAATGGTGCAGGTCTGCTGGAGCTTGGGAAGGGGCAGGGTGGAGACTACAGTTGGTGAACCTTAAGACAGTCGCCGCATGTGGAGACTGTAGCAGGCGAGCATGAGAAGGGGCAGGGTGGTGTAGCCGCTGATGATGTAGCCGTCGGTGAGCTGTAGGTCTGAAGGGGCAGCCACCGACGACCAAGTGTGAATAAGGGCTGGAACAGTCGAGCTAGCAAGAGAGGAGAGTAGGAGACGTAGTGCACTGTTTGACGGCTAGGTATTACATTGGAGAGGAGAGGGTAGTCTAAAGTATACTGTAGATGGGTTGAAAATGGCCCAATAACAAAAACCTAGCCAGGACGACCAGGAAACAGTAATCGGGCGATTAGTCGGACGACCAGGACGACCAGCCTATCTAGTTGAGTCGTCCAGCTAATCGAGCCCAAATAGCCGACCAGCCCGACTAATCGCGACTAATCGCGATTAGTCGGACGACTTGAAATCATTGATCAAGAATGCTCTCAAGCGTAATGCTATTTTGATATACTAGTGTTTAGATGGAGAGTTGGAGACTGTTAAGTTGAACATCTACCTAGAACTGAGACTACACTTGTTTACAAGTGTACAATGGACGATGCCTTGAACTGACAGTTTTGTGCAATCAAGATTGGCCAAAACACTTAAGGATATCTCCAGCAGCTTACCCACCCCCATACCCATATTCAAACTCCACTCTACGAACAGTGCAGTTTACAGTGCAAAACAATGTTTTGAATGACCATATGGGTGAACTGGTGAACGCGGTCTAACTAGCACTAGGAGGCAAAGGCATCCTCTCGGTTTGGAGCCTATAAGTATGGTCGTACTCTATGCCCTTTCATGAGTGTTTAGAGGTTACCTAATCTCATAGATTGTGACTAGCAGTCGAACTCATATAGGTGTGATTCACAAAAGATGTTTTGTAAGTCAATACCTTTGTTTCAATAAGCCATTCGAATCACATTAAGGTATAAATCATTCTGCCATACAGCTTAGAAGTGAGATGCACCTGCAGTGGAATGAACCTTTTACCACTATGGAGTTTCGTAGATGCATTTTCTATTACCTTATGTGAGAGACTCTTTATGAATTGGTCTACCATGTTCTTAGTCGTTTGAACATAATCTAAGGCTATAACTCTGGAGTTGTTCATTTTCCTGACAGATTTTAACTGTCCCTTAACATGTCTTAAAGACTTCATGTTGTCTTTTGAACTGTTCACTTTGACAATCACCATTTGATTATCACACAGTTTATTAGAATAGCCGATATTGGGTTTTCAACTACCGGTAAATCTATTAGGAGTTCACGAAGCCACTCGGCTTCAACAATGGCGGTATCAAATGTTATGAGTTCTGCTTCCATAGTTGACTTTGTTAAGATGGTCTACTTGTAAGACTTTATAGAAACAGCACCACCTCCAAATGTGAATACATATCTACTTGTGGCTTCGGAGGCATGCTCTTGCCAGGTCCGTTCGCATATCAACATTAGGGGACCTGATTTGGGTGCCCCTGCTGGAGTTGCTCTAAGATTGTTGGATATATGATATATCAGTGCTTGTGCTTCCAGCAATCCAGTGTGTGTTTGGTTATGTTAGGTGGCTGCAGTAAAAATAGAAAAAGCTATTATTCGTAATATTATGTAATACGTACACGCAAGGGCCTCCTCGAAATCTTGTTTGGAAATGTAGTAGAGTAGTCGAAGTAGTAGGAATGTAGGATCACCTAGTAGGAAAGATGCTTGGCACAAGTCTGATCTGCTTTGGTATAGACTTGTGTCTAGTTTTATTTTTCTGTTGAAGCACCTTTTAATGATACTTTTCCTTTGAATGTTGGTATTAATTTGATACCTCTTTGCAATTATTGTATGTGCCACAATTTAATGTCTGTTGATTTCTTTGCGGTTCAGGAGCTTGTTGGAGTTTTTCCGCAACAGGTGCTATGGAAGGAATAAACAAAATAAAGACAGGCTCTCTGGTCAGTCTTTCTGAACAGGAACTAATTGACTGTGACAGATCTTATAATAGTGGCTGTGGTGGTGGCCTCATGGATTATGCTTATAAGTTTGTGATAAAAAATGGTGGAATTGACACAGAGGAGGACTACCCCTATCGTGAAGCAGATGGGACATGTAACAAGAACAAGGTAACATGGGGTTGTATAAAAGTAGCTTCACAAACTGTTTAATGCACCATGCCATGTTTATGTGATGTGTTTGCTTATATTACAGCTGAAAAAGCGAGTTGTGACTATTGATGGTTACACTGATGTACCTTCCAACAAAGAAGATTTGTTACTTCAGGCTGTTGCCCAGCAACCTGTTAGTGTAGGAATATGTGGTAGCGCTAGAGCATTTCAGTTATACTACCAGGTGCAGATACATAATTAGTTTCTCCTGTCACTAACATGTGTTACAGAACAGTAACCATTTATTTTACTTCCCTTCTAATGTTTTCTTATGCAGGGTATCTTTGATGGTCCATGTCCGACATCTCTTGATCATGCTGTGTTAATCGTTGGCTATGGTTCTGAAGGTGGCAAGGATTACTGGATTGTGAAAAACTCTTGGGGTGAAAGTTGGGGAATGAAGGGCTATATGCATATGCATCGAAACACTGGTGATTCCAAGGGTGTCTGCGGTATAAACATGATGGCATCATTTCCTACCAAAACAAGCCCGAATCCTCCTCCTTCACCAGGCCCAGGTCCTACCAAATGCAGTCTGCTCACATACTGTCCTGAGGGATCCACCTGTTGTTGCTCATGGCGTGTCTTGGGCTTTTGCCTTTCCTGGAGCTGTTGCGAACTGGACAATGCAGTTTGCTGCAAGGATAACCGGTACTGCTGCCCTCATGACTATCCTGTTTGTGATACAGGTCGCGGGCAGTGTCTCAAGGTTTGTTTGCTTGACATTTGTGTAATCTATTTGAACTTTAGCCATGTTTGTGAGGCCACTGACCTGCATCGAAGTTTTCTAGTGTTTGTTCTAAACTTTTCTAAGCTAGTTTTGTTCTTTATTTATTATGTTACCTTTCATATTTCAGTTATGAACTTTATCGTATATGGTCGTTTAAAGCATTTCAGTAGCTGACATCTTGACTGAATACTTTGATTTATGGTATCAGGCCAGCGGCAACTTCTCTGCTATAGAAGGAATCAGGAGGAAACAGTCTTTCTCTAAAGCTCCCTCGTGGACTGGCTGGCTGGAGTTGATGGACCAATGAGCGTATAATCAGACATGGATAGTAGGTAAGATTACCTAAAACTTGCTTTTGCTAGCACCGTTATACTTTCTGACGCCCATTCCTTATTGCAACACACCCATGCTGACCGTTCTGTGTCTACAACAACTAACAGCTTCATGCAAGTTGTGCGATCCGTAGGTCGGTCATTGGCCTTGTTCCAGTGCCCTGCCACATTCCTGTACGCCTACTGCCCGCTGCGTTGCACCGAGATGCCCTTATTTTCTCTTTATTATTGAACCCTATGGTGTAAAGTTTCACGATTGTACATGTATGTCACACTTGTATTGGCCTATGCATTAGACGTCTCCATTAGTTTACCTATATGATCATCT

>ZmCP28

GAGGTCAAACTAACTTCAGAGACCGGCTGCTGGTTCTCCACCAAACCAACCAACCATGCGTCCGTCGTCGCGCTACCCGCCGCAACCCGCGCTACTCGCTCTCGCCCTCGCCCTCGCGGCGGCGGCCCCGGAGCTGAGACCCGTCGCCGCCGCCGTCGCCGTCACCGTCACGCCGCCGCCGGAGCGGACGGACGAGGAGGTGCGGCGGCTGTACGAGGAGTGGAGGTCGGAGCACGACGCGGGGCCGAGGCGTGGCGCCACCGGCGGCAGCCTCGGCCCGGGCGACGCCGACGCCGGCGCCGGCGCCGGCGAGGACGACGACGCGCGGCGGCTGGAGGTGTTCCGCGACAACCTCCGCTACATCGACGCGCACAACGCGGAGGCGGACGCGGGGCTCCACGGCTTCCGCCTCGGCCTCACCCGCTTCGCCGACCTCACGCTGGAGGAGTACCGCGCCCGCCTCCTCCTTGGCTCCCGCGGCCGCAACGGCACCGCGGTGGGCGTCGTCGGGAGACGCCGCTACCTGCCCCTAGCCGGGGAGCAGCTCCCCGACGCCGTGGACTGGCGCGAGCGCGGCGCCGTCGCCGAGGTCAAGGACCAGGGCCAATGCGGTGGGTGCTGGGCGTTCTCGGCGGTGGCAGCGGTGGAAGGGATCAACAAGATCGTGACAGGCAGCCTCATCTCGCTGTCGGAGCAGGAGCTTATCGACTGCGATAAGTTCCAGGACCAGGGCTGCGATGGCGGCTTAATGGACAATGCTTTCGTGTTCATGATCAAGAACGGCGGGATCGACACCGAGGCCGACTACCCGTTCACTGGCCACGACGGGACGTGCGATCTCAAACTGAAAAATACAAGGGTTGTATCCATTGATTCGTTCGAGCGTGTGCCCATCAACTACGAGAGAGCGTTGCAGAAGGCCGTGGCGCACCAGCCTGTTAGTGCCAGCATTGAAGCATCTCGGCGCGCGTTCCAGCTCTACAGTTCTGGCATCTTCGACGGGAGATGCGGGACGTACCTGGACCACGGTGTGACGGTGGTGGGGTACGGCAGCGAGGGCGGCAAGGACTACTGGATCGTGAAGAACTCGTGGGGCACCCAGTGGGGCGAGGCCGGGTACGTCCGGATGGCGCGCAACGTCAGGGTGAGGCCGCCAAGTGCGGGCATCGCCATGGAGCCGCTGTACCCGGTGAAGGAGGGCCCGAACCCGCCGCCGGGCCCGACGCCGCCGTCGCCCGTGAAGCCGCCCAACGTGTGCAACGCGGAGTACTCGTGCCCGGAGGCCACCACCTGCTGCTGCGTCTCCGAGTACCGCGGCAAATGCCTCGCCTACGGCTGCTGCGAGCTGGAGAACGCCACCTGCTGCGAGGACCACTCGTCCTGCTGCCCGCACGACTACCCCGTGTGCAGCGTCCGGGACGGGACCTGTCGTAAGAGCGCCAACAGCCCGATGATGGTGAAGGCTCTGCAGCGCAAGCCGGCGATGTATACTGGCGGCGGCGGCGGCGGCGAGCAAAGCGGGAGGAGCAGCTGGTGAGGAGATTGAGACTGGTGGTGCGTGGCGTGTACTGTTCATCGTTCAGACAGATGACTTGCTGGCCGTGCTGTGGGCTCAGGAACTGCTTCTTCACAGTGGCGATGTTCTGATCTGTAATGCGCGAAGCACGATACTATTTGTTGTATATGTATGTGTAACTACAGATAAGATTAGGGAACGGTGTGAAAGAATAAAGAAACCGATGGAATAAGTAATTTGGGAAC

>ZmCP29

TCCCATCATCTCTCGCCATGCGCCCAACACGCTCCCCTGTGCCGGCTACGGCGCTGCTCCTGCTTGCCGTGGCACTGGCACTGGCCGCCACGGCGGCGGCCCGCCACTTCTACACCACCACCACCACCACCCGCGTCCCGGCGCCAGCGGAGCGGGCGGACGAGGAGGTAAGGCGCATGTACGAGGCGTGGAAGTCGAAGCACGGGCGCGGCGGCAGCAGCAACGACGACTGCGACATGGCGCCCGGCGATGATGAGCAGGAGGAGGACCGCCGGCTGCGGCTGGAGGTGTTCCGCGACAACCTTCGGTACATCGACAAGCACAACGCGGAGGCGGACGCTGGGCTCCACACCTTCCGCCTCGGCCTCACCCCCTTCGCCGACCTCACCCTGGACGAGTACCGCGGCCGCGTCCTCGGATTCCGCGCCCGCGCCCGCCGCAGCGGCGCCCGCTACGGCCACGGCCACGGCTACCGCGCCCGTCCCCGCGGCGGCGACCTCCTCCCCGACGCCATCGACTGGCGCCAGCTTGGCGCCGTCACCGAGGTCAAGGACCAGCAACAGTGCGGTCCGTAGCATATATACTCCGACCCATAGCGCGCCGTACTCTTACATATGTGTGCGTGCTCTCCGGCGAGAAGCTTGTTGCGGCTACTTATCTGATGACGATCTCTGTGGGGGGCATGCAATGCATGCATGCTTGCAGGTGGGTGCTGGGCGTTCTCGGCGGTGGCGGCCATCGAGGGGATCAACGCGATCGCGACGGGTAACCTGGTGTCGCTGTCGGAGCAGGAGATCATCGACTGCGACGCCCAGGACAGCGGCTGCGACGGCGGGCAGATGGAGAACGCGTTCCGGTTCGTCATCGGCAACGGCGGGATCGACACCGAGGCCGACTACCCCTTCATCGGAACCGACGGCACTTGTGACGCCAGCAAGGTCGGTATGGGTGCTGGCGTGCTGCTGCTTTTGCTTCGTCGATCGATGGATGGATGATGCGGTAGTACTGCTGCTAATATAACGGAGAGATCGATATGACTGTGTGTGTACGTGTTCAATTCAATGCAGGAGAACAACGAGAAGGTCGCCACCATAGATGGGTTGGTGGAGGTGGCGAGCAACAACGAGACGGCGCTGCAGGAGGCGGTGGCGATCCAGCCCGTCAGTGTCGCCATCGACGCAAGCGGGCGTGCGTTCCAGCACTACAGTTCGGTAAGCTAGCTAGCTAGCTAAGCATGCATGAGAATGAGATCCATCAGACGACGACGACGACGACCGTCACAGATTTAGGAAGAACTAGCTAGCTAGGTTCTGTGCGTGTCTGTCTGTGTGATGAACTGTTGTAGTATGCACTATAATGCAGGGCATCTTCAACGGGCCATGCGGGACGAGCCTGGACCACGGCGTCACGGCGGTGGGCTACGGCAGCGAGAGCGGCAAGGACTACTGGATCGTGAAGAACTCGTGGAGCGCCAGCTGGGGCGAGGCCGGCTACATCCGCATGAGGCGCAACGTGCCCCGGCCCACGGGCAAGTGCGGCATCGCCATGGACGCGTCCTACCCTGTGAAGGACACCTACCACGACCCCGGCACCGGCACCGGCACCGCCACGGCTACGGCAGCTGCCATGGATGTGATCAAGATGGTTCTTGCTTAGGAGGGAGCGAGCGGAGCAGGCAGCAGAGAGCCGATGGTCTTGTCGTGTTGAACTTTACATATGGTAGCTAGGTACCACTGGGGATAATTAAGTTAATCCGTTATGGTGTGGCAAGTTAATTAATATGTGCGTATCTCTTTTGATGAGTGCCATATATGATGTAATGAAGTTACATAAACTCAAATAAAGTCGTAATCGTAATGGTAAA

>ZmCP30

GCACACACCGACCGAACCGAAAAAACAGAGAATCCAAAAGAAAGGAACAGGGAGGTGGGGAAAAGCGCAGGGCGAAGAAAGGAAAGGGACGCACGTCTCGTCACGTCCTCCACCGGTCCACCCCCGCCGTCATGGCTGCCCTGGGCCTGCTCCTGCTCCTCCTCCTCGCCGTCGTCGGTGCCGCCAACGCCGCGGCGCCCGGCGGCAGAATGTCCATCATCTCCTACAACGAGGAGCACGCCGCGCGCGGGCTGGAGCGGACGGAGCCGGAGGCCCGGACGCTGTACGAGCTCTGGCTCGCCGAGCACGGCCGCGCCTACAACGCGCTCGGCGAGCGCGACCGCCGCTTCCGCGTGTTCTGGGACAACCTCCGCTTCGTCGACGCGCACAACGAGCGCGCCGCGGAGCACGGCTTCCGCCTCGGCATGAACCAGTTCGCCGACCTCACCAACGACGAGTTCCGCGCCGCGTACCTCGGCGCCAGGATCCCCGCCTCCCGGAGAAGGGGAACCGCCGTGGGGGAGAGGTACCGCCACGGCGGTGGCGCCGAGGAGCTGCCGGAGAGCGTCGACTGGAGGGAGAAGGGCGCCGTTGCGCCCGTCAAGAACCAGGGGCAGTGCGGTGAGGAACCTGGACCCTTTTCTTTCGGCTTATTAACTTGTGTCGATCAATTAGTTCTATCGAACAGAACAGATGCGGTTCCTCAGTTTGTTGACGCTAACTTTGGATGTGAAGTTCTGAACGGGCCGGTTTTGCATTAGGTCTTTAGGGTTCGATATGCCTGGCCTTTTAACTTCGTAAATGTTTGATGCTTGAGCTATTTCTTTGCGGATTTTTTTTCTATCACAACAGGTAGTAGGTCTGAAAATCTGTGGTGAAGTTGGATCTTCCCCCCTCTGTGAACTTATGAGATTTAATCTACACACAATTGTTGTTTGTAATTTGATGATTAACAACGATGAGGTTTAATAAACACACAATTGTTTGTTTGGAATTTGATGATTAACATTCATGAGATTTAATCTAAGCATAATTGTTTGTTTGGAATAGATGATTAACATTCATGAGATATAATCTACACACAATTGTTTGTTTGAAATTTGATGATTAACACTGAAGTAGCTGGCCACCTTTCTGCATGGGTATTGTTTCCCCGACGGGTTACGGTTTAGATGAACCGTACAGAAGGGGGTTATTTCTTGGTTCTTTTCTTCTGGACGAAGTAAGGGCCATGAGTCCTGCTGCGTGGAATTTAGACGGAACGAGCCCCACGGTTTCTTGGACTTTGTCCGGCCTGCTGTGCCTTCCCTATTTTTAGGGTTACAGTTTAACTATCTTCCAAACCGCTTTTTCTGGTGTAAGACTACTCTTTTATGATTGTTTCTGTTGTGGTCTTTCCATCGAAGCTGCTGTTAGTTGTTTCTGCCGGTCAGGCCTTCTTTTTTAAGGGATACGCTAACAAAACTTTACAGAACTATTTTGGGATTCTTTGGGCTCTCACATGCCATGTCATATATGAACAGGCTTACAGTTACACAACATGTTCTGTTGGCACTGCTGTACTTGGGAAGTACTGCTTTGTAGTGTTGACTGTTTGCTTGGCTTTAATCCGCTTCTACAATATTTTTTGTCTCCATTGAGCCTGTGGTTCTCGTATAATACCTTGCTGGGTGAGACCTCCTAGGATTTTGTCTCTTGCAAGAATTAGAACACCTATGGTTGCTTGTAAAAATTGGCCACATGGATTCAAGAAGGCGAACACTGTAAAACAAATTATCTGATTAAATGTCACAGTTTATCTATGTTATCCTGGTAGCGTTAATATGAGGATCTGGCTGCACACTCTTCATATGTATAAAGTCAGTACTATTGTAGCTTATGCAGTCTATCTATTTCTGTTGTTCAGAATTTGTTATTGTTGAATTATGATATGCAGGAAGTTGCTGGGCTTTCTCTGCAGTAAGCTCAGTAGAAAGCGTTAACCAAATTGTTACTGGTGAGATGGTGACCTTGTCCGAGCAGGAGCTTGTAGAGTGCTCGACTGATGGAGGGAACAGCGGCTGCAATGGTGGGCTTATGGATGCCGCATTTGATTTTATCATAAAGAACGGGGGCATTGATACTGAAGGAGACTACCCTTACAAAGCTGTGGATGGGAAGTGTGACATCAACAGGGTACATTACATTGTCTAACCCTCCTTCCTCTGATGCTGTCGCTGGTAAACTCACCTTCAGATTTATGCATTGGTCACTGCAGGAAAATGCCAAGGTTGTGAGCATCGATGGCTTTGAAGATGTGCCTGAAAACGACGAGAAATCATTGCAGAAGGCAGTTGCTCACCAGCCCGTCAGCGTTGCCATTGAGGCTGGTGGCCGGGAGTTCCAGCTGTACAAAGCGGTAAAACCTGCTGCAGTTAGCAGCATATGCTATGGCCCAGCAAGGTTTGGCTGCTGTTCTTACACTCGTCTTTCGGTTTTCATTTAGGGTGTCTTTACTGGAACTTGCACCACGAATCTTGACCACGGTGTCGTCGCTGTCGGCTACGGCACCGAAAATGGGAAGGACTACTGGATTGTCCGCAACTCTTGGGGTGCAAAGTGGGGCGAGGATGGTTACATCCGTATGGAGAGGAACGTCAATGCCACCACTGGGAAGTGCGGGATTGCTATGATGGCATCCTACCCTACCAAGAAGGGCGCAAACCCTCCCAAGCCGTCTCCAACCCCTCCAACGCCGCCGCCTCCCCCGGTTGCCCCGGACAATGTCTGCGATGAGAACTTCTCCTGCGCAGCGGGCAGCACCTGCTGCTGTGCGTTTGGTTTCAGGAACGTCTGCTTGGTCTGGGGTTGCTGTCCGATGGAAGGTGCCACCTGCTGCAAGGACCACGCCAGCTGTTGCCCGCCGGGTTACCCTGTGTGCAACGTCAGAGCCGGAACTTGCTCGGTGGTAAAGTCTTTGCTCCTTAATTAATGTTTTCACGGCAGTAACTACACTTGGAGTTGTAGGTATAGCATGCTGTTTGCGCGTTATTTGTGTTTATCACGGTTGATTTAACCATAATAGCAGGCTGTTACATTTAAATTACTCCTTGTTTAAATTGAGAAGAGCAAGAACAGCCCAATTTTGTTTGTGTTCGTCATGATCTAGCCATAAAATAACATGCGGATGCATACTAAACCCTCTATTTGTTTTTTTATATAAAAAAATCGAGCAGAGCAAGAACAGCCCGCTGAGCGTGAAGGCCTTGAAGCGCACCCTCGCCAAGCTGAACAGCGCATGAGCTCGTCCAGACATCAGGGATCATCGATCTGGAGAGGAATGGGACCACCGCCCGGAGCTTTTCAGACTCTGCGTCCTGATACAAGCTTTCATAGATAACATTATATTGTATCTAAGTATTACTTGGCGTCCCATGAATCCGTTGTAAATATCAAGCTATAATTTCTACTATCTTTGTTCTCGAA

>ZmCP31

ACCCCACACCAAAAGAAACCAACTAAAATCCCAAAAGAGAGGAAAGGCACAAACAGACGGTACAAAGAAAGGGACGCTCGTCTCGTCTCGTCACGTGCCGCCGCCATGGCTGCTCTGGGCCGTGGCCTGCCCCTCCTCCTCCTGCTCCTCCTCCTCGCCGTATCGGGGGCCGCCAACGCCGCGGCGGCGCCCGGCGGCATGTCCATCATCACCTACAACGAGGAGCACGGCGCGCGGGGGCTGGAGCGGACGGAGCCGGAGGTGCGGGCCATGTACGACCTCTGGCTCGCGGAGCACGGCCGCGCCTACAACGCGCTGGGCGAGGGCGAGGGCGAGCGCGACCGCCGCTTCCTCGTCTTCTGGGACAACCTCCGCTTCGTCGACGCGCACAACGAGCGCGCCGGCGCCCGCGGCTTCCGCCTCGGGATGAACCAGTTCGCCGACCTCACCAACGACGAGTTCCGCGCCGCGTACCTCGGCGCCATGGTCCCCGCCGCCCGCCGGGGAGCCGTCGTGGGGGAGAGGTACCGCCACGACGGCGCCGCCGAGGAGCTGCCGGAGAGCGTCGACTGGAGGGAGAAGGGCGCCGTCGCGCCCGTCAAGAACCAGGGGCAATGCGGTGAGGAACCTCGACTCTTGGTCCCCCGGCGTGTTATCTTTTGCCGATCAGTTCTTGCTGTCGAACAGAACCGGACGTGGTTCCTCGGTTGGTTGACGCTAACTTCGGATCTGAACAGAATCTCCGGTTTTGCATTAGATCTTTTAGGGTTCATATTGATATGTCCTTTTTAACGCTTGCAAAGCTCTGCAGATTTCTGATCGGTGCTTAAACTATTTCTTTTTAAGGACACTTAATTTTGCGGAAAAATAGTTTCCCCCCCTATCATAACAGTTAGTGGGTGTGTAAATCTCTAGTGAAGTTATATATCTTTATTTTCTTTGTGAACATATGAGATTTAATCAGCACATCAATTATTCGGGATATGATGATTAACATTGAAGTAGCTAATCAATTGTTTGGGATATGATGATTAACATTGAAGTAGCTGGTTCACCTTTGCTTGTTTTTTTTTCAACGGGTTAGCTGAACTGTACAGATGGGGCTTGTTTTCGTGTCTCTTTTCTTCTGGACGAAGCAATGGCCATGCGTCGTGCTGTGTTGAATTTGGACGGAACTAGCCCCACCACCGTTTCTTGGGCTGGTCCTGCGTGCTGTGCTTTCCCTATTTTGAGGGTTACGGTTTAGCTATCTTACAAATTGCTTTTCTGGTGTAAGACTACTGTTTTAGGATTGTTTCTGTTATTGTTTCTGTCGATGGACTTTCCACCGAAGATCCTGTTGGTTGTATCGACCTGTCGGCAGACTTTCTTTTTGAAGGAGTTAAGGGCCTGTTTGGTTCGTTTTGTCCAGAACTAAAGTTTAGTTGAGTGACTGAAGTTTAGTCCATATTATGTTTGTTTAAAGAGACAAAATATTCAGAATATATTAAATGACTCATAAGAGGACTAAAATGCTTCTTAACATTCTTCCGCTAGTAGTGAAACTGAAATAAATGAAGGCTAAAAAGTGGAATTAATATGGTTTAGTCCTTTTTAGTCACCCCTTGAGGAACTAGAGACTAAATCAGTTTAGTCATTGTTTTAGTCCACCGTTTGGCTATTTAGAGACTAAATGAGACTAAAATGGAGGGGTTAATCTTTAGACCCTCAACCAAACAGGGCCTACTCAGAATCTGTATGAAACCTACTTTGGGGTTCTTCGTGCTGTCACATGCGTGTCATATATGCACAGCATGTTCTGTTGGTACTTGGTAGTACTGTAGTCGGTAAGCCTGTTTTTTTTTCCTTCTGTATTATTATAGAACCTGTGGTTCTTGTATTGTAGCCTGCTGAAACCACCTATGATATTTGGAAAAAAAATTCTTTGCAGTCCAGATGCAAACGTACTCCTTTACAGCCTCTCACATCCACCACTTGGAGGTCCACATGAACAAATTGCTAAAGGGTTAAGTTGCAAATTTTGATCTGATTTTATGGGCCTCTAAGTGGTCGATGTGAGGGACTGTACGTTTGCATCATCCGGGCTGTGGAGATTTTTTTTCCTGATATTGTCTCTTGCAATAATCAAAACAACTATGGTTTGATTAGAAAAAAATACTGTAAGACAAATGAGTTGATTACGGATCACAGTTTATCTATGTTATCCTGGTAGCAGCGTCATATGCGGTTAGGTTGACACTCTCCACCCTTCATATGCATTTAGTCAGTACTATTGTAACTTACGCAACCTGGGGAGGAGATTTATTTCAGTTGTTCACAATTTGTTCTGGTTGAACTATGGTGTGCAGGAAGTTGCTGGGCTTTCTCTGCAGTAAGCTCAGTGGAAAGCGTTAACCAGATCGTCACCGGTGAGATGGTGACACTGTCTGAACAGGAGCTCGTAGAGTGCTCGACTGACGGAGGGAACAGCGGCTGCAACGGCGGGCTCATGGACGCCGCTTTCGATTTCATCATAAAGAACGGGGGCATCGATACCGAAGATGACTACCCTTACAGAGCCGTGGACGGGAAGTGCGACATGAACAGGGTACATTGCCTAACCACCCTCCCTTCCGTCGTGTCCCTTCCTTACATGGCTGTCGCTGGTAAACTGACGTTGCGAATTGTGCATCTGTCACTGCAGAAAAATGCCAGGGTTGTGAGCATCGATGGCTTTGAAGACGTGCCTGAGAACGACGAGAAGTCGCTGCAGAAGGCGGTTGCTCACCAGCCAGTTAGCGTTGCCATTGAGGCCGGAGGCCGGGAGTTCCAGCTCTACAAATCGGTAAACGAACTTCGCCGTAGCTAGCAGCCCTGTGTCTGTGGCCCAGCTGTACTGTTCTTAACAACGTGTCTTTGTTTTTTTTTTCCGTCAGGGTGTCTTCAGCGGAAGCTGCACCACGAACCTTGACCATGGTGTCGTCGCGGTCGGCTACGGCGCCGAGAACGGGAAGGACTACTGGATCGTCCGCAACTCGTGGGGCCCGAAGTGGGGCGAGGCTGGTTACATCCGTATGGAGAGGAACGTCAACGCCAGCACTGGGAAGTGCGGGATCGCGATGATGGCGTCCTACCCGACGAAGAAGGGCGCGAACCCTCCCAGGCCGTCTCCAACCCCGCCAACACCGCCGGCTGCCCCTGACAATGTCTGCGACGAGAACTTCTCGTGCTCCGCGGGCAGCACCTGCTGCTGCGCGTTTGGCTTCAGGAACGTCTGCTTGGTCTGGGGCTGCTGCCCGGTCGAGGGCGCCACCTGCTGCAAGGATCACGCCAGCTGCTGCCCGCCGGGCTACCCTGTCTGCAACGTCAGAGCTGGAACTTGCTCGGTGGTAAACTCTCTGCTCCTTGATCAATGTTTTCTTTTTTTTTTCACGGCAACACTGGTATATCTACTTACTGTCTGGCCTAATAGCATGCTGTTTGTGCTTTTAACCAGACTAGTATACTTAACAGCATGCATGAGCTAATAACCGAGAGATATCTTTGTTTTGTTTGAACTGAGCAGAGCAAGAACAGCCCACTGAGCGTCAAGGCCTTGAAGCGCACTCTCGCCAAGCTGAGCACCGCATGAGGACCTCGATCTGGGACTGCCCATTGCTTTTTCTGACTCATGGGCGTTCAGATCACCAAGAGCAAGATTTCATAGATTATCTATATGTTTGTATAAGTACTATTACCGTCCCATGAATCCGCTGTAAATAACTAGTACCTCTGATTAAAAGGATAATATTATTTGTC

>ZmCP32

GAAGGGCCTCCTGTATCCTCTCACTTCCCTCAGTCCTCGTCCGTCTGCTCCCTCCTCTCTCTACCTAGCTCTAGCTAGCCTTCCTGTAGCGCGGCAGAGATGGCTGCCCCTGCCTCCACCACGGCGGCGGCGGCGGCAGCGCTGCTGCTGCTGCTCCTCCTGTCGCTCGCCGCGGCGGCGGACATGTCGATCGTGTCCTACGGGGAGCGCAGCGACGAGGAGGCGCGGCGGATGTACGCGGAGTGGATGGCGGCGCACGGCCGGACCTACAACGCCGTCGGCGAGGAGGAGCGGCGGTACCAGGTGTTCCGGGACAACCTCCGCTACATCGACGCGCACAACGCCGCCGCCGACGCGGGCGTCCACTCCTTCCGCCTCGGCCTCAACCGCTTCGCCGACCTCACCAACGACGAGTACCGCGCCACCTACCTCGGCGCCAGGACCAGGCCGCAGAGGGAGAGGAAGCTCGGCGCCAGGTACCACGCCGCCGACAACGAGGACCTGCCGGAGTCCGTCGACTGGAGGGCCAAGGGCGCCGTCGCCGAGGTCAAGGACCAGGGCAGCTGCGGTAAAGGAGCTTCCATACCAATCTCCGCCCTCTTCCTCTCTCTTTTTTTTATTACTATACTCCTGCTGAAATAAAGCTGACCTGCTTAGTAACTCTAGCTAGAACATGGGAAGCTTCTCCAGCCGTGTCCATCCGCAACTTGCAGTAGTTTTTATTGCTATCCACATGAGCAGAATAAAAGAGTCGGGGATTTGTATCTTCTACGGTTGTACCTGAAAGGTCAACGCTCAGCGGCAAGCTTCACGTTCTGCAATTCCGTTAGTCGTGCGTCCAGAATGGGTGGGAGGTTGCAGAGATGGCACGCTTTTGAATCTGAACTCCATAGATAGATAGACACGCTAATTGACAAATAAAATCGTTTTAAATCAAAATATTCGATTTGGGTACAGCTTTGTTTCGGTTCTACACCTAGTTTTATGGGTTCTGTGACTTCTGGGGTAGGAGTTGTTTGGATCTCGGACTAAACTTTAGTCCGGGTCACATCCGATGTTTGCATTTCGATTAGAATGATTAAACATGAGTTTATTATAAAACTAATTGCATAAACAGTGACTGATTCGTGAAATGGATCTATTAAACCTAATTAATTTGTGGTTATCATATGTTTACTGTAGCATTCACGTAGGTAAATCATGGATTAATTAGAATAGATTCCATCTTGTGAATTTGCTATGGTTTATACAATTATTTTTATATAATTATATTATCTTTATTGTCTTGCGTGAAACGGGACATACACTATGTAAGTAGGTTCACCGAACATGGACATCACTTTGCTCCCTTGTGAACTCCAGTCTCCAATGGGGCAGTGACCCTGCTGGAAGGAGCAACCAAGAAATATATTGCACTAATAGCAGTCGCTGTCTAGCTGACAAGGCGGTTGGATTTCTCCTCTGGACAGGCTCACTTCATGATTCCAGCTAGTAGCTCACTTTCATCACGGCTCTTGTGAACATTTCACGTTGTATTGGTCCCCTGCCTCCCTCGCCAATGCCATCCATTCATCACCACAAATTTCTTTCTCTTGAATAAAATATGCTACGCCCATAAAGATATTTTCCACTCTATTGTGGGTAGCTTTCACTCTTTTGGTTTTATAGCTATGTGCTGTTACTCGGCTGCCTTTTTTAATTGATATGCGGGATTTAACAATGATACTGATGGTTTATTGACATTGAAATCTGATCTCTTTCAGGGAGCTGTTGGGCTTTCTCAACAATAGCAGCTGTGGAAGGCATCAACCAGATTGTTACAGGCGACTTGATCTCCTTGTCTGAACAAGAGCTTGTCGACTGTGACACTTCGTACAATCAGGGGTGCAATGGAGGTCTGATGGACTATGCATTTGAGTTCATCATCAACAATGGCGGAATCGACACCGAGAAGGATTACCCTTACAAGGGCACGGACGGACGGTGTGATGTCAACAGGGTATTTTTTGCTTCCATAGTTTTTCCTTGATTTTCCAAAGACCAGCAGCGTCATTTTTACTTACTGCTGATCTTGTGTGTTGGGTCCATCTATTGCAGAAAAACGCGAAGGTTGTCACTATTGACAGCTACGAAGATGTACCAGCGAACGATGAGAAGAGTCTGCAGAAGGCAGTTGCAAACCAGCCTGTCAGTGTCGCAATTGAGGCTGCTGGCACAGCATTTCAGCTCTACAGCTCGGTAATTGGCGCTCACCTTGCTGCTGGGTTCATCTGTGGACCATTTGGTTAAAGTCCGAGATGAATGATTCAATCATCTAGTGTTTGATTATTAGTTGGTGGCCATGAAAGAAATAAAATCCATCTGCCTTGAACAAGACATGCCCATGTTAGGTCGAGGTCACCTAATTGCTTTAAAATTGTCTCTTTCATATCGCTTTACTCAAGATTATTTTCTCTGGAAAGAATGATTCTGTTGTCACCCTGCAAGCTGCAACTGTTACACACCATACCCCTGACCTAGTTTACTTCGATCCAGACTATATATTTAGCAACTTTTACAGTACGATAGTCCTTCAGTGCCCCACCACTGAATCATGCTCCCTTGTTTTTCTCAAAGACTATCCTGCCACTTCCTGTGAAGCACTTTTAATGGTATACAACTTTGATGTGTTTCTGTGAGGCCTTTTGGAAAGACGTGCTTTGGTAATATAGCGGCGTGAGCTATCCTCCACTAATTAATAATTATCATTTCAACTCCATGAATCATCTGCCTTTGTACTGGTGACTAATTATCGTTTCCTGGCCTCAGGGTATCTTCACTGGAAGCTGTGGAACAGCGCTGGACCATGGTGTCACGGCCGTCGGCTACGGGACAGAGAACGGCAAGGACTACTGGATCGTGAAGAACTCATGGGGCAGCAGCTGGGGCGAGTCCGGGTACGTGAGGATGGAGCGCAACATCAAGGCGTCCAGCGGCAAGTGCGGTATCGCGGTTGAGCCGTCATACCCGTTGAAGGAGGGCGCCAACCCGCCGAACCCTGGCCCCAGCCCGCCGTCCCCGACCCCGGCGCCCGCCGTGTGCGACAACTACTACTCGTGCCCTGACAGCACCACCTGCTGCTGCATCTACGAGTACGGCAAGTACTGCTTCGCCTGGGGCTGCTGCCCGCTCGAGGGCGCCACCTGCTGCGACGATCACTACAGCTGCTGCCCCCATGACTACCCCATCTGCAACGTCAGGCAGGGAACCTGCCTCATGGTAAATGCTCGGCCCATCTCGTGATCTTCTTCAGTTAGCTACGGAGATAACTGCCAGTTTCTTTCGTCTGATCTCTAACCGGCAAATTCGCTGCTTGACGAACAGGGCAAGGACAGCCCACTGTCACTGTCAGTGAAGGCTACGAAGCGAACCCTGGCCAAGCCGCACTGGGCTTTCTCCGGCAACACAGCTGACGGAATGAAGAGCAGCGCATGAGAAAACGCAGCGTGGCATTAGGTCGTCAGCAACCTGGGTGGTCGATTAGAGCCCCTGCGTGGGAAATGGTCACCTGGCCCCCTGATTGACGACGGAAGCATGGCTCG

>ZmCP33

ACGTCCAAGAAATGAATATAGCATAACATTGCTGATTGTTCCGATAAATACTTCCATTACACCGAATATCAATCGCAGTCCTGTTGAACTGCGCAGGGGAAGCTTGGCAAGAGCTAGCTGGATTTTTACATGGCATGCAAAAGCAAATTTGGCAGAGTTCCATAAACAACTGAACCCATTTTTTTTCAGGCTTATGTACAGGAGGAGTACCTACTATAGTGATATAAGTAAATCCGTAAACAGCGGCCGCCCAGCCATGCTTTCGTCGTGAACTACGGGACCGGGTGATCATTCTGCATGCAGGGGGGCTCTAATCATGCTGGCTGTTGGCGGCCTAATGCCATGCTGCGTTTTTCTCATGCGCTGCTCTTCATGCCGTCAGCAGCTGTGTTGCCAGGGAAAGCCCAGTGCGGCTTGGCCAGGGTGCGCTTCGTAGCCTTCACTGACAGTGACAGTGGGCTGTCCTTGCCCTGTTCGTCAAGCAGTGGATTTGCCCGTTAGAAACCAAACGAATTTGACCACTGTAGAAGATCATGAAATGGCCCGAGCATTTACCATGAGGCAGGTTCCCTGCTTGACGTTGCAGACGGGGTAGTCATGGGGGCAGCAGCTGTAGTGGTCGTCGCAGCAGGTGGCGCCCTCGAGCGGGCAGCAGCCCCAGGCGAAGCAGTACTTGCCGTACTCATAGATGCAGCAGCAGGTGGTGCTGTCGGGGCACGAGTAGTAGTTGTCGCAGACGGTGGGCGGCGGGGTGGGGGAAGGCGGGGTCGGCCCAGGGTTCGGGGGGTTGGCGCCCTTCTTCAGAGGGTACGACGGCTCAACGGCGATACCACACTTGCCGCTGGACGCCTTGATGTTGCGCTCCATCCTGACGTAGCCGGACTCGCCCCAGCTGCTGCCCCAAGAGTTCTTCACGATCCAGTAGTCCTTGCCGTTCTCTGTGCCGTAGCCGACGGCCGTGACACCATGGTCCAACGCTGTTCCACAGGTTCCTGTGAAGATACCCTGAGAGGCCAGGAAAAGATGATTAGTCACCAGTACAAGGCAGATGATTCATGGAGTTGACACTAATAGTGACAACAACGAAGGATGAGATAATAAAATGCCTATTTAACTTTATGGCAAGGGAAACATTTTTCAGAATCTTCTGCTCGGCTTTTGCTCGAAGTGGATGATAGCTCACACCACTCTCCTACCAAAGGACGTCTTTCCAAAAGCCCTCACAAAAACAGATGAAAACTCTGTGTAGCATTAAAAATTGCTTCACAAGAAGTGGCAGCATAGTCTTTGAGAAAAACAAAGGAGCATGATTCGTGCGAAGTGGTGGGGGTACTGAAGCACTATTGTATCGCAAAAGTTGCCAATACGTAATCTGAATTGTAGTAAACTAAGTCGGGGTATGAACTATGAACAAGGTATCATAATTGATGTAGTAGTTGCAACTTGCGGAGTGACAACAGAATCATTCCTTCCAGTGAAAATAACCTTGAGTGTAGAGATGTGATGTCTTGTTCAAGGCAGATTTTTTTAAAAAAAAGCAATTAGGTGACTCGACTCGAACAATCATGTGCATGTCTTGTTCAAGGCACATCTTTTTTTTTTCTTTCATGGCCATGGATATACCATCAACTGATTCCCACCCACTGATAATCAGATACTAGCTGATTCCATAATTCATCTACGAATTTAACGAGATGGTCCACAGATGAACCCAGCAGCAAGGTTAGTGTCAGTTACCGAGTTGTAGAGCTGAAATGCTCTGCCACCAGCCTCAATTGCAACACTGATGGGCTGGTTCGCAACTGCCTTCTGCAGACTTTTCTCACTGTTCGCTGGTACATCTTCATAGCTGTCGATAGTAACAACCTTGGCGTTTTTCTGCGATGAACCCAACACATAAAATCAGCAAGAAGAAAAAAATGACACTGATATTTGGAAGATCAAGGAAGACCATGAAGCGAAAAATACCCTGTTGACATCACACCGTCCGTCCGTGCCCTTGTAAGGGTAATCCTCCTCGGTGTCGATTCCGCCGTTGTTGATGATGAACTCAAACGCATAGTCCATCAAACCTCCATTGCACCCCTGGTTGTATGAAGTGTCACAGTCAACAAGCTCCTGCTCAGACAAGGAGATCATGTCGCCAGTAACAATCTGGTTGATGCCTTCCACAGCCGCTATTGTTGAGAAAGCCCAGCAACTCCCTGGAAAAGATAAACATATATCAGATTCCAGTACCAATATGTTATCAATATTATTGTTTAGTCATCCACGTCGATTAAAAAAGGCGGACAAGTAACAAGTCAGAGTTATAAACTCCAAAGAGTGAAAGTTAACCTGCAATAGAGTGGAAAACATCATTATCACTTTAGACCTAGTTTAGGTACTATAGTAATGAGAAAATCGGAGTGGATTAAGATGTATTGTGAGTGTTTTTTTATCTATTGCGGATGTAAATACTCTCTAATCCCTCCAAACCACTTCAATATTAGAGTACTCAAACTAGACACTTTATGGACATAGCATTTTTTTTAAGAGAATAATTTTTTGGTTGTGACCGGTGAATAGGGTGTGGACCAATAGAAACGTGAAACGGTCACAAGAGCCGTGAATCCGTGATGAAAGTCGTCTCCAAGAGCCTACTATCTGGAATCATGAATGAAGTGAGCATATTGGCCCAGCCACCTTGTCAGCTAGACAGCGACTGCTAGTGTAAATATCTTTCTTGGTTGCTCCTTCCAGGAAGGTCACTGCCTCGCAAGGTTTAAAGGTGATGTCCATGTTCGATGAACCTACCTACATAGTATGTGTCCCCCTTCATGCAAGGCAATAAAGATCAAATACACGGATCACTTTTCCACCTTCCGTAGCGAAAATCGTTCTACTAATATTTGTCACGCCCACGCACAAGACACAGAACCAGTACCAGTACCAGTACCAAATGGAATATTTTTAAATAGTTTTATTTGTTAGTGTGTCTCGAATTCAAGTGCATGCTAATCTATATACGCGTGGCATCTGCGCAACCACCCACCCGTTTTGGTGCCCGGCTAACGAATCGCAGAACGTGAAGCTGAGCGTTGACCTTCAGCTAGTCCCCTGCTCTCTCTCTCTCTCTCTCTCTCTCTCTCTCTCTCTCTCTGTTCATGTGGATAACAGTAACAAACTACCGCAAGTTGTGGATGAGCAGACTTGACAGTTTTCCCCATGTTCTATCTAGAATTATTCTTGATTCAGAAGCAGGAGAGGGATAAAAAAAAGAAGAAAGTGGAAGGGCGGAGATCGATATGGAAGCTCACCGCAGCTGCCCTGGTCCTTGACCTCCGCGACGGCGCCCTTGGCCCGCCAGTCGACGGACTCCGGCAGGTCCTCGTTGTCGCCGGCCAGGTACCTGTCACCGAGCCTCCTCTCCCTCTGCGGCCTGCTCCTGACGCCGAGGTAGGTGGCGCGGTACTCGTCGTTGGTGAGGTCGGCGAAGCGGTTGAGCCCGAGGCGGAAGGAGTGGACGCCCGCGTCGGCGGCGGCGTTGTGGGCGTCGACGTAGCGGAGGTTGTCCCTGAACACCTCGAACCTGCGCTCCTCCTCGCCGACGGCGTTGTAGGTCCGGCCGTGCGCCGCCATCCACTCGGCGTACATCCGCCGCGCCTCCTCCTCGCTCCGCTCGCCGTAGGAGACGATCGACATGCTGTCCGCCGCCGCGGCGAGCGGCGCGAGCAGCAGCAGAAGCAGCGCCGCCGCCGACGCGAGCGGCGTGGTGGAGGCGCCCATCTCCGCCGCGATGCAGGAACGGGAAGGCTAGCTAGATAGGGGGAGGAAGGAGCGGAACGGACCGGAGCAGAGCAGATGGATGAGAGTTGAGATGGAGGCCCTTCCTTTTAATACAAGTGGGCGAGGGAGCGGTGGGCCCAGATTCCGTGATCCCCTTGTTTTTTTAAATTCATTCGCATCTAATCTACTGGACCAGTCAGCTGTACGATATTTTCCGTTGGTTTTTTATTGGTCGTGGGGTTGTGGAGCGGAAGTGGACAGGTTGGATTGGATTGGATGAGGGCGACCTTTTTTATTCTTACCCGA

>ZmCP34

CTGCAACAGCCAAGTGCAAGTGATCGAGCTCCCCCCTCCTCATCAGTCGCTAGGCACAGCGCACTTGCAGGATGGCGCAGGTCTCTAAGACGCTTCTCCTTGTGGCCCTGGTGTTCGTGTCGTCGGCGGCGGTGGAGCTGTGCCGCGCGATCGACTTCGACGAGCGGGACCTGGCGTCGGACGAGGCGCTGTGGGACCTGTACGAGCGGTGGCAGACGCACCACCGGGTGCACCGGCACCACGGCGAGAAGGGCCGGCGGTTCGGGACGTTCAAGGAGAACGTGCGCTTCATCCACGCGCACAACAAGCGCGGCGACCGGCCCTACCGCCTCCGCCTGAACCGGTTCGGCGACATGGGGCGGGAGGAGTTCCGGTCCACGTTCGCCGACTCCCGCATCAACGACCTCCGGCGGCAGGATTCCCCCGCGGCGCGGGCCGGGGCCGTGCCGGGGTTCATGTACGACTCCGCCGCCGACCCGCCGCGGTCCGTGGACTGGCGGCAGGAGGGCGCGGTGACGGGCGTGAAGGACCAGGGCCACTGCGGAAGCTGCTGGGCCTTCTCGACGGTGGTGGCCGTGGAGGGCATCAACGCCATCCGGACGGGCAGCCTGGCGTCGCTGTCGGAGCAGGAGCTGATCGACTGCGACACGGACGAGAACGGGTGCCAGGGCGGGCTGATGGAGAACGCCTTCGAGTTCATCAAGTCCTTCGGCGGCATCACCACCGAGGCCGCGTACCCGTACCGCGCGTCCAACGGCACCTGCGACGGCGATCGGGCCCGGCGCGGGGGAGGCGTCGTCGTGGTGATCGACGGCCACCAGATGGTGCCGGCCGGCAGCGAGGACGCGCTGGCCAAGGCGGTGGCGCACCAGCCGGTGTCCGTGGCCGTCGACGCGGGGGGCCAGGCGTTCCAGTTCTACTCGGAGGGCGTGTTCACGGGCGACTGCGGTACGGACCTGGACCACGGCGTGGCGGCGGTCGGCTACGGCGTCGGCGACGACGGCACGCCCTACTGGATCGTCAAGAACTCCTGGGGCACCTCCTGGGGCGAGGGTGGCTACATCCGGATGCAGCGCGGCGCCGGCAACGGCGGCCTCTGCGGCATCGCCATGGAGGCGTCCTTCCCCATCAAGACCTCGCCCAACCCTGCAGACCCGCCGCGCAAGCCCCGCCGCGCGCTCATCGCCAGGGACACCTCTTCTTCCCAGTGACACACACGACCACAACTGGCACGTAAGTACACGTTGCCAGGAATGACATGCTATGCTACTAGTGTTTGCTTGAACTATTATCGATTGGGTTTGTACTAGTGATGACAGTTTCATCGATTGCGCGGTCGATCTCCATCAGAGACAGAGACGATATAATCAGAATAAAGTATAGCAGCGCGTGTGCGCAACAGGGTAAGATTAGGCGTGTGATTTGATTTCAAAGCTGTGCTTGTAACGTAATGTAATACGGTGTGGCATATGCACCGGGATATGGTATGTGTATTGGGAAGACGCTATGCGTAGC

>ZmCP35

GCTCACACTCCGGCAGCCGAGCGATCGAGCTCCCCGGTCGCTAGCACAGCCGGCCACTGCACACGACGGTGCTGGTAGGTTGCAAGCACTTGCAGGATGGCGCAGGTCGCTAAGACGCTTCTCCTTGTGGCCCTGGTGGTCGTGTCGGCGGTGGAGCTGTGCCGCGCCATCGAGTTCGACGAGCGGGACCTGGCATCGGACGAGGCGCTGTGGGACCTGTACGAGCGGTGGCAGACGCACCACCGGGTGCACCGACACCACGGCGAGAAGGGCCGCCGGTTCGGGACCTTCAAGGAGAACGCGCGCTTCATCCACGCGCACAACAAGCGCGGCGACCGACCCTACCGCCTCCGCCTGAACCGCTTCGGCGACATGGGCCGTGAGGAATTCCGTTCCGGGTTCGCCGACTCCCGCATCAACGACCTCCGCCGGGAGCCCACCGCGGCGCCGGCCGTGCCGGGGTTCATGTACGACGACGCCACCGACCTGCCGCGGTCCGTGGACTGGCGGCAGAAGGGCGCGGTGACGGCCGTCAAGAACCAGGGCCGCTGCGGCAGCTGCTGGGCCTTCTCCACAGTGGTGGCCGTGGAGGGCATCAACGCCATACGGACGGGGAGCCTGGTGTCGCTGTCGGAGCAGGAGCTGATCGACTGCGACACGGACGAGAACGGGTGCCAGGGCGGGCTCATGGAGAACGCCTTCGAGTTCATCAAGTCCCACGGCGGCATCACCACCGAGTCCGCGTACCCGTACCACGCCTCCAACGGCACCTGCGACGGCGCGCGGGCCCGGCGCGGGCGGGTCGTGGCGATCGACGGCCACCAGGCTGTGCCGGCCGGCAGCGAGGACGCGCTGGCCAAGGCGGTGGCGCACCAACCGGTGTCCGTGGCCATCGACGCGGGGGGCCAGGCGCTGCAGTTCTACTCGGAGGGCGTGTTCACGGGCGACTGCGGCACGGACCTGGACCACGGCGTGGCGGCGGTCGGCTACGGCGTCAGCGACGACGGCACGCCCTACTGGATCGTCAAGAACTCGTGGGGCCCTTCCTGGGGAGAGGGCGGCTACATCCGGATGCAGCGCGGCACCGGCAACGGCGGCCTCTGCGGCATCGCCATGGAGGCGTCCTTTCCCATCAAGACCTCGCCCAACCCGTCGCGCAAGCCCCGCCGCGCGCTCATCACCAGGGACGCCTCTTCCCAGTGACAGGCTGATCCATCTCGGCGTCGAACTCGTACTACTGGCACGTAAGTAGGAGTACGTTGACAGGATGACATCCTGCTGCTGCTGTTTGCTTGTACTATTTTCGATTGTGTTTGTAGGTTTGATTGTGCCAGTCTCGATCAGAGACGATATACACATGCTAGAATAAAATATAGCAGCGCGTGTGCGCAACAAAGACTAGGGCCTGTTTGATTTGATGCCTAACTTGCCACACTTTGCCTAACTTTTGTGCCTAAGGTTAGTTATTCAATTCGAACGACTAACCTTAGACAAAGTGATGCACATTTAGCTACAAACCAAACAGCCCCTAGGAGTGTGATATCAAAGTTGTGCTTGTAATGTAACGTAATACGGTGTCGCATATGCACCGGGATATGGTATATTGAATGGAGCTATGCGTAAGCTAGCAGTTGTTGCATTGCATTTGC

>ZmCP36

TGCACGCACATGATCCATGTCGCAGTTGGCCTGAACTGACTAGCTAGCTACTCACACACCGCACAATGTCGAGGTGCCTGGTGCTGGCTGCGGTGTTGCTCGCCGCGCTCGCACTGGCTCCGGCGGCGCGGGGCATCCCGTTCAGCGAGCGGGACCTGGCGTCGGAGGAGAGCCTGCGAGCGCTGTACGAGCGGTGGCGCAGCCACTACCACAGGGTGTCGCCGCGGGACGGGGACGACAAGCAGCAGCAGGCGCGCCGGTTCAACGTGTTCAAGGAGAACGCGCGGTACGTCCACGAGGCGAACAGGAAGGACGGCCGGCCGTTCCGGCTGGCGCTGAACAAGTTCGCGGACATGACCACGGACGAGTTCCGGCGCACGTACGCTGGCTCCCGGACGCGCCACCACCGCGCCCAGCTGGGCGAGGCGAGGAGCTTCGCGCACGCGCAGCATGGCCGGGGCGGGAGCGGGACGACGAACCTGCCGCCCGCGGTCGACTGGCGGCTCCGCGGCGCGGTCACCGGCGTCAAGGACCAAGGCCAGTGCGGTGAGCAAGGCTAGCTGCTTGTCTGTGTGTGTGAGCGTGTGTGTGTCATCACAATTCACAACTTACAGATGGTATCTTTGTATGCATGGACACTCGTTGTTGAAGGGAGCTGCTGGGCGTTCTCGGCCATCGCGGCCGTGGAGGGCGTCAACAAGATCATGACCGGGAAGCTGGTGTCCCTGTCGGAGCAGGAGCTCGTGGACTGCGACGACGTGGACAACCAAGGCTGCGACGGCGGCCTCATGGACTACGCGTTCCAGTACATCCAGAGGAACGGAGGCGTCACCACCGAGTCCAACTACCCGTACCTTGCCGAGCAGAGGAGCTGTAACAAGGCCAAAGTACACGCGGCTAGAACCAAAATCACGTACACGTCTAAGATTAATCTAAACTACTATGTAACATATCGCTGTGCAGGAACGGTCCCATGACGTGACCATAGATGGATACGAGGACGTCCCTGCTAACAACGAAGACGCTCTCCAGAAAGCCGTCGCGAGCCAGCCAGTGGCCGTTGCAATCGAGGCTAGCGGCCAAGATTTCCAGTTCTACTCAGAGGTATATGTTACGTACGTAATTTGATGGAATAATCTGCTTTTACTTAACAAAATAAAAATTTTGTATGCTCGGTGTGCCTGCTCTGCTCTGCTCTCAGGGCGTCTTCACCGGAAGCTGCGGCACGGACCTAGACCACGGGGTCGCCGCCGTTGGGTACGGCACCACTGGAGACGGCACCAAGTACTGGACAGTGAAGAACTCGTGGGGCGAGGACTGGGGCGAGAGGGGCTACATCAGAATGCAGCGAGGCGTGCCGGACTCCCGAGGGCTGTGCGGCATCGCGATGGAGCCGTCTTACCCGACCAAAAAACCGGCAGGGCATGGCGGCGGCATCGTACAAGATCTCGGCCAGGAGCTTCATCAGGCTGACGGCGGCCGCGCATCCTACTACTGAAAGAGCAGCGTTACCATGTTCCGTTGTGTTATGCGGAATGCAATGTTAATGTTACTCTCAAGGAATTGGACCAAATCAATCTAAT

>ZmCP37

AAAGACAAACTGATATGGACGGAGGAAGCAGTAGTTAAATAATATAATAATAACCGAATGAAATGAAAGTTGCTGGTAGATCAACTCTCGACTGGTATTACATGAATGCAGGTGTACGATGTGCGTATGCCATTCAGACATTCACAGTCGGCGATGTTCCTGTCCGAGCATACATGGATCAATCATAGTATAGCTATATAGGCTATAGCTAGTACGTAGTAGTGTAGTGCGCAATTCAGAGCTCGTCGTGGCTGGAGCCGTCCTCGTCGACGACGGCGTGGACCTTGGGGTTGGGCGAGGTCTTGACGGGGTAGGATGCCTCCATGGCGATGCCGCAGTGTCCCTCCTTGGCGGCCACGTCGCGCGCCATGCGGATGTACCCCTTCTCGCCCCACTCGGGGCCCCACGAGTTCTTGACCAGCCAGTACTTGGTGCCGTCCGCGGTGACCCCGTAGCCGACCGCCGCGACGCCGTGGTCCAGCTCCGTCCCGCACCTGCCGGAGAAGACCCCCTCGGAGTAGAACTGGAAGTGCGAGCCGCTGGCCTCGATGGCGACGGACACGGGCTGGTGCGCCACCGCCTTCTTGAGCGCCGACTCGTCGTTGGCCGGCACGTCCTCGTAGCCGTCGATGGTGACGACCGGGGCCGGCGACTTCTTGCAGGACGCCTGGCGCGCTCTGTACGGGTACGCGTCCTCCGCCGCCACCCCGCCGTGCTTGGCGATGTACTGGAACGCGTAGTCCATGAGGCCCCCGTTGCAGCCGGCGTTGGCCTTGGTGTCGCAGTCCACCAGCTGCTGCTCCGACAGCGACGTCAGGTTCTTGGTCTTGATCGCGTTGATGCCCTCCACGGCGGCGATCGTCGAGAACGCCCAGCAGCTACCTGCGTGGACGACATTGGTGTTGGCATTATATATATATTCTTGTCCACAAGCATCTCTATATGTAGTATATATGTACATGCATACATATATACATACATACCGCATTGGCCCTGGTCCTTGACGTCGGTGACAGCGCCCTTCTGCCTCCAGTCGACGGAGGCCGGGACGTCGCGCGCGTCGGCGTACATGAACGACGACGCCGACGCCGAGGAGCCCTGCCTGTCGCCGCGGAACATGCGGTGGTGCGCGACCCTGGAGCCCGCGTAGTGGCGTCGGAACTCGTCGGCGGTCATGTCGCCGAAGCGGTTGAGGCGGAGCTTGTAGGGCTCGTCCCGGCGGTTGAACTCGTGGATGAGGCGCACGTTGGCCTTGAAGACGTTGAAGCGGCGCGCCTTGTCGCCCAGGTCCCGCGCCAGCGCGTGGCGGCCGCGCCAGCGCTCGTACAGCGCCCACAGCGCCTCCTCCGACGCCAGGTCCTCCGCGCCGAAGTCGACCGCGGCCGCCGGCGCCGGCGCCGCGGCCAGCAGCAGCGCGGCGGCCGCCAGCAGGACGGACGCCGCGAGCACCAGCGGCAGCGCTCTCGCCATCTCTGCTAGCTTCGCTCGCGCCGCTAGGCAGATGTAGGTACTGGCCGGGCTTCTTCTTCTAGCTCGGGGATGTGGAGCGGTACGGTATACTAAGCTGCTGATGCTATGATCAGTGATGGCTGGCTGGCTGTGTTTGTGATGATGAGGTGTTGTAGCCATGGGGACGACCGGGGAGGTCCTTATATAGTAGCGCGAGATGGAGACGCGTTGCTGCATGGAAGCAGCAACAACGCAAGGACGACGGACGGACGAGGATGACGTTGCTTGCGGGCAACAGCTTCGCGCTGGGCGCTGTGGTGATTGATGAATTGAACGACACATACATACATGAGTAACACAACAACCGTACCAGGAATGCAGGCGATGAACCGTCCGTAAGCTAGGGACGACTGCAG

>ZmCP38

TCACACACAACTCGCTCGCAACACACACACACACACAGAGAGAGAGAGAGGGGGGGGAAAGCCGGCAGCATCCATCCATCGAGATGGTGAAGAAAATGGGAGCTAGCCGAGCGCTTGTGTTGGCAGCAGCAATGCTGGCCATGGCCGTGGCGGCCAAGGGTGCCCTGCTGCTGACGGACAAGGACCTGGAGTCGGAGGAGAGCATGTGGAGTCTGTACGAGCGGTGGCGCAGCGTGCACACCGTGTCGCGGGACCTCAGGGAGAAGCAGAGCAGGTTCGAGGCGTTCAAGGCGAACGCCAGGCACATCGGCGAGTTCAACAAGAGGAAAGACGTTCCCTACAAGCTCGGCCTCAACAAGTTCGCGGACCTGACGCAGGAGGAGTTCGTCAGCAAGTACACGGGCGCCAAGGTCGTCGACTCCGAGGCCGCCGCCAGGCTCGCCAGCGGCGTGCGCGTGTCGTCCAGCGACGAGTCGCCGCCGCAGCTGGCCGCCTCCGTCGGCGACGCGCCCGACGCGTGGGACTGGAGAGACCACGGAGCCGTCACCGCCGTGAAGGACCAGGGGCAGTGCGGCAGCTGCTGGGCCTTCTCGGCAGTGGGCGCGGTGGAGAGCGTCAACGCCATCGTCACGGGCAACCTGCTCACGCTGTCGGAGCAGCAGATGCTCGACTGCTCCGGCGCCGGCGACTGCACCTACGGCGGGTACACGTACTACGCCATGCTGTACGCCATCAGTAACGGGCTTACCCTCGACCAGTGCGGCAAGACTCCCTACTACCAGCGCTACGACGCCCAGCAGCATCTACCCTGCAGATTCGACGCGGTAATAAGTTGTCTGAGCTAAGATTAAAGAGAGAGTACTACTCATTTAGTATTATATATCAATGACCAATCAATATAATCGATGTATGATTGCATGCATGCATATGCAGAAAAAGCCTCCTGTGGTGAAGATCGACAGCATGTACGTGATGAACAACGCCGACGAGGCCGCCCTGAAGCGGGCAGTGTACAAGCAGCCGGTGTCCGTGCTCATCGACGCCGGCGGCATCGGCTACTACAGCGAGGGCGTCTTCACGGGGCCGTGCGGGACGTCGCTGAACCACGCCGTGCTGCTCGTCGGCTACGGCGCCACCGCGGATGGCACCAAGTACTGGATCGTCAAGAACTCGTGGGGCGCCGACTGGGGCGAGAAGGGATATTTCCGCCTCAAGCGCGACGTCGGCACCCAGGGCGGTCTCTGTGGCATCACCATGTACCCCATTTATCCCATCAAGAACTGCCCCTGCCCGGCGGCGGCGGCTGCTGTTGCGGCCTACTAGCGGCCGGAGAGAGGGATAATATATGCATGCTTATTCATAAATAAATAAACACATGCATACATACATACATACATAGAGTGTAGTAATGTATGTGTATATGTATGCGGGCATGGTTTCAATAAAACGGTGTGTATGGTGTCCCTTGCTTATTGCAAGTTAATTAATAAAGGGTAATATCTCCCTC

>ZmCP39

CGTTAATCAATCATTCCTCTGCCCATAGACAGCATCCGTTTCCGTTGCTAATTAACACTCCATCACTTGAAGCTACAATGGTGAGAGCTGCTGAGGTCGCAACGACCATGGCGGCGGCACTAGTCGTGGTCATCGCATTGTCCACTACGCCGGCGGCAAGCGCCATTGACTACACCGAGCATGACCTGGCGTCGGAGGAGTCCCTGTGGGCACTGTACGAGCGTTGGTGTGCACACTACAACATGGCGCGCGACCTCGGCGAGAAGACCCGGCGGTTCAACTTGTTCAAGGAGAACGCGCACCGCATCTACGAGCACAACCAAGGCAATGCGACGTACACGCTGGGCCTCAACCGCTTCAGCGACATGACCGACGAGGAGTTCAGCCGCTCCCCGTACGGACGTTGCTTGTTTGCCCCGGTCCAGCGCATTTCTGACGGCGAGAACGAAGAGCTCCAGCAGATGAGGACGTGTCGTTCAACCTCACGCACGGCGGCGCCACCGCAGCGCTGGGCCTCCCGCCGTCAGTGGACTGGCGCGGGAGGTCGGTGACGCGCGTGAAGGACCAGGGGCTCACCTGCGGGAGTTGCTGGGCCTTCGCAGCGATCGCGGCGGTGGAGGGCATCAACGCCATCCGGACATGGAGCCTGGTGACGCTGTCGGAGCAGCAGCTGGTGGACTGCGACAATGTGGACCATGGCTGCGCTGGTGGTTGGATACCCTCGGCGTTGGACTTCATCGTGAGGAACCGCGGCATCGTGCCGGAGGGCACGTACCCGTACATAGGCACGCAGGGCCGTTGCCGGCACGTCATGGCCCCGCCGGTGACCATCGACGGGTACCGGCGAGTTCTGCCGTTCGACGTGAATGCACTGATGTCTGCGGTGGCTGCCCAGCCTGTGGCAGTGGCGATGGAGTCTAGCGCCTGGGCCTTCAGGCACTACCAAGGGGGCGTCTTTAATGGGAACTGCGGGGGGAGGCTGGGTCACGCGGCGGCGGTGGTGGGGTACGGCGACGGGGCCGGCGGCCCTTTCTGGATCGTCAAGAATTCATGGGGTCCCAAGTGGGGCGAGGGCGGGTACGTCCGCATCAGCCGCAACGCGCCCAACAGGCTGGGGATCTGCGGCATTCTCACACAACCGTTGTACCCTGTGAAGCGCTAGGATACCTATTTCAATAGCTATTCCGGTTTGTTCGTTGGGATAAAAAGTTTATGTATCGCATGATACAAGTAACCCGTTCCATAATAATACAATATACTCATAGTCACAAATAGTGCATATGTACCTTATATG

**Amino acid:**

>ZmCP01

MAAYQQAPALLCACLMLVLMAGAASGGRVDVEDMLMMDRFRAWQATYNRSYLTAAERLRRFEVYRQNMELIEATNRRAELSYQLSETPFTDLTSEEFLATHTMSTRLHASEAARRHRELITTHAGPVSDGGRQWNRRNYTTDLDVPESVDWRTKGAVTTVKDQGACGGCWSFATVAAIEGLHKIRTGQLVSLSEQEVLDCSSPPNNGCHGGNPAAAIDWVSANGGLTTESDYPYEGRQGKCKLDKARNHVAKIRGRKLVDQNNEAALEVAVAQQPVAVGMNVHPIQQHYKSGVFHGPCDPEDLNHAVTMVGYGAESGGRKYWIVKNSWGEKWGEKGYFRGFASRGASRTSGAPAV

>ZmCP02

MAAISLRLSTSSSQAHSKTSTMAAYQQAPALLCACLMLVLMAGAASGGRVDVEDMLMMDRFRGWQATYNRSYLTAAERLRRFEVYRQNMELIEATNRRAGLSYQLGETPFTDLTSEEFLATHTMSTRLHASEAARRHRELITTHAGPVSDGGRQWNRNYTTDLDVPESVDWRTKGAVTPVKDQGACGSCWSFVTVAAIEGLHKIRTGQLVSLSEQAVLDCSSPPNHGCNRGDPAAAIDWVSANGGLTTESDYPYVGRQGKCKLDKARNHVAKIKGRKLVDQNNEAALEVAVAQQPVAVDMNVDPILQHYKSGVFHGPCDPEDVNHGVTMVGYGVESGGRKYWIAKNSWGNEWGEKGYFRXSRGASRTPGARVV

>ZmCP03

MALFRAAASGGFALILLACCSLIMLAAASGGGGVDDDGVGGDRLMMDRFLSWQATYNRSYPTAEERQRRFQVYRRNIEHIEATNRAGNLTYTLGENQFADLTEEEFLDLYTMKGMPVRRDAGKKRANVSSSAAAVDAPTSVDWRSKGAVTPIKNQGPSCSSCWAFVTAATIESITKITTGKLVSLSEQELIDCDPYDGGCNLGYFVNGYRWVIQNGGLTTEANYPYQARRYACSRSRAAQHAATISDYVQLPAGEGQLQQAVAQQPVAAAIEMGGSLQFYSGGVFSGQCGTRMNHAITVVGYGADSSSGLKYWLVKNSWGQSWGERGYLRMRRDVGRGGLCGIALDLAYPVV

>ZmCP04

MATTSALLALVLLASLLAGTVFSDDIVPIHIPLLDRFQAWQAEYNRTYATPEEFQQRFMVYSENVKFIETMNQPGSSYELGENQFADLTEEEFKDTYLMKLDNVASSPEAMALTVDTMNRAGTSGGSNTNEAPNSVDWRTKGAVTPVKSQQHCGSCWAFAAVASIEGVHKIKTGRLVSLSEQEIVDCDRGGNNHGCHGGHSSSAMEWVTRNGGLTTESDYPYVGRQGQCMSDKLGHHAAKIRGRQAVQGKNEGALQHAVAGRPVAVSINASRAFQFYKRGIFSGPCNTTRNHAVTVVGYGANASGHKYWIVKNSWGERWGEKGYVRMQRGVRAREGVCGIAIAPFYAVM

>ZmCP05

MTMATASASLALVMLFACSLLLAGTAFSDDTIAIPLLERFKAWQAEYNRTYATPEEFQQRFMVYSENLRFIKTMNQLSTGSSYELGENQFTDLTEEEFKDTYLMKLDEQPPAAEAMPPIVGTMSTAGMSNGDNTGEAPNSVDWRTKGAVTPVKNQQQCGSCWAFATVASIEGVHQIKTGRLVSLSEQEIVDCDRGGNDHGCRGGYPRSAMEWVTRNGGLTTESDYPYVGSQRQCMSGKLGHHAARIRGYQAVQRKNEAELERAVAGRPVAVVIDASRAFQFYKRGVFSGPCNTTTVNHAVTVVGYGSAGSDSGGGRKYWIVKNSWGQRWGENGYVRMARRVRAREGMCAIAIEPYYPVM

>ZmCP06

MAGVTRLEKGQGAGWVRGVLGVEHRAGERERESISLVVLCDPEEFQQRFMVYSENVRFIKTMNQLSTGSSYELGENQFTDLTEEEFKDTYLMKLDEQPPAAEAMPPTVGTMSTAGMSNGDNTGEAPNSVDWRTKGAVTPVKNQQQCGSCWAFATVASIEGVHQIKTGRLVSLSEQQIVDCDRGGNDHGCHGGYPRSAMEWVTRNGGLTTESDYPYVGSQRQCMSGKLGHQAARIRGYQAVQRKNEAELERAVAGRPVAVVIDASRAFQFYKRGVFSGPCNTTTVNHAVTVVGYGSTGSDSGGGRKYWIVKNSWGQRWGENGYVRMARRVRAREGMCAIAIEPYYPVM

>ZmCP07

MNGPPSPSSSSRLSVALLLMITVLACGFVLASSGRSYAHADYADGSDQELLMSTEWFRFHAWMAAHGRSYPTAEEKLRRFHIYRANVELIEATNRDTSKTFTCGENQFTDLSHHEFLAMYTMAGHSAPPLLNLSSVITTRAGDITESDRGTTQVEEDEEVEALPENIDWREQNAVTPVQDQRRGCNACWVFASVATMESAHKIKTNHGHGELLKLSEQEIVDCTSQHCGGGYPDDAFSWVKRNGIATESEYGGYEATVDSCRADMVRPPAVRVKDYSFVPKNSEKKLAMRVAQQPVAVLFDATDPCFQCYTNGIYSGRPAAAADRYNILNHAMAIVGYGEDKTTGRKYWIAKNSWGTRWGQNGYVYIRKDMADRPEGVGGLATHPRYPIV

>ZmCP08

MASSSKGSLPCVLLLLAVFHHGCSSARAHRRAGDMERSMSTDDSSMIERFQRWKAAYNKSYATVAEERRRFRVCARNMAYIEATNAEAEAAGLTYELGETAYTDLTNQEFMAMYTAPAPAQLPADESVITTRAGPVDAVGGAPGQLPVYVNLSTSAPASVDWRASGAVTPVKNQGRCGSCWAFSTVAVVEGIYQIRTGKLVSLSEQELVDCDTLDDGCDGGISYRALRWIASNGGITTETDYPYTGTTDACNRAKLSHNAVSIAGLRRVATRSEASLANAVAGQPVAVSIEAGGDNFQHYKKGVYNGPCGTNLNHGVTVVGYGQEAAGGDRYWIVKNSWGQGWGDDGYIRMKKDVAGKPEGLCGIAIRPSYPLM

>ZmCP09

MARSPRLLALLLAVVWICGAALVARADPMLERFEQWMGRHGRLYADAGEKQRRLEVYRRNVELVETFNSMGNGYRLADNKFADLTNEEFRAKMLGFGRPRSGGGAGHSTAPSTVACIGSGLMGRQGYSDLPKSVDWREKGAVAPVKSQGDCGSCWAFSAVAAIEGINQIKNGKLVSLSEQELVDCDTKAIGCAGGYMSWAFEFVMKNRGLTTERNYPYQGLNGACQTPKLKESAVSISGYMNVTPSSEPDLLRAAAAQPVSVAVDAGSFVWQLYGGGVFTGPCTAELNHGVTVVGYGETQGDTDGDGSGVPGKKYWIVKNSWGPEWGDAGYILMQREASVASGLCGIAMLPSYPVM

>ZmCP10

MAHRVLLLLSLASAAAVAAAVDAEDPLIRQVVPGGDDNDLELNAESHFLSFVQRFGKSYKDADEHAYRLSVFKANLRRARRHQLLDPSAEHGVTKFSDLTPAEFRRTYLGLRKSRRALLRELGESAHEAPVLPTDGLPDDFDWRDHGAVGPVKNQGSCGSCWSFSASGALEGAHYLATGKLEVLSEQQFVDCDHECDSSEPDSCDSGCNGGLMTTAFSYLQKAGGLESEKDYPYTGSDGKCKFDKSKIVASVQNFSVVSVDEAQISANLIKHGPLAIGINAAYMQTYIGGVSCPYICGRHLDHGVLLVGYGASGFAPIRLKDKPYWIIKNSWGENWGENGYYKICRGSNVRNKCGVDSMVSTVSAVHASKE

>ZmCP11

MAPRRLLVLAVVALAATAAAANSGFADSNPIRPVTDRAASALESTVFAALGRTRDALRFARFAVRYGKSYESAAEVHKRFRIFSESLQLVRSTNRKGLSYRLGINRFADMSWEEFRATRLGAAQNCSATLTGNHRMRAAAVALPETKDWREDGIVSPVKNQGHCGSCWTFSTTGALEAAYTQATGKPISLSEQQLVDCGLAFNNFGCNGGLPSQAFEYIKYNGGLDTEESYPYQGVNGISKFKNENVGVKVLDSVNITLGAEDELKDAVGLVRPVSVAFEVITGFRLYKSGVYTSDHCGTTPMDVNHAVLAVGYGVEDGVPYWLIKNSWGADWGDEGYFKMEMGKNMCGVATCASYPIVA

>ZmCP12

MAPHIVVNKTVITFTAVALTILAVTTMMAEARDLSSTSTGGYGEEAMKVRHQQWMAEHGRTYRDEAEKAHRFQVFKANADFVDASNAAGDDKKSYRLELNEFADMTNDEFMAMYTGLRPVPAGAKKMAGFKYGNVTLSDADDDQQTVDWRQKGAVTGIKNQGQCGCCWAFAAVAAVEGIHQITTGNLVSLSEQQVLDCDTDGNNGCNGGYIDNAFQYIVGNGGLGTEDAYPYTAAQAMCQSVQPVAAISGYQDVPSGDEAALAAAVANQPVSVAIDAHNFQLYGGGVMTAASCSTPPNLNHAVTAVGYGTAEDGTPYWLLKNQWGQNWGEGGYLRLERGANACGVAQQASYPVA

>ZmCP13

MAPYIVVNKTVIAFTAVALTILAVKTMMAEARDLSSTSTGGYGEEAMKVRHQQWMAEHGRTYRDEAEKAHRFQVFKANADFVDASNAAGDDKKSYRMELNEFADMTNDEFMAMYTGLRPVPAGAKKMAGFKYGNVTLSDADDNQQTVDWRQKGAVTGIKNQGQCGCCWAFAAVAAVEGIHQITTGNLVSLSEQQVLDCDTEGNNGCNGGYIDNAFQYIAGNGGLATEDAYPYTAAQAMCQSVQPVAAISGYQDVPSGDEAALAAAVANQPVSVAIDAHNFQLYGGGVMTAASCSTPPNLNHAVTAVGYGTAEDGTPYWLLKNQWGQNWGEGGYLRLERGANACGVAQQASYPVA

>ZmCP14

MDQSNISNKHITMTTLMLLLCVIAIADCICQAAVAARVEPSTTVGRTTGGDEAMMMARYKKWMAQYRRKYKDDAEKAHRFQVFKANAEFIDRSNAGGKKKYVLGTNQFADLTSKEFAAMYTGLRKPAAVPSGAKQIPAGFKYQNFTRLDDDVQVDWRQQGAVTPVKNQGQCGCCWAFSAVGAMEGLIMITTGNLVSLSEQQILDCDESDGNQGCNGGYMDNAFQYVVNNGGVTTEDAYPYSAVQGTCQNVQPAATISGFQDLPSGDENALANAVANQPVSVGVDGGSSPFQFYQGGIYDGDGCGTDMNHAVTAIGYGADDQGTQYWILKNSWGTGWGENGFMQLQMGVGACGISTMASYPTP

>ZmCP15

MSASRFLLAVLVVGSAVLCTAAAPRALAAAAAAMASRHEKWMAEHGRAYKDEAEKARRLEVFRANAELIDSFNAAGTHSHRLATNRFADLTVQEFRAARTGLRPRPAPSAGAGRFRYENFSLADAAQSVDWRAMGAVTGVKDQGASGCCWAFSAVAAVEGLNKIRTGRLVSLSEQELVDCDVSGVDQGCDGGLMDNAFQFVARRGGLASESGYPYQCRDGPCRSSAAAAAASIRGHEDVPRNNEAALAAAVAHQPVSVAINGEDMAFRFYDSGVLGGACGTDLNHAITAVGYGTAADGTRYWLMKNSWGASWGEGGYVRIRRGVRGEGVCGLAKLPSYPV

>ZmCP16

MATHYSSAFVLLSVVAWACALSGSLAARDLADQDQAMVARHEEWMAKYDRVYSDAAEKARRFEVFKANMALIESVNAGNHKFWLEANRFADLTDDEFRATWTGYRPKTAAASSKGRSRTATTGFKYANVSLDDVPASVDWRTKGAVTPIKNQGECGCCWAFSAVASMEGVVKLSTGKLVSLSEQELVDCDVNGMDQGCEGGEMDDAFDFIVGNGGLTTESRYPYTASDGTCNSNEASGDAASIKGYEDVPANDEASLRKAVANQPVSVAVDGGDSHFRFYKGGVLSGACGTELDHGIAAVGYGVASDGTKYWVMKNSWGTSWGEAGYIRMERDIADEEGLCGLAMQPSYPTA

>ZmCP17

MVSSKAFLLLLAVLIGCVCSFPSPVLAARELSDDAAMAERHERWMAEYGRVYKDAADKARRFEVFKDNFAFVESFNADKKNKFWLGVNQFADLTTEAFKANKGFKPISAEKAPTTGFKYENLSISALPTAVDWRTKGAVTPIKNQGQCGCCWAFSAVAAVEGIVKLSTGNLVSLSEQELVDCDTHSMDEGCEGGWMDSAFEFVIKNGGLATESSYPYKAVDGKCKGGSKSAATIKGHEDVPPNNEAALMKAVASQPVSVAVDASDRTFMLYSGGVMTGSCGTQLDHGIAAIGYGVESDGTKYWIMKNSWGTTWGEKGFLRMEKDISDKRGMCGLAMKPSYPTE

>ZmCP18

MVSSRAFLLLLAILTGCACSFPSPVLAARELSDDAAMAERHERWMAVYGRVYKDAAEKARRFEVFKDNLAFVESFNADKKNKFWLGVNQFADLTTEEFKANKGFKPISAEEVPTTGFKYENLSVSALPTAVDWRTKGAVTPIKNQGQCGCCWAFSAVAAMEGIVKLSTDNLVSLSEQELVDCDTHSMDEGCEGGWMDSAFEFVIKNGGLATESSYPYKAVDGKCKGGSKSAATIKGHEDVPPNNEAALMKAVASQPVSVAVDASDRTFMLYSGGVMTGSCGTQLDHGIAAIGYGVESDGTKYWILKNSWGTTWGEKRFLRMEKDISDKQGMCGLAMKPSYPTE

>ZmCP19

MAERHERWMAEYDRVYKDAAEKARRFEVFKDNFAFVESFNADKKNKFWLGVNQFADLTTEEFKANKGFKPISAEEVPTTGFKYENLSVSALPTAVDWRTKGAVTPIKNQGQCGCCWAFSAIAAMEGIVKLSTGNLVSLSEQEPVDCDTHNMDEGCEGGWMDNAFEFVIKNGGLATESSYPYKVVDGKCKGGSKSAATIKGHEDVPPNNEAALMKVVASQPVSVAVDASDRTFMLYSGGVMTGSCGTQLDHGIAAIGYGVESDDTKYWILKNSWGTTWGEKGFLRMEKDISDKRGMCDLAMKPSYPTE

>ZmCP20

MGIPKALLLAILGCGVCLCSAAVLAARELGGDDELAMVARHEQWMVQHGRVYKDETDKAHRFLVFKANVKFIESFNAAAAAGNRKFWLGVNQFADLTNDEFRATKTNKGFNPNVVKVPTGFRYQNLSIDALPQTVDWRTKGAVTPIKDQGQCGCCWAFSAVAATEGIVKISTGKLTSLSEQELVDCDVHGEDQGCNGGEMDDAFKFIIKNGGLTTESNYPYTAQDGQCKSGSNGAATIKGYEDVPANDEAALMKAVASQPVSVAVDGGDMTFQFYSGGVMTGSCGTDLDHGIAAIGYGKTSDGTKYWLMKNSWGTTWGENGFLRMEKDIADKKGMCGLAMQPSYPTA

>ZmCP21

MATLQASILAVLSFAFFCGAALAARDLNEDSAMVARHEQWMAQYSRVYKDAAEKARRFEVFKANVKFIESFNTGGNRKFWLGINQFADLTNDEFRTTKTNKGFKPSLDKVSTGFRYENVSVDAIPATIDWRTNGAVTPIKDQGQCGCCWAFSAVAATEGIVKISTGKLISLSEQELVDCDVHGEDQGCEGGLMDDAFKFIIKNGGLTTESNYPYTAADGKCKSGSNSAANIKGYEDVPTNDEAALMKAVANQPVSVAVDGGDMTFQFYSGGVMTGSCGTDLDHGIAAIGYGKTSDGTKYWLMKNSWGTTWGENGYLRMEKDISDKKGMCGLAMEPSYPTE

>ZmCP22

MATLKASISAIIGFAFFCGAAMAARDLSDDSVMVARHEQWMAQYSRVYKDASEKARRFEVFKANVQFIESFNAGGNNKFWLGVNQFADLTNDEFRSTKTNKGLKSSNMKIPTGFRYENVSADALPTTIDWRTKGAVTPIKDQGQCGCCWAFSAVAATEGIVKISTGKLVSLAEQELVDCDVHGEDQGCEGGLMDDAFKFIIKNGGLTTESSYPYTAADGKCKSGSNSAATIKGYEDVPANDEAALMKAVANQPVSVAVDGGDMTFQFYSGGVMTGSCGTDLDHGIAAIGYGKTSDGTKYWLMKNSWGTTWGENGYLRMEKDISDKRGMCGLAMEPSYPTE

>ZmCP23

MATLKASILAILGFAFFCGAALAARDLSDDSAMVARHEQWMAQYSRVYKDASEKARRFEVFKANVKFIESFNAGGNNKFWLGVNQFADLTNDEFRSIKTNKGFKSSNMKIPTGFRYENVSVDALPTTIDWRTKGAVTPIKDQGQCGCCWAFSAVAATEGIVKISTGKLVSLAEQELVDCDVHGEDQGCEGGLMDDAFKFIINNGGLTTESSYPYTAADGKCKSGSNSAATIKGYEDVPANDEAALMKAVANQPVSVAVDGGDMTFQFYSSGVMTGSCGTDLDHGIAAIGYGKTSDGTKYWLMKNSWGTTWGENGYLRMEKDISDKRGMCGLAMEPSYPTE

>ZmCP24

MHAKMTKPAAIVVAAIAVLSVSLLAGSSCLALARPSGDFSIVGYSEEDLSSHESLAELFERWLSRHRRAYASLEEKLRRFQVFKDNLHHIDETNRKVSSYWLGLNEFADLTHDEFKATYLGLRSSVGDGGSGIDDDDEPEEEEGYEGVDGASLPKSVDWRSKGAVTGVKNQGQCGSCWAFSTVAAVEGINQIVTGNLTALSEQELIDCDTDGNNGCNGGLMDYAFSYIAHNGGLHTEEAYPYLMEEGTCQRSSSSEKKWPGSSEDANDDAAVVTISGYEDVPRNNEQALLKALAQQPVSVAIEASGRNFQFYSGGVFDGPCGTQLDHGVAAVGYGTAAKGHDYIIVKNSWGPSWGEKGYIRMRRGTGKRQGLCGINKMASYPTKN

>ZmCP25

MAWSCARPMSIALAAVLLLCGGAWLQQAAEARPHHMDDDSSIDMDRGSDDFFSIVGYSPEDLTQHDRLVRLFEEWVAKYRKAYGSFEEKLRRFEVFKDNLHHIDEANRKEVTSYWLGLNAFADLTHDEFKATYLGLLPKRTSGGRFRYGGVGDGGDEVPASVDWRKKGAVTEVKNQGQCGSCWAFSTVAAVEGINQIVTGNLTSLSEQQLVDCSTDGNNGCSGGVMDNAFSFIATGAGLRSEEAYPYLMEEGDCDDRARDGEVLVTISGYEDVPANDEQALVKALAHQPVSVAIEASGRHFQFYSGGVFDGPCGSELDHGVAAVGYGSSKGQDYIIVKNSWGTHWGEKGYIRMKRGTGKPEGLCGINKMASYPTKDH

>ZmCP26

MEPKLAVAVFVLFLAFAACSANHHRDPSVVGYSQEDLALPSSLFRSWSVKHGKLYASPTEKLERYEIFKQNLMHIAETNRKNGSYWLGLNQFADVAHEEFKASYLGLKRALPRAGAPQTRTPTAFRYAAAAAGSLPWSVDWRYKGAVTPVKNQGKCGSCWAFSSVAAVEGINQIVTGKLVSLSEQELVDCDTTLDHGCEGGTMDLAFAYMMGSQGIHAEDDYPYLMEEGYCKEKQPQADVVTLTGFEDVPENSEISLLKALAHQPVSVGIAAGSRDFQFYRGGVFDGACSVELDHALTAVGYGSSYGQNYITMKNSWGKNWGEQGYVRIKMGTGKPEGVCGIYTMASYPVKNATRWGA

>ZmCP27

MPSVHHHLLLLTLAALAVAAATASAGGDPPAIEAQFDAWCAEHGKAYATPEERAARLAVFADNAAFVAAHNARAGANAAGGGGGGAAPPSYTLALNAFADLTHEEFRAARLGRIAPGAALRSRAAPVYWGLGGGAAVPDALDWRKSGAVTKVKDQGSCGACWSFSATGAMEGINKIKTGSLVSLSEQELIDCDRSYNSGCGGGLMDYAYKFVIKNGGIDTEEDYPYREADGTCNKNKLKKRVVTIDGYTDVPSNKEDLLLQAVAQQPVSVGICGSARAFQLYYQGIFDGPCPTSLDHAVLIVGYGSEGGKDYWIVKNSWGESWGMKGYMHMHRNTGDSKGVCGINMMASFPTKTSPNPPPSPGPGPTKCSLLTYCPEGSTCCCSWRVLGFCLSWSCCELDNAVCCKDNRYCCPHDYPVCDTGRGQCLKASGNFSAIEGIRRKQSFSKAPSWTGWLELMDQ

>ZmCP28

MRPSSRYPPQPALLALALALAAAAPELRPVAAAVAVTVTPPPERTDEEVRRLYEEWRSEHDAGPRRGATGGSLGPGDADAGAGAGEDDDARRLEVFRDNLRYIDAHNAEADAGLHGFRLGLTRFADLTLEEYRARLLLGSRGRNGTAVGVVGRRRYLPLAGEQLPDAVDWRERGAVAEVKDQGQCGGCWAFSAVAAVEGINKIVTGSLISLSEQELIDCDKFQDQGCDGGLMDNAFVFMIKNGGIDTEADYPFTGHDGTCDLKLKNTRVVSIDSFERVPINYERALQKAVAHQPVSASIEASRRAFQLYSSGIFDGRCGTYLDHGVTVVGYGSEGGKDYWIVKNSWGTQWGEAGYVRMARNVRVRPPSAGIAMEPLYPVKEGPNPPPGPTPPSPVKPPNVCNAEYSCPEATTCCCVSEYRGKCLAYGCCELENATCCEDHSSCCPHDYPVCSVRDGTCRKSANSPMMVKALQRKPAMYTGGGGGGEQSGRSSW

>ZmCP29

MRPTRSAVSATALLLLAVALALAATAAARHSYTTTTTRVPAPAERADEEVRRMYEAWKSKHGRGGSSNDDCDMAPGDDEQEEEDRRLRLEVFRDNLRYIDAHNAEADAGLHTFRLGLTPFADLTLEEYRGRVLGFRARGRRSGARYGSGYSVRGGDLPDAIDWRQLGAVTEVKDQQQCGGCWAFSAVAAIEGVNAIATGNLVSLSEQEIIDCDAQDSGCDGGQMENAFRFVIGNGGIDTEADYPFIGTDGTCDASKEKNEKVATIDGLVEVASNNETALQEAVAIQPVSVAIDASGRAFQHYSSGIFNGPCGTSLDHGVTAVGYGSESGKDYWIVKNSWSASWGEAGYIRMRRNVPRPTGKCGIAMDASYPVKDTYHPGTGTATARAAAMDVIKMVLA

>ZmCP30

MAALGLLLLLLLAVVGAANAAAPGGRMSIISYNEEHAARGLERTEPEARTLYELWLAEHGRAYNALGERDRRFRVFWDNLRFVDAHNERAAEHGFRLGMNQFADLTNDEFRAAYLGARIPASRRRGTAVGERYRHGGGAEELPESVDWREKGAVAPVKNQGQCGSCWAFSAVSSVESVNQIVTGEMVTLSEQELVECSTDGGNSGCNGGLMDAAFDFIIKNGGIDTEGDYPYKAVDGKCDINRENAKVVSIDGFEDVPENDEKSLQKAVAHQPVSVAIEAGGREFQLYKAGVFTGTCTTNLDHGVVAVGYGTENGKDYWIVRNSWGAKWGEDGYIRMERNVNATTGKCGIAMMASYPTKKGANPPKPSPTPPTPPPPPVAPDNVCDENFSCAAGSTCCCAFGFRNVCLVWGCCPMEGATCCKDHASCCPPGYPVCNVRAGTCSVSKNSPLSVKALKRTLAKLNSA

>ZmCP31

MAALGRGLPLLLLLLLLAVSGAANAAAAPGGMSIITYNEEHGARGLERTEPEVRAMYDLWLAEHGRAYNALGEGEGERDRRFLVFWDNLRFVDAHNERAGARGFRLGMNQFADLTNDEFRAAYLGAMVPAARRGAVVGERYRHDGAAEELPESVDWREKGAVAPVKNQGQCGSCWAFSAVSSVESVNQIVTGEMVTLSEQELVECSTDGGNSGCNGGLMDAAFDFIIKNGGIDTEDDYPYRAVDGKCDMNRKNARVVSIDGFEDVPENDEKSLQKAVAHQPVSVAIEAGGREFQLYKSGVFSGSCTTNLDHGVVAVGYGAENGKDYWIVRNSWGPKWGEAGYIRMERNVNASTGKCGIAMMASYPTKKGANPPRPSPTPPTPPAAPDNVCDENFSCSAGSTCCCAFGFRNVCLVWGCCPVEGATCCKDHASCCPPGYPVCNVRAGTCSVSKNSPLSVKALKRTLAKLSTA

>ZmCP32

MAASTTAAAAASLLLLLLVSLAAAADMSIVSYGERSDEEARRMYAEWMAAHGRTYNAVGEEERRYQVFRDNLRYIDAHNAAADAGVHSFRLGLNRFADLTNDEYRATYLGARTRPQRERKLGARYHAADNEDLPESVDWRAKGAVAEVKDQGSYGSCWAFSTIAAVEGINQIVTGDLISLSEQELVDCDTSYNQGCNGGLMDYAFEFIINNGGIDTEKDYPYKGTDGRCDVNRKNAKVVTIDSYEDVPANDEKSLQKAVANQPVSVAIEAAGTQFQLYSSGIFTGSCGTALDHGVTAVGYGTENGKDYWIVKNSWGSSWGESGYVRMERNIKASSGKCGIAVEPSYPLKEGANPPNPGPSPPSPTPAPAVCDNYYSCPDSTTCCCIYEYGKYCFAWGCCPLEGATCCDDHYSCCPHDYPICNVRQGTCLMGKDSPLLSVKATKRTLAKPHWAFSGNTADGMKSSA

>ZmCP33

MGASTTPLASAAALLLLLLAPLAAAADSMSIVSYGERSEEEARRMYAEWMAAHGRTYNAVGEEERRFEVFRDNLRYVDAHNAAADAGVHSFRLGLNRFADLTNDEYRATYLGVRSRPQRERRLGDRYLAGDNEDLPESVDWRAKGAVAEVKDQGSCGSCWAFSTIAAVEGINQIVTGDMISLSEQELVDCDTSYNQGCNGGLMDYAFEFIINNGGIDTEEDYPYKGTDGRCDVNRKNAKVVTIDSYEDVPANSEKSLQKAVANQPISVAIEAGGRAFQLYNSGIFTGTCGTALDHGVTAVGYGTENGKDYWIVKNSWGSSWGESGYVRMERNIKASSGKCGIAVEPSYPLKKGANPPNPGPTPPSPTPPPTVCDNYYSCPDSTTCCCIYEYGKYCFAWGCCPLEGATCCDDHYSCCPHDYPVCNVKQGTCLMGKDSPLSLSVKATKRTLAKPHWAFPGNTAADGMKSSA

>ZmCP34

MAQVSKTLLLVALVFVSSAAVELCRAIDFDERDLASDEALWDLYERWQTHHRVHRHHGEKGRRFGTFKENVRFIHAHNKRGDRPYRLRLNRFGDMGREEFRSTFADSRINDLRRQDSPAARAGAVPGFMYDSAADPPRSVDWRQEGAVTGVKDQGHCGSCWAFSTVVAVEGINAIRTGSLASLSEQELIDCDTDENGCQGGLMENAFEFIKSFGGITTEAAYPYRASNGTCDGDRARRGGGVVVVIDGHQMVPAGSEDALAKAVAHQPVSVAVDAGGQAFQFYSEGVFTGDCGTDLDHGVAAVGYGVGDDGTPYWIVKNSWGTSWGEGGYIRMQRGAGNGGLCGIAMEASFPIKTSPNPADPPRKPRRALIARDTSSSQ

>ZmCP35

MAQVAKTLLLVALVVVSAVELCRAIEFDERDLASDEALWDLYERWQTHHRVHRHHGEKGRRFGTFKENARFIHAHNKRGDRPYRLRLNRFGDMGREEFRSGFADSRINDLRREPTAAPAVPGFMYDDATDLPRSVDWRQKGAVTAVKNQGRCGSCWAFSTVVAVEGINAIRTGSLVSLSEQELIDCDTDENGCQGGLMENAFEFIKSHGGITTESAYPYHASNGTCDGARARRGRVVAIDGHQAVPAGSEDALAKAVAHQPVSVAIDAGGQALQFYSEGVFTGDCGTDLDHGVAAVGYGVSDDGTPYWIVKNSWGPSWGEGGYIRMQRGTGNGGLCGIAMEASFPIKTSPNPSRKPRRALITRDASSQ

>ZmCP36

MSRCLVLAAVLLAALALAPAARGIPFSERDLASEESLRALYERWRSHYHRVSPRDGDDKQQQARRFNVFKENARYVHEANRKDGRPFRLALNKFADMTTDEFRRTYAGSRTRHHRAQLGEARSFAHAQHGRGGSGTTNLPPAVDWRLRGAVTGVKDQGQCGSCWAFSAIAAVEGVNKIMTGKLVSLSEQELVDCDDVDNQGCDGGLMDYAFQYIQRNGGVTTESNYPYLAEQRSCNKAKERSHDVTIDGYEDVPANNEDALQKAVASQPVAVAIEASGQDFQFYSEGVFTGSCGTDLDHGVAAVGYGTTGDGTKYWTVKNSWGEDWGERGYIRMQRGVPDSRGLCGIAMEPSYPTKKPAGHGGGIVQDLGQELHQADGGRASYY

>ZmCP37

MARALPLVLAASVLLAAAALLLAAAPAPAAAVDFGAEDLASEEALWALYERWRGRHALARDLGDKARRFNVFKANVRLIHEFNRRDEPYKLRLNRFGDMTADEFRRHYAGSRVAHHRMFRGDRQGSSASASSFMYADARDVPASVDWRQKGAVTDVKDQGQCGSCWAFSTIAAVEGINAIKTKNLTSLSEQQLVDCDTKANAGCNGGLMDYAFQYIAKHGGVAAEDAYPYRARQASCKKSPAPVVTIDGYEDVPANDESALKKAVAHQPVSVAIEASGSHFQFYSEGVFSGRCGTELDHGVAAVGYGVTADGTKYWLVKNSWGPEWGEKGYIRMARDVAAKEGHCGIAMEASYPVKTSPNPKVHAVVDEDGSSHDEL

>ZmCP38

MVKKMGASRALVLAAAMLAMAVAAKGALLLTDKDLESEESMWSLYERWRSVHTVSRDLREKQSRFEAFKANARHIGEFNKRKDVPYKLGLNKFADLTQEEFVSKYTGAKVVDSEAAARLASGVRVSSSDESPPQLAASVGDAPDAWDWRDHGAVTAVKDQGQCGSCWAFSAVGAVESVNAIVTGNLLTLSEQQMLDCSGAGDCTYGGYTYYAMLYAISNGLTLDQCGKTPYYQRYDAQQHLPCRFDAKKPPVVKIDSMYVMNNADEAALKRAVYKQPVSVLIDAGGIGYYSEGVFTGPCGTSLNHAVLLVGYGATADGTKYWIVKNSWGADWGEKGYFRLKRDVGTQGGLCGITMYPIYPIKNCPCPAAAAAVAAY

>ZmCP39

MVRAAEVATTMAAALVVVIALSTTPAASAIDYTEHDLASEESLWALYERWCAHYNMARDLGEKTRRFNLFKENAHRIYEHNQGNATYTLGLNRFSDMTDEEFSRSPYGRCLFAPVQRISDGENEELQQHEDVSFNLTHGGATAALGLPPSVDWRGRSVTRVKDQGLTCGSCWAFAAIAAVEGINAIRTWSLVTLSEQQLVDCDNVDHGCAGGWIPSALDFIVRNRGIVPEGTYPYIGTQGRCRHVMAPPVTIDGYRRVLPFDVNALMSAVAAQPVAVAMESSAWAFRHYQGGVFNGNCGGRLGHAAAVVGYGDGAGGPFWIVKNSWGPKWGEGGYVRISRNAPNRLGICGILTQPLYPVKR
